# Supplementary material for: Associations between hospital deaths (HSMR), readmission and length of stay (LOS): a longitudinal assessment of performance results and facility characteristics of teaching and large-sized hospitals in Canada between 2013–2014 and 2017–2018
Source: BMJ Open. 2021 Feb 5;11(2):e041648. doi: 10.1136/bmjopen-2020-041648 (PMC7925915; doi:10.1136/bmjopen-2020-041648)
Supplement: Supplementary data [file bmjopen-2020-041648supp001.pdf]

Supplementary file

Provincial/territorial range of % change difference (2013-14 vs. 2017-18), mean % change (and 95% Confidence Intervals), combined Teaching and Community-Large hospitals

Province/territory	Indicator	Range of % change (2013-14 vs. 2017-18)	Mean % change (95% CI)
Alberta	All Patients Readmitted to Hospital	-6 to 17	3.1 (-0.7 to 6.9)
	Hospital Deaths (HSMR)	-21 to 22	-0.7 (-9.3 to 7.9)
British Columbia	All Patients Readmitted to Hospital	-12 to 12	1.9 (-1.2 to 5)
	Hospital Deaths (HSMR)	-33 to 11	-6.5 (-11.4 to -1.6)
Manitoba	All Patients Readmitted to Hospital	3 to 10	6.7 (-2.1 to 15.4)
	Hospital Deaths (HSMR)	-13 to 12	-1.3 (-32.6 to 29.9)
New Brunswick	All Patients Readmitted to Hospital	-8 to 2	-3.2 (-11.2 to 4.7)
	Hospital Deaths (HSMR)	-11 to 10	-2.5 (-16.8 to 11.8)
Newfoundland and Labrador	All Patients Readmitted to Hospital	1 to 10	5.5 (-51.7 to 62.7)
	Hospital Deaths (HSMR)	-6 to 6	0.0 (-76.2 to 76.2)
Nova Scotia	All Patients Readmitted to Hospital	-4 to 11	3.5 (-91.8 to 98.8)
	Hospital Deaths (HSMR)	1 to 21	11.0 (-116.1 to 138.1)
Ontario	All Patients Readmitted to Hospital	-14 to 9	0.9 (-1 to 2.8)
	Hospital Deaths (HSMR)	-24 to 8	-5.8 (-9.2 to -2.5)
Prince Edward Island*	All Patients Readmitted to Hospital	-5 to -5	N/A
	Hospital Deaths (HSMR)	-22 to -22	N/A
Quebec	All Patients Readmitted to Hospital	1 to 9	4.8 (1.8 to 7.8)
	Hospital Deaths (HSMR)	-21 to -1	-12.0 (-19.5 to -4.5)
Saskatchewan	All Patients Readmitted to Hospital	-2 to 3	0.8 (-2.5 to 4)
	Hospital Deaths (HSMR)	-11 to -5	-7.8 (-11.7 to -3.8)

*Only one hospital value.

Subset of hospitals (n=81), with both Readmission and Hospital Deaths (HSMR) values, used in performance trends over time analysis

Provincial/territorial jurisdiction	Community — large hospitals	Teaching hospitals	Jurisdiction total
Alberta	4	7	11
British Columbia	11	6	17
Manitoba	1	2	3
New Brunswick	3	1	4
Newfoundland and Labrador	1	1	2
Nova Scotia	1	1	2
Ontario	21	10	31
Prince Edward Island	1	0	1
Quebec	2	4	6
Saskatchewan	0	4	4
**Total**	**45**	**36**	**81**

Facility characteristic averages by hospital peer-groups

		Mean value, (n of hospitals)	
**Facility characteristic**	**Unit**	**Teaching hospitals**	**Community – Large hospitals**
**Number of Acute Care Hospital Stays**	# of days	27,322 (n=53)	20,421 (n=66)
**Number of Acute Care Beds**	# of beds	474 (n=53)	328 (n=66)
**Number of Emergency Department Visits**	# of visits	83,441 (n=40)	86,962 (n=43)
**Average Acute Care Resource Intensity Weight (RIW)**	average RIW	1.6 (n=53)	1.2 (n=66)
**Total Acute Care RIW**	total RIW	43,295 (n=53)	25,057 (n=66)
**Hospital Occupancy Rate**	% of occupancy	88.9 (n=44)	89.9 (n=61)
**Patients Admitted Through the Emergency Department (%)**	% of patients	44.4 (n=53)	54.4 (n=66)
**Patient Days in Alternate Level of Care (Percentage)**	%	11.4 (n=43)	15.4 (n=53)

Correlation matrix (scatterplot) of both Teaching and Community-Large hospitals

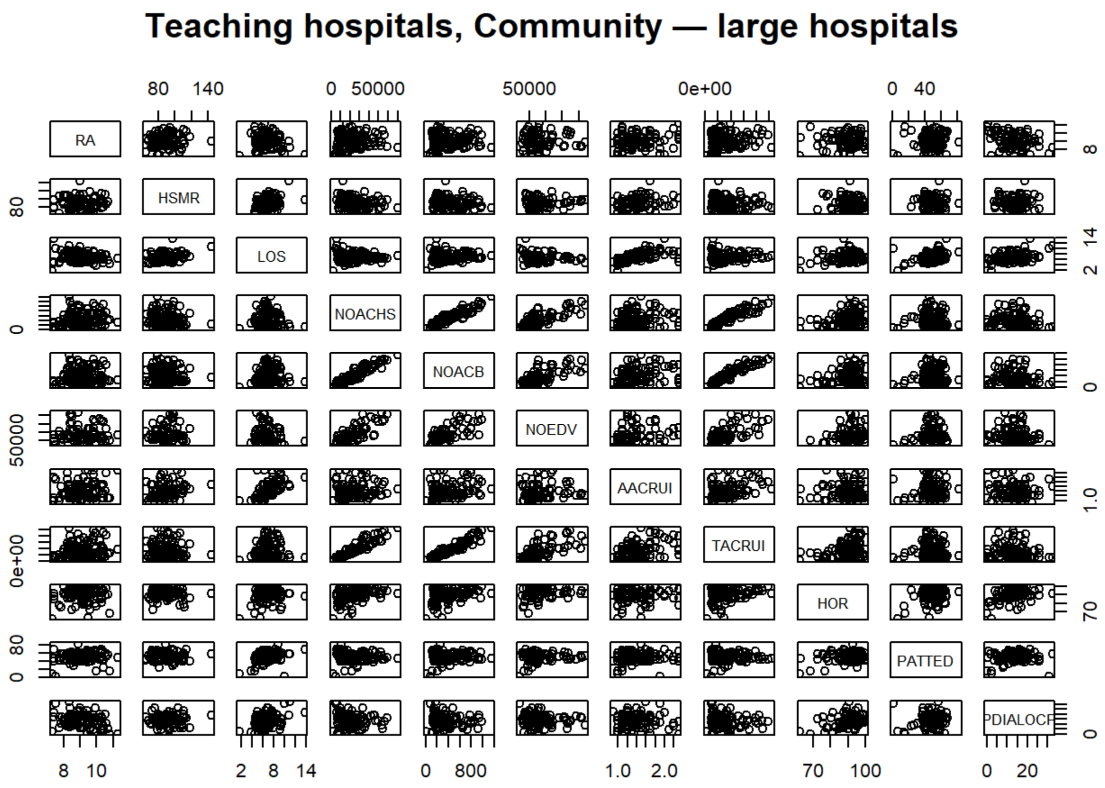

Indicator acronyms: All Patients Readmitted to Hospital (RA); Hospital Deaths (HSMR); Average Length of Stay (LOS); Number of Acute Care Hospital Stays (NOACHS); Number of Acute Care Beds (NOACB); Number of Emergency Department Visits (NOEDV); Average Acute Care Resource Intensity Weight (AACRUI); Total Acute Care Resource Intensity Weight (TACRUI); Hospital Occupancy Rate (HOR); Patients Admitted Through the Emergency Department (%) (PATTED); Patient Days in Alternate Level of Care (Percentage) (PDIALOCP).

<u>Correlation matrix (scatterplot) of Teaching hospitals</u>

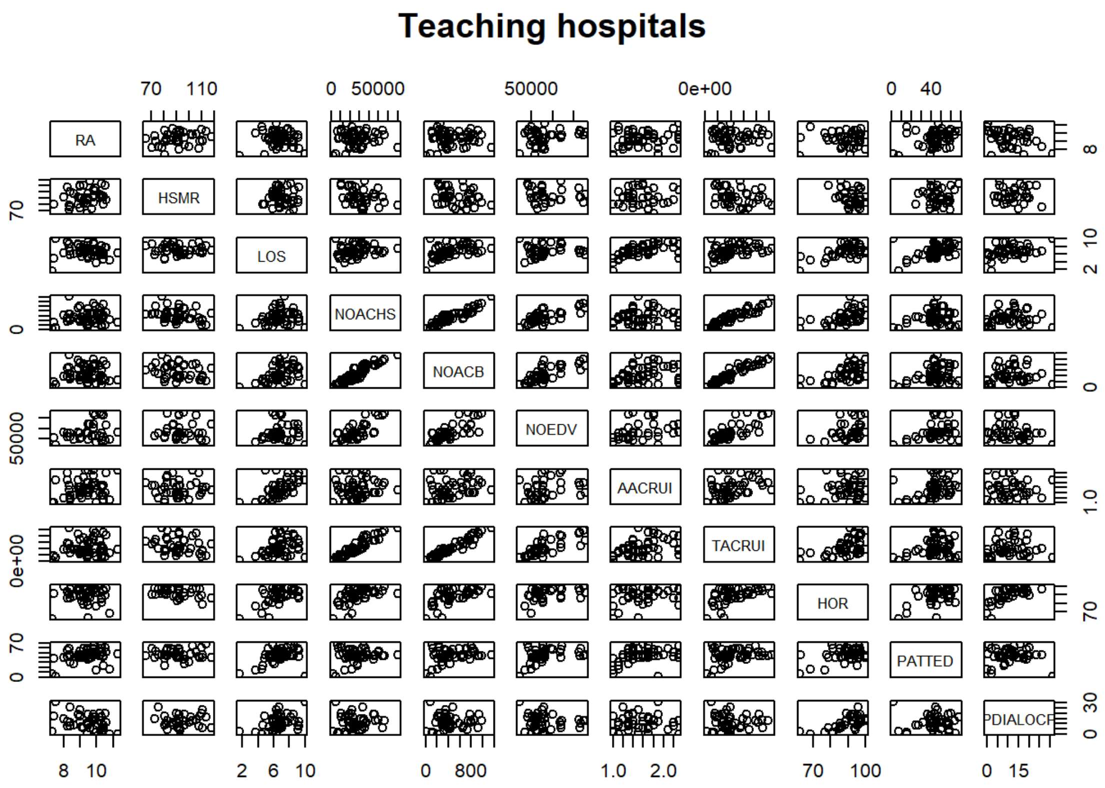

Indicator acronyms: All Patients Readmitted to Hospital (RA); Hospital Deaths (HSMR); Average Length of Stay (LOS); Number of Acute Care Hospital Stays (NOACHS); Number of Acute Care Beds (NOACB); Number of Emergency Department Visits (NOEDV); Average Acute Care Resource Intensity Weight (AACRUI); Total Acute Care Resource Intensity Weight (TACRUI); Hospital Occupancy Rate (HOR); Patients Admitted Through the Emergency Department (%) (PATTED); Patient Days in Alternate Level of Care (Percentage) (PDIALOCP).

<u>Correlation matrix (scatterplot) of Community-Large hospitals</u>

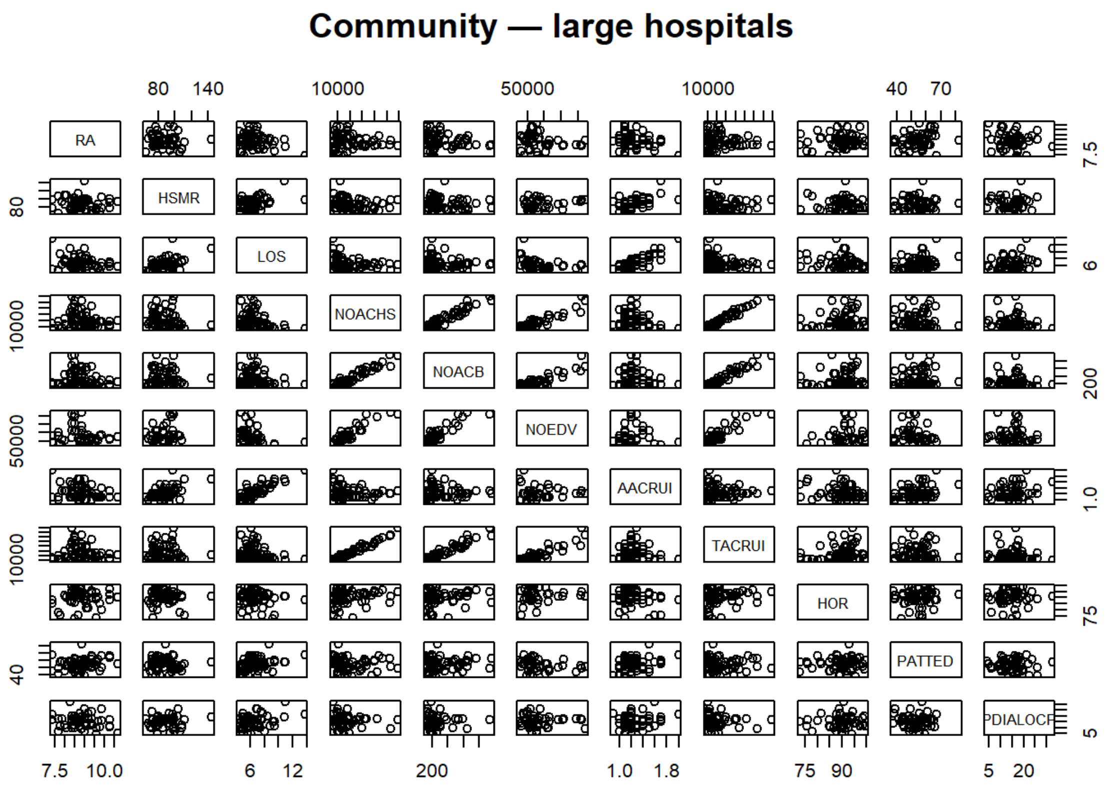

Indicator acronyms: All Patients Readmitted to Hospital (RA); Hospital Deaths (HSMR); Average Length of Stay (LOS); Number of Acute Care Hospital Stays (NOACHS); Number of Acute Care Beds (NOACB); Number of Emergency Department Visits (NOEDV); Average Acute Care Resource Intensity Weight (AACRUI); Total Acute Care Resource Intensity Weight (TACRUI); Hospital Occupancy Rate (HOR); Patients Admitted Through the Emergency Department (%) (PATTED); Patient Days in Alternate Level of Care (Percentage) (PDIALOCP).
