# Supplementary material for: Associations between hospital deaths (HSMR), readmission and length of stay (LOS): a longitudinal assessment of performance results and facility characteristics of teaching and large-sized hospitals in Canada between 2013–2014 and 2017–2018
Source: BMJ Open. 2021 Feb 5;11(2):e041648. doi: 10.1136/bmjopen-2020-041648 (PMC7925915; doi:10.1136/bmjopen-2020-041648)
Supplement: Author's manuscript [file bmjopen-2020-041648.draft_revisions.pdf]

BMJ Open is committed to open peer review. As part of this commitment we make the peer review history of every article we publish publicly available.

When an article is published we post the peer reviewers' comments and the authors' responses online. We also post the versions of the paper that were used during peer review. These are the versions that the peer review comments apply to.

The versions of the paper that follow are the versions that were submitted during the peer review process. They are not the versions of record or the final published versions. They should not be cited or distributed as the published version of this manuscript.

BMJ Open is an open access journal and the full, final, typeset and author-corrected version of record of the manuscript is available on our site with no access controls, subscription charges or pay-per-view fees (http://bmjopen.bmj.com).

If you have any questions on BMJ Open's open peer review process please email info.bmjopen@bmj.com

BMJ Open

Associations between Hospital Deaths (HSMR), Readmission and Length of Stay (LOS): a longitudinal assessment of performance results and facility characteristics of teaching and large-sized hospitals in Canada between 2013-14 to 2017-18

Journal:	*BMJ Open*
Manuscript ID	bmjopen-2020-041648
Article Type:	Original research
Date Submitted by the Author:	13-Jun-2020
Complete List of Authors:	Fekri, Omid; Academic Medical Center,<br>Manukyan, Edgar; University of Amsterdam, Academic Medical Center<br>Klazinga, Niek; Academic Medical Centre at the University of Amsterdam
Keywords:	Quality in health care < HEALTH SERVICES ADMINISTRATION & MANAGEMENT, Organisation of health services < HEALTH SERVICES ADMINISTRATION & MANAGEMENT, Clinical governance < HEALTH SERVICES ADMINISTRATION & MANAGEMENT

SCHOLARONE™
Manuscripts

I, the Submitting Author has the right to grant and does grant on behalf of all authors of the Work (as defined in the below author licence), an exclusive licence and/or a non-exclusive licence for contributions from authors who are: i) UK Crown employees; ii) where BMJ has agreed a CC-BY licence shall apply, and/or iii) in accordance with the terms applicable for US Federal Government officers or employees acting as part of their official duties; on a worldwide, perpetual, irrevocable, royalty-free basis to BMJ Publishing Group Ltd ("BMJ") its licensees and where the relevant Journal is co-owned by BMJ to the co-owners of the Journal, to publish the Work in this journal and any other BMJ products and to exploit all rights, as set out in our licence.

The Submitting Author accepts and understands that any supply made under these terms is made by BMJ to the Submitting Author unless you are acting as an employee on behalf of your employer or a postgraduate student of an affiliated institution which is paying any applicable article publishing charge ("APC") for Open Access articles. Where the Submitting Author wishes to make the Work available on an Open Access basis (and intends to pay the relevant APC), the terms of reuse of such Open Access shall be governed by a Creative Commons licence – details of these licences and which Creative Commons licence will apply to this Work are set out in our licence referred to above.

Other than as permitted in any relevant BMJ Author's Self Archiving Policies, I confirm this Work has not been accepted for publication elsewhere, is not being considered for publication elsewhere and does not duplicate material already published. I confirm all authors consent to publication of this Work and authorise the granting of this licence.

For peer review only

Title page

Date: 13 June 2020

Manuscript title:

Associations between Hospital Deaths (HSMR), Readmission and Length of Stay (LOS): a longitudinal assessment of performance results and facility characteristics of teaching and large-sized hospitals in Canada between 2013-14 to 2017-18

Authors:

Omid Fekri (corresponding author: o.fekri@amsterdamumc.nl)

Edgar Manukyan

Niek Klazinga

Affiliation: University Medical Centers - University of Amsterdam

Word count: 4310 (excluding tables, graphs, abstract, references)

For peer review only

Associations between Hospital Deaths (HSMR), Readmission and Length of Stay (LOS): a longitudinal assessment of performance results and facility characteristics of teaching and large-sized hospitals in Canada between 2013-14 to 2017-18

Abstract

Objectives: To examine the association between Hospital Deaths (HSMR), Readmission, Length of Stay (LOS), and eight hospital characteristics.

Design: Longitudinal observational study.

Setting: A total of 119 teaching and large-sized hospitals in Canada between fiscal years 2013–14 and 2017-18.

Participants: Analysis focused on indicator results and characteristics of individual Canadian hospitals.

Primary and secondary outcomes: Hospital Deaths (HSMR); All Patients Readmitted to Hospital; Average Length of Stay (LOS); and a series of eight hospital characteristic summary measures: Number of Acute Care Hospital Stays; Number of Acute Care Beds; Number of Emergency Department Visits; Average Acute Care Resource Use Intensity; Total Acute Care Resource Use Intensity; Hospital Occupancy Rate; Patients Admitted Through the Emergency Department (%); Patient Days in Alternate Level of Care (%).

Results: Comparing 2013-14 to 2017-18, Hospital deaths (HSMR) largely declined, while readmissions increased; 69% of hospitals decreased their hospital deaths (HSMR), while 65% of hospitals increased their readmissions rates. LOS was moderately positively correlated with hospital deaths (HSMR) in Community-Large hospitals ($r=0.4$, $p <0.01$). LOS was largely positively and statistically significantly correlated with the suite of eight hospital characteristics. Hospital deaths (HSMR) was largely negatively (not statistically significantly) correlated with the hospital characteristics. Readmission was largely not statistically significantly correlated. There was no clear pattern of correlation between readmissions and the hospital characteristics (with only minimal statistical significance). A greater proportion of Community-Large hospitals (31%, n=14) improved on both hospital deaths (HSMR) and readmission compared to Teaching hospitals (13.9%, n=5).

Conclusions: Examining publicly-reported hospital performance results can reveal meaningful insights into the association among outcome indicators and hospital characteristics. Good or bad hospital performance in one care domain does not necessarily reflect similar performance in other care domains. Thus, caution is warranted in a narrow use of outcome indicators in the design and operationalization of hospital performance measurement and governance models (namely pay-for-performance schemes). Analysis such as this can also inform quality-improvement strategies and targeted efforts to address domains of care experiencing declining performance over time; further granular subdivision of the analyses, for example by hospital peer-groups, can reveal notable differences in performance.

Article Summary - Strengths and limitations of this study

- Assessed correlations across eight hospital characteristics and three hospital performance indicators.

For peer review only

- Assessed five years of performance data.
- Examined the majority of Teaching and Community-Large hospitals in Canada.
- Limitation: LOS is an aggregate of all hospitalizations, and could not be restricted to condition-specific cases (of hospital death or readmission).

For peer review only

Introduction

Over the last two decades, there has been substantial interest in hospital performance[1], and with financing of hospitals increasingly tied to improving the quality of care delivered[2]. Along with improving the quality of care, a tandem goal of hospital reforms has been to improve efficiency[3] (i.e., reducing waste, streamlining care pathways, increasing patient throughput, optimizing the use of technology, etc.). Hospital deaths[4] and readmission to hospital[5] are among the most commonly used indicators to measure quality of hospital care, while average Length of Stay (LOS) is often used as a measure of efficiency[6]. The three measures together (hospital deaths, readmission and LOS) have been the subject of increased interest in recent years to assist with more reliable interpretations of hospital performance[7].

However, the goals of achieving quality and efficiency can at times be opposing. For example, it seems warranted to investigate whether a hastened hospital stay (shorter LOS) would lead to an increased chance of readmission to hospital[8]. Similarly, do efforts to reduce hospital readmissions have the unintended consequence of increasing the likelihood of mortality after hospitalization[9]? While hospital deaths and readmission are both desired to be reduced, it is not definite (and varying across diseases and clinical procedures) whether a patient's LOS should be lower or higher in order to minimize readmission or in-hospital mortality. However, what can be deduced is that the relationships between LOS, in-hospital mortality and readmission are intertwined and interdependent. Hence governance of hospitals based on these publicly reported indicators should be based on acknowledgment and consideration of these interdependencies.

Yet, despite a sizeable research community investigating the interrelationship between these indicators, the evidence-base on the patterns of these interdependencies remains inconclusive due to wide heterogeneity in methods and findings across studies (which speaks to the complexity of the topic). For example, a switch between the unit of analysis (from patient-level to hospital-level), on the same underlying admissions data, will yield inconsistent, and even inverse, results[10]. In recent years, researchers have also examined hospital characteristics, such as hospital volumes[11] or hospital teaching status[12] to better understand any associations between LOS, readmission and in-hospital mortality.

Much of the afore cited literature originates from the United States and Europe. With a scarcity of local examples, this study will use a large, nationally-representative dataset of hospital performance measures (produced by the Canadian Institute for Health Information (CIHI)) to expand interest and add evidence for the Canadian context. Specifically, we investigate the relationship between hospital deaths, readmission and LOS, and explore any associations with hospital characteristics. Our specific research questions are:

1. What are the performance trends in hospital deaths (HSMR) and readmission over time?
2. What is the correlation between hospital deaths (HSMR), readmissions and LOS?
3. How do a series of eight hospital characteristics correlate with hospital deaths (HSMR), readmissions and LOS?
4. Do the results of the aforementioned research questions show differences between peer groups of Teaching hospitals and Community-Large hospitals?

For peer review only

Methods

Data

We used the all data export report file from CIHI's Your Health System In Depth online tool[13] to perform the analyses. The data file contains results per hospital for all indicators published on the online tool as well as contextual measures and additional variables to assist with analysis and interpretation. Five singleton fiscal year (1 April to 31 March) data points were available covering 2013–14 to 2017–18 for the indicators capturing Hospital Deaths (HSMR) and All Patients Readmitted to Hospital (henceforth referred to 'Readmission'), while LOS and eight hospital characteristics measures were only available for the most recent year (2017-18).

Definition of variables

The following indicators were used for the analysis: Hospital Deaths (HSMR) (Hospital Standardized Mortality Ratio), Readmission (%), and LOS (days); and eight contextual measures of hospital facility characteristics: Number of Acute Care Hospital Stays; Number of Acute Care Beds; Number of Emergency Department Visits; Average Acute Care Resource Use Intensity; Total Acute Care Resource Use Intensity; Hospital Occupancy Rate; Patients Admitted Through the Emergency Department; Patient Days in Alternate Level of Care (%).

HSMR (hospital standardized mortality ratio) and other variations of summary hospital mortality measures are commonly-used indicators to assess hospital performance. The Hospital deaths (HSMR) indicator is a ratio of observed to expected in-hospital mortality, capturing the 72 leading causes of hospital death (representing ~80% of all in-hospital mortality). The Readmission indicator captures all urgent patient readmissions within 30-days. The average LOS indicator is a sum of all valid days spent in hospital, divided by the total number of inpatient cases. Detailed technical notes on these indicators[14], and on hospital facility characteristics[15], are made available by CIHI through its Indicator Library.

CIHI classifies the approximately 600 hospitals in Canada into four distinct peer-group types: Teaching hospitals; Community—Large hospitals; Community—Medium hospitals; and Community—Small hospitals. This classification facilitates meaningful comparisons across hospitals of similar structural characteristics, patient volume, and clinical complexity[16]. A hospital is designated as 'Teaching' by provincial/territorial ministries of health, or were identified as such in the provincial/territorial ministry's submission to CIHI's Management Information System (MIS) Database. Community—Large hospitals meet two of the following three criteria: more than 8,000 inpatient cases; more than 10,000 weighted cases; or more than 50,000 inpatient days.

In order to qualify for public-reporting of results for the Hospital Deaths (HSMR) indicator, a hospital must meet a minimum of 2,500 eligible Hospital Deaths (HSMR) cases for each of the most recent three consecutive years[17]. Consequently, no Community—Small hospitals met this criteria to have publicly-reported Hospital deaths (HSMR) results. Of the 93 Community—Medium hospitals only 11 hospitals met the minimum reporting requirements and had Hospital deaths (HSMR) results reported. Since this represents only 8.5% of the entire peer-group, it was decided to also exclude Community—Medium hospitals, alongside Community—Small hospitals, in this analysis. Hospitals with only one year of data available, for both Readmission and Hospital Deaths (HSMR) indicators, for either 2013-14 or 2017-18 only, were excluded from performance trend analysis. Therefore, a total of 119 hospitals were included in the overall study, and a subset of 81 hospitals were included in the performance trend analysis.

For peer review only

Statistical analyses

Descriptive statistics for the analysis of LOS, Hospital Deaths (HSMR) and Readmission indicators are presented by range of values, peer-group means and 95% confidence intervals (CI), and coefficient of variation (CoV) (see Table 1). Trend over time is calculated as the percent-change difference between first and last year of data (2013-14 and 2017-18). A paired-t test was used to determine whether absolute changes in rates between 2013-14 and 2017-18 were significant.

To compare indicator rates per hospital across 2013-14 to 2017-18, three possible outcomes are inferred: a decrease in rate (2013-14 > 2017-18); an increase in rate (2013-14 < 2017-18); and no change in rate (2013-14 = 2017-18). Multiplying these three outcomes by the two indicators of interest (Hospital Deaths (HSMR) and Readmission), in tandem, yields a total of nine trend outcomes (see Table 2).

Graphical representation of the aforementioned tests are shown via scatterplots depicting: 1) percent-change over time for Hospital Deaths (HSMR) and Readmission (delineated by peer-group) (see Figure 1); and 2) 2017-18 data year results on Hospital Deaths (HSMR) and Readmission, with LOS depicted as the size of the bubble plot (see Figures 2 & 3).

A Spearman's Rank Correlation test examines the association between LOS, Hospital Deaths (HSMR) and Readmission on 2017-18 data year values (with breakdowns for Teaching and Community—Large hospital peer-groups). Strengths of correlations, the absolute value of R_s (positive and negative) are defined as: .00-.19 very weak; .20-.39 weak; .40-.59 moderate; .60-.79 strong; .80-1.0 very strong[18].

Lastly, a Spearman's Rank Correlation test was also used to assess the correlation between eight hospital facility characteristics against LOS, Hospital Deaths (HSMR) and Readmission values for 2017-18. All analyses were performed on R version 3.5.0 (R Foundation for Statistical Computing, Vienna, Austria).

Patient and public involvement

Patients or public were not involved in the design of this longitudinal, observational study. However, all data used are available in the public domain.

For peer review only

Results

Performance trends of hospital mortality (HSMR) and readmission over time

In comparing 2013-14 and 2017-18 national indicator rates, Hospital deaths (HSMR) largely declined, while readmissions increased (see table 1). For both indicators, the Community-Large hospital peer-group showed greater improvement than Teaching hospitals. Community-Large hospitals on average improved on in hospital deaths (HSMR) by -6.0% (95% CI -9.1 – -2.8) compared to Teaching hospitals at -4.1% (95% CI -7.5 – -0.8). Similarly, Community-Large hospitals, while increasing in readmission rates on average 1.6% (95% CI -0.3 – 3.4), had a more favourable rate than the average for Teaching hospitals at 2.1% (95% CI 0.7 – 3.6). Furthermore, for the 2017-18 data year, Community-Large hospitals had lower average rates across all three indicators of LOS, hospital deaths (HSMR), and readmission. A paired-t test showed statistically significant changes in trend over time for both indicators: hospital deaths (HSMR) improved by a mean of -5.1 (95% CI, -7.33 – -2.9, t=-4.58, df=80, p<.001). And readmission worsened by a mean of 0.15% (95% CI, 0.04 – 0.26, t=2.81, df=80, p=.006).

Table 2 provides a lens on how individual hospitals performed in both indicators. Nine possible outcomes of performance are shown. Overall, 56 (69%) out of the total 81 hospitals assessed decreased their hospital deaths (HSMR), while only 23 (28%) hospitals decreasing their readmissions rates.

Figure 1 illustrates the combined percent change of hospital deaths (HSMR) and readmissions rates (comparing 2013-14 and 2017-18 individual hospital rates) delineated by hospital peer group. While coefficient of variation values are largely similar between the two peer groups, nearly three times as many Community-Large hospitals (n=14) showed greater improvement in the bottom left quadrant of Figure 1 (decrease in both hospital deaths (HSMR) and readmission), than Teaching hospitals (n=5). These clear trends of overall decreasing hospital deaths and rising readmissions have been confirmed in our previous analysis[19].

For peer review only

Table 1 Descriptive statistics for combined analysis of hospital deaths (HSMR), readmission and LOS

	Teaching hospitals			Community-large hospitals		
**Number of hospitals, n**	36			45		
**Range of values for 2017-18 data year**	**Range of values**	**Teaching Peer-group mean* (95%CI)**	**Coefficient of variation (%)**	**Range of values**	**Community-large Peer-group mean* (95%CI)**	**Coefficient of variation (%)**
**LOS (days)**	4.6 – 9.2	7.1 (6.7 – 7.4)	16	4.5 – 13.7	6.5 (6.1 – 6.9)	24
**Hospital Deaths (HSMR)**	66 – 118	91.8 (87.8 – 95.7)	14	65 – 144	87.5 (83.9 – 91)	16
**Readmission (%)**	7.4 – 10.6	9.4 (9.2 – 9.6)	8	7.4 – 10.7	8.9 (8.7 – 9.1)	8
**Percent-change difference 2013-14 vs. 2017-18 (%)**	**Range of % change**	**Average Teaching Peer-group % change* (95%CI)**		**Range of % change**	**Average Community-large Peer-group % change* (95%CI)**	
**Hospital Deaths (HSMR)**	-21 – 22	-4.1 (-7.5 – -0.8)	n/a	-33 – 21	-6.0 (-9.1 – -2.8)	n/a
**Readmission (%)**	-12 – 12	2.1 (0.7 – 3.6)	n/a	-14 – 17	1.6 (-0.3 – 3.4)	n/a

*calculated by summing values of all hospitals within peer-group and dividing by number of hospitals

Table 2 Hospital outcomes on HSMR and Readmission changes over time

Trend outcome	Hospital deaths (HSMR)	Readmission	Teaching hospitals (total n=36) Number, (%)	Community-Large hospitals (total n=45) Number, (%)	Total of all hospitals, number, (%)
Decrease in both HSMR & Readmission	↓	↓	5 (13.9%)	14 (31.1%)	19 (23.5%)
Decrease in HSMR, increase in Readmission	↓	↑	20 (55.6%)	14 (31.1%)	34 (42.0%)
Decrease in HSMR, no change in Readmission	↓	=	1 (2.8%)	2 (4.4%)	3 (3.7%)
Increase in both HSMR & Readmission	↑	↑	7 (19.4%)	8 (17.8%)	15 (18.5%)
Increase in HSMR, decrease in Readmission	↑	↓	2 (5.6%)	1 (2.2%)	3 (3.7%)
Increase in HSMR, no change in Readmission	↑	=	1 (2.8%)	0	1 (1.2%)
No change in both HSMR & Readmission	=	=	0	1 (2.2%)	1 (1.2%)
No change in HSMR, decrease in Readmission	=	↓	0	1 (2.2%)	1 (1.2%)
No change in HSMR, increase in Readmission	=	↑	0	4 (8.9%)	4 (4.9%)

↑=signifies increasing rate; ↓=signifies decreasing rate; = signifies no change

Figure 1 Scatterplot of percent change between 2013-14 – 2017-18 for Readmission and HSMR (by hospital peer-group)

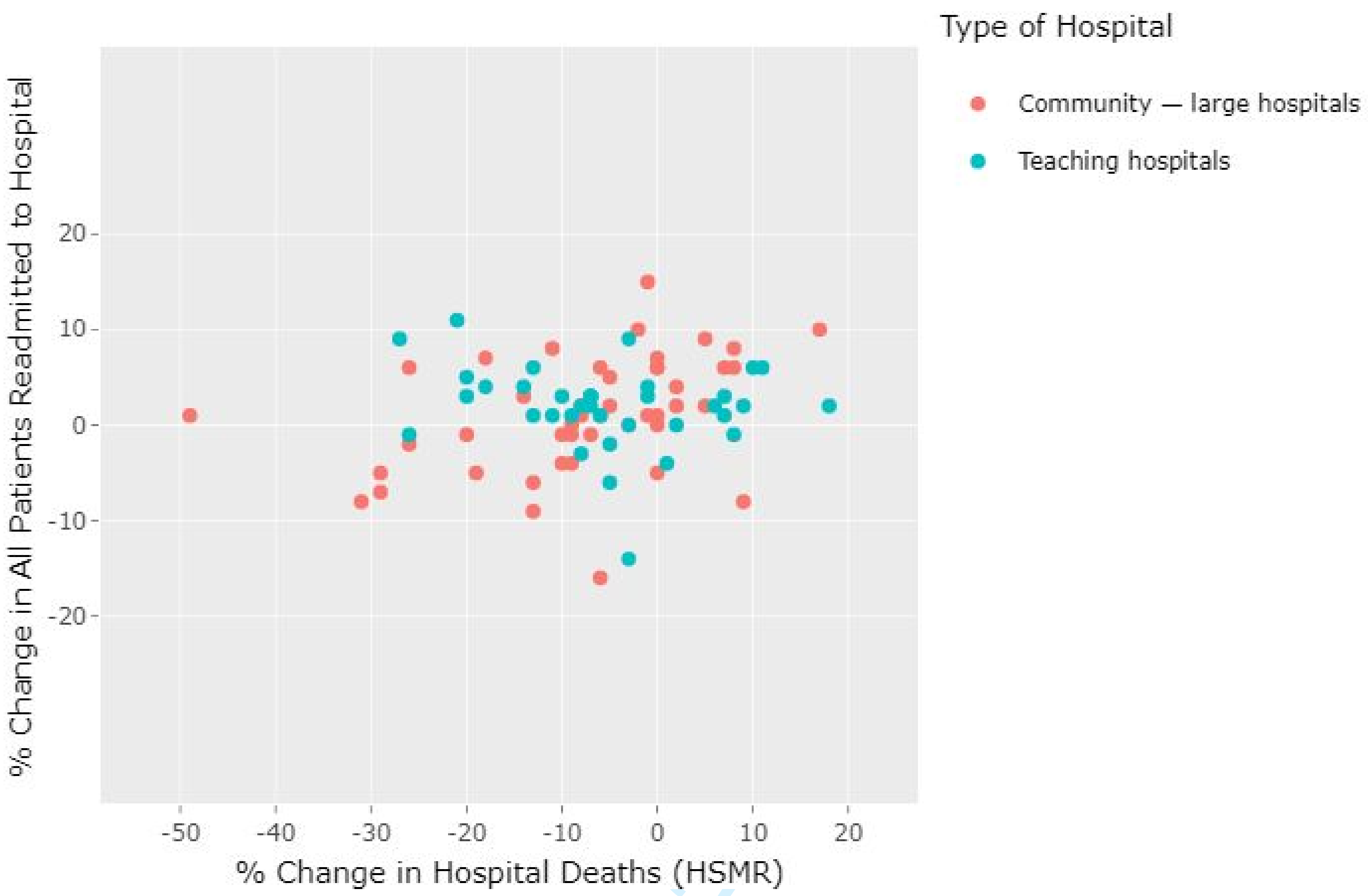

Hospital deaths (HSMR), readmissions and LOS

LOS was moderately positively correlated with hospital deaths (HSMR) in Community-Large hospitals (r=0.4, p <0.01); Teaching hospitals showed no correlation between LOS and hospital deaths (HSMR) or readmissions (see table 3). Readmissions and hospital deaths (HSMR) showed weak to very weak not statistically significant correlations. While Community-Large hospitals showed greater variation in LOS values compared to Teaching hospitals (CoV=24% compared to 16%), their mean peer group LOS values were still lower than Teaching hospitals (6.5 days compared to Teaching hospitals at 7.1) (see table 1). Mean LOS of patients in Community-Large hospitals was 0.6 days shorter, or roughly half a day, compared to Teaching hospitals (6.5 vs. 7.1 days). Figures 2 and 3 illustrate LOS, hospital deaths (HSMR) and readmission values for the 2017-18 data year with LOS delineated in size and shading of bubble plot.

Table 3 Correlations between Hospital Deaths (HSMR), Readmission and LOS (breakdowns by Teaching and Community-Large hospitals)

	Length Of Stay		Hospital deaths (HSMR)	
**Hospital deaths (HSMR)**	Teaching:	**-0.03**		
	Community-Large:	**0.4***		
**Readmission**	Teaching:	**-0.04**	Teaching:	**0.22**
	Community-Large:	**0.04**	Community-Large:	**-0.13**

* p less than .01; ^ p less than .05; Direction of correlation is shown as Blue (positive) and Red (negative), and intensity of cell-colouring reflects strength of correlation. Correlation strength classification: .00-.19 very weak; .20-.39 weak; .40-.59 moderate; .60-.79 strong; .80-1.0 very strong.

For peer review only - http://bmjopen.bmj.com/site/about/guidelines.xhtml

Figure 2 Scatterplot of Teaching hospital values for Hospital Deaths (HSMR), Readmission and LOS (2017-18)

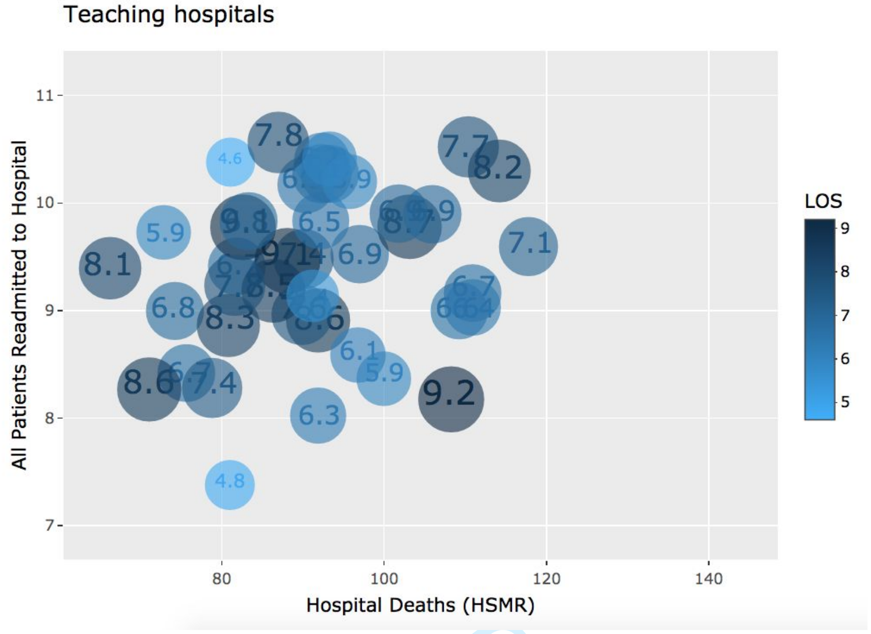

Figure 3 Scatterplot of Community-large hospital values for Hospital Deaths (HSMR), Readmission and LOS (2017-18)

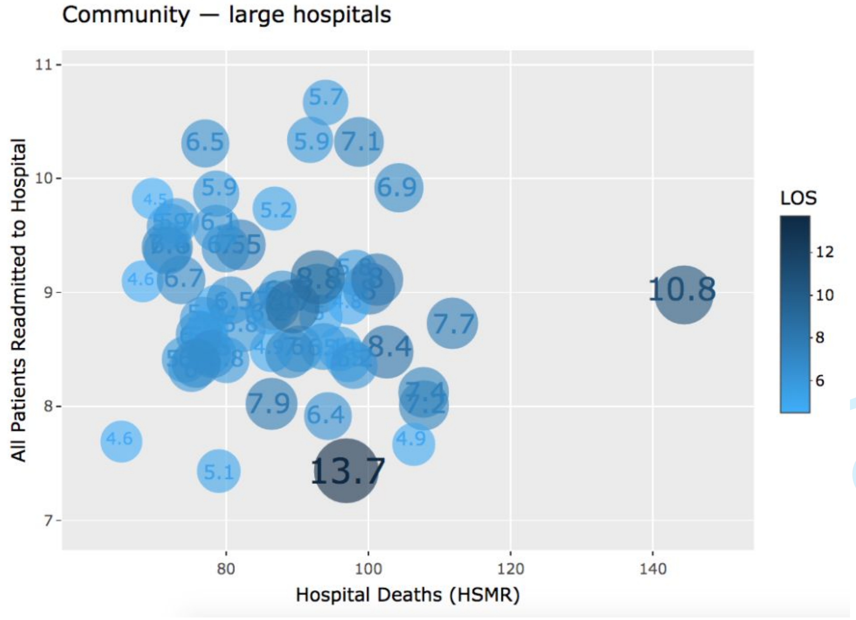

Correlation between hospital characteristics, LOS, Hospital Deaths (HSMR) and Readmission

Table 4 Correlations between hospital characteristics on LOS, HSMR and Readmission

Hospital characteristic	Unit	Length of stay		Hospital deaths (HSMR)		Readmission	
**Number of Acute Care Hospital Stays**	# of days	All:	**-0.04**	All:	**-0.14**	All:	**0.07**
		Teaching:	**0.26**	Teaching:	**-0.3**	Teaching:	**0.07**
		Community-Large:	**-0.36***	Community-Large:	**-0.2**	Community-Large:	**-0.11**
**Number of Acute Care Beds**	# of beds	All:	**0.24***	All:	**-0.01**	All:	**0.03**
		Teaching:	**0.50***	Teaching:	**-0.24**	Teaching:	**-0.03**
		Community-Large:	**-0.02**	Community-Large:	**0.01**	Community-Large:	**-0.17**
**Number of Emergency Department Visits**	# of visits	All:	**-0.13**	All:	**0.03**	All:	**0.04**
		Teaching:	**0.17**	Teaching:	**-0.14**	Teaching:	**0.18**
		Community-Large:	**-0.44***	Community-Large:	**0.13**	Community-Large:	**-0.2**
**Average Acute Care Resource Use Intensity**	Average RIW	All:	**0.68***	All:	**0.39***	All:	**0.15**
		Teaching:	**0.55***	Teaching:	**0**	Teaching:	**0.12**
		Community-Large:	**0.76***	Community-Large:	**0.53***	Community-Large:	**-0.2**
**Total Acute Care Resource Use Intensity**	Total RIWs	All:	**0.13**	All:	**-0.02**	All:	**0.13**
		Teaching:	**0.43***	Teaching:	**-0.25**	Teaching:	**0.11**
		Community-Large:	**-0.16**	Community-Large:	**-0.06**	Community-Large:	**-0.13**
**Hospital Occupancy Rate**	% of occupancy	All:	**0.09**	All:	**-0.14**	All:	**0.01**
		Teaching:	**0.37^**	Teaching:	**-0.28**	Teaching:	**0**
		Community-Large:	**-0.12**	Community-Large:	**-0.1**	Community-Large:	**0.01**
**Patients Admitted Through the Emergency Department**	% of patients	All:	**0.30***	All:	**-0.11**	All:	**0.12**
		Teaching:	**0.47***	Teaching:	**-0.04**	Teaching:	**0.29^**
		Community-Large:	**0.39***	Community-Large:	**-0.1**	Community-Large:	**0.27^**
**Patient Days in Alternate Level of Care**	%	All:	**0.23^**	All:	**-0.01**	All:	**-0.29***
		Teaching:	**0.36^**	Teaching:	**0.02**	Teaching:	**-0.28**
		Community-Large:	**0.24**	Community-Large:	**0.07**	Community-Large:	**-0.13**

* p less than .01; ^ p less than .05; Direction of correlation is shown as Blue (positive) and Red (negative), and intensity of cell-colouring reflects strength of correlation. Correlation strength classification: .00-.19 very weak; .20-.39 weak; .40-.59 moderate; .60-.79 strong; .80-1.0 very strong.

Table 4 shows the correlation between hospital characteristics and LOS, hospital deaths (HSMR) and readmissions. LOS was largely positively correlated (and statistically significant) with the series of eight hospital characteristics. Hospital deaths (HSMR) was largely weak to very weakly negatively correlated. Readmissions were mixed with positive and negative weak to very weak correlations. Correlations between Hospital deaths (HSMR) and readmissions with the eight hospital characteristics were largely not statistically significant (aside from patient days in alternate level of care, patients admitted through the emergency department, and average acute care resource use intensity).

The **Number of Acute Care Hospital Stays** was only statistically significantly correlated with LOS (negatively weakly) in Community-Large hospitals (r= -0.36, p < 0.01). Teaching hospitals had a moderate positive and statistically significant correlation in the **Number of Acute Care Beds** and LOS (r =0.5, p <0.01). The **Number of Emergency Department Visits** and LOS were negatively moderately correlated in Community-Large hospitals (r= -0.44, p <0.01). The **Average Acute Care Resource Use Intensity** was positively strongly correlated with LOS (r= 0.68, p <0.01) when assessing both hospital peer groups. With respect to hospital deaths (HSMR), the average acute care resource use intensity was positively moderately correlated in Community-Large hospitals (r=0.53, p <0.01). **Total Acute Care Resource Use Intensity** was only moderately positively correlated with LOS for Teaching hospitals (r=0.43, p <0.01). **Hospital Occupancy Rate** was only statistically significantly correlated with LOS for Teaching hospitals (r=0.37, p <0.05). With respect to hospital deaths (HSMR), a hospital's occupancy rate is very weak to weakly negatively correlated (and not statistically significant). **Patients Admitted Through the Emergency Department** had a positive weak to moderate correlation with LOS (Teaching hospitals r=0.47, p <0.01; Community-large hospitals r=0.39, p <0.01), and a positive weak correlation with readmissions (Teaching hospitals r=0.29, p <0.05; Community-large hospitals r=0.27, p <0.05). The percentage of **Patient Days in Alternate Level of Care** (a measurement of days patients spend in inpatient acute care, when unneeded, while waiting for discharge to home care or other supports are ready) had a positive weak correlation with LOS in Teaching hospitals (r=0.36, p <0.05), and a weak negative correlation with readmissions for all hospitals combined (r=-0.29, p <0.01).

Supplementary data files include descriptive statistics (mean/percent-change values, CIs, range of values, and number of hospitals) by indicator, facility characteristics, provincial/territorial jurisdiction, and hospital type/size, and correlation matrix histograms.

Discussion

In recent years, there has been growing interest in the association between hospital deaths, readmission and LOS[7]. It is logical to investigate the strength and directionality of correlation between these three components of hospital performance, and with hospital characteristics. There is wide heterogeneity in the available evidence in this research area. Aside from the natural differences across studies that narrow their scope in terms of disease or procedure-specific indicators, limited clinical settings within hospitals, and small denominator groups, even a change in the unit of analysis on the same underlying data, from patient-level data to hospital-level data, can yield disparate results[10].

For peer review only

This secondary analysis of hospital performance and characteristics data aimed to provide a high-level overview of the association between hospital deaths, readmission and LOS across a majority of Teaching and Community-Large hospitals in Canada between 2013-14 and 2017-18. Our earlier research[19] established that, over time, Canadian hospitals have largely improved on in-hospital mortality; readmission rates have been trending upward; and that good or bad performance in one domain of care does not automatically reflect the same performance in other domains. What this present study aimed to add is whether a hospital's improvement or weakening performance over time, in either hospital deaths (HSMR) or readmission, had a positive or negative association on the other; our results showed that 42% of hospitals, the largest proportion across the possible outcomes, in fact decreased hospital deaths (HSMR) while increasing readmission rates. Furthermore, we added LOS to the research question as a proxy of hospital efficiency. Eight hospital characteristics showed trends in strength and directionality of correlation with hospital deaths (HSMR), readmission and LOS.

Strengths and limitations of this study

The main strengths of this study are the quality and extent of data used; all Teaching and Community-Large hospitals across Canada that had publicly-available reported performance results were included in the analysis. The 'all readmission' indicator captures, as the title suggests, all readmission to hospital within 30-days; the hospital deaths (HSMR) indicator captures ~80% of all in-hospital mortality; and the LOS indicator quantifies the mean duration across all hospitalizations. Eight diverse hospital characteristics also provided summary measures that capture numerous aspects of a hospital's performance context. While results for LOS and the eight hospital characteristics were only available for the most-recent year (2017-18), for hospital deaths (HSMR) and readmission indicators, five fiscal year data points were available to measure trend over time differences.

There are limitations in this study with respect to its generalisability beyond Canada; differences in risk-adjustment methodologies, indicator definitions and calculation methods, and hospital type/size definitions, pose challenges to make apples-to-apples comparisons across countries. However, the categorical outcomes of performance simultaneously comparing hospital deaths and readmission, along with the correlation tests of these indicators and hospital characteristics, is available and worthwhile to other settings. Community-Medium and Community-Small hospitals in Canada treat fewer patients, and offer less-complex clinical services. This large group of hospitals (comprising of more than half within the country) are omitted from this study due to an absence of publicly-reported indicator values for hospital deaths. Furthermore, as a result of mergers between disparate hospitals, historic indicator values (i.e., 2013-14 data year) are omitted from the reporting platform. Thus, this inhibits a longitudinal comparison (i.e., performance trend over time). However, current indicator values and hospital characteristics data is available and was included in analyses that only required 2017-18 data year (namely, correlation analyses on hospital characteristics). Some researchers have limited LOS inclusion for those patient cases that are long-LOS or are directly-related to the complementary indicator (i.e., LOS cases only applicable to the indicators of Hospital Deaths or Readmission). This was not feasible in this study as we did not have access to the underlying patient records (just aggregate, hospital-level summaries).

Reflections on the study's findings

Public reporting of performance results poses challenges to hospital administrators and the broader public. Public reporting has become a staple in health systems and hospital performance management. But the practice of public reporting is not without concerns[20]. Tunnel vision and myopia by hospital governance and performance

For peer review only

managers can run the risk of sub-optimisation; the unintended consequences of shifting concentration disproportionately towards areas prioritized for immediate measurement at the expense of other areas of care and broader/long-term organizational goals[21].

Pay for performance schemes are commonplace in hospital governance. A governance model that assesses hospitals through isolated performance measures, runs the risk of unintended consequences in other factors of care and performance not under immediate scrutiny[8]. The results and methods of this study support the notion that quantification of hospital performance should not be done via isolated or single measures at a time, but rather in a more broad and informed mechanism of considering complementary aspects of hospital performance (such as those in the CIHI Hospital Performance Framework: access to services, clinical effectiveness, safety, coordination of care, patient-centeredness, and hospital efficiency)[22]. Furthermore, a poorly conceptualized pay-for-performance scheme may be mal-aligned to take into consideration the correlation (and potential causality) of intensifying efforts to reduce, for example, LOS or hospital mortality, on the increase of readmission rates.

Moreover, government officials charged with hospital governance must take into account inequality across hospital facilities and hospital corporations. Beginning in the 1990s, but increasing rapidly in recent years, there has been a trend of mergers between multiple hospitals and between hospitals and rehabilitation institutes into a singular hospital corporation[23]. These larger hospital corporations in turn have near-exclusive coordination of care between acute-care patients served in hospitals and subsequently their transfer to rehabilitation services. Rural and more-remote hospitals (especially those without paired rehabilitation services) could face higher LOS and occupancy rates, greater number of days and percentage of patients in alternate level of care, and higher resource use intensity. If analysis of these amalgamated hospitals and rehabilitation services proves they perform better than hospitals without direct rehabilitation services, this consideration should also be included in the contextual interpretation (and perhaps risk-adjustment) of hospital performance and governance. Similarly, readmission to hospital may also be a proxy of the strength and availability of primary health care services in the community. Thus, the necessity to consider hospital performance in the broader context of an integrated health service delivery system, a tenet of the accountable care organization movement[24].

Government bodies and professional associations charged with supporting quality improvement initiatives can use the methods and findings of this type of analysis to identify best practices and top-performing hospitals so as to learn from their effective practices. Similarly, hospitals in an unfavourable quadrant (long LOS, and high hospital mortality and readmissions) should receive tailored programs to support their improvement in quality and efficiency of care.

The general public, too, requires consideration when publicly reporting performance results. Efforts in describing indicators in plain language and providing a framework for contextualization can increase the public's assimilation of performance results (especially demographic groups with fewer skills or resources)[25]. CIHI's applies these practices in their online YHS tool, providing their health system performance[26] and hospital performance frameworks[22] as a basis for the curation of performance results, and describing both performance indicators and hospital characteristics in plain language.

The results of this study do not provide a definitive outcome to the debate on the complementarity between LOS, hospital deaths, readmission and hospital characteristics. The underlying pathways and differences between hospitals in functions, and scope of services provided, makes the hospital a complex unit of analyses. The corpus of past studies illustrates the wide heterogeneity of research methods and degree of association outcomes. The embedding of this type of analysis into hospital governance formulation can only better-inform those charged with

For peer review only

policy-making, and administrators of hospitals. Subdividing the research methods of this study, into disease and/or procedure-specific analysis, can help facilitate addressing quality improvement concerns on specific clinical areas; but caution is stressed so as to not unintentionally cause clinicians and hospital administrators to experience tunnel vision.

For peer review only

Conclusions

This study shows that secondary analyses of publicly-reported hospital performance results can reveal meaningful insights into the association among outcome indicators and hospital characteristics. Good or bad hospital performance in one care domain does not necessarily reflect similar performance in other care domains. Thus, caution is warranted in a narrow use of outcome indicators in the design and operationalization of hospital performance measurement and governance models (namely pay-for-performance schemes). Analysis such as this can also inform quality-improvement strategies and targeted efforts to address domains of care experiencing declining performance over time; further granular subdivision of the analyses, for example by hospital peer-groups, can reveal notable differences in performance.

Contributors:

OF initially conceived of the study, reviewed the literature, performed data analysis, interpreted results, and drafted the manuscript. EM assisted in the design of the study, performed and validated data analysis, interpreted results, and reviewed the manuscript. NK assisted with the design of the study, interpreted results, and assisted in the drafting of the manuscript.

Funding:

This research received no specific grant from any funding agency in the public, commercial or not-for-profit sectors.

Competing interests:

None declared. The results and views expressed are those of the authors alone.

Patient consent for publication:

Not required.

Ethics approval:

Not required.

Provenance and peer review:

Not commissioned; externally peer reviewed.

Data availability statement:

All hospital performance and characteristics data used in this study is publicly available via CIHI's Your Health System online tool (http://yourhealthsystem.cihi.ca/).

For peer review only

Supplementary file

Provincial/territorial range of % change difference (2013-14 vs. 2017-18), mean % change (and 95% Confidence Intervals), combined Teaching and Community-Large hospitals

Province/territory	Indicator	Range of % change (2013-14 vs. 2017-18)	Mean % change (95% CI)
Alberta	All Patients Readmitted to Hospital	-6 - 17	3.1 (-0.7 – 6.9)
	Hospital Deaths (HSMR)	-21 - 22	-0.7 (-9.3 – 7.9)
British Columbia	All Patients Readmitted to Hospital	-12 - 12	1.9 (-1.2 – 5)
	Hospital Deaths (HSMR)	-33 - 11	-6.5 (-11.4 – -1.6)
Manitoba	All Patients Readmitted to Hospital	3 - 10	6.7 (-2.1 – 15.4)
	Hospital Deaths (HSMR)	-13 - 12	-1.3 (-32.6 – 29.9)
New Brunswick	All Patients Readmitted to Hospital	-8 - 2	-3.2 (-11.2 – 4.7)
	Hospital Deaths (HSMR)	-11 - 10	-2.5 (-16.8 – 11.8)
Newfoundland and Labrador	All Patients Readmitted to Hospital	1 - 10	5.5 (-51.7 – 62.7)
	Hospital Deaths (HSMR)	-6 - 6	0.0 (-76.2 – 76.2)
Nova Scotia	All Patients Readmitted to Hospital	-4 - 11	3.5 (-91.8 – 98.8)
	Hospital Deaths (HSMR)	1 - 21	11.0 (-116.1 – 138.1)
Ontario	All Patients Readmitted to Hospital	-14 - 9	0.9 (-1 – 2.8)
	Hospital Deaths (HSMR)	-24 - 8	-5.8 (-9.2 – -2.5)
Prince Edward Island*	All Patients Readmitted to Hospital	-5 – -5	N/A
	Hospital Deaths (HSMR)	-22 – -22	N/A
Quebec	All Patients Readmitted to Hospital	1 – 9	4.8 (1.8 – 7.8)
	Hospital Deaths (HSMR)	-21 – -1	-12.0 (-19.5 – -4.5)
Saskatchewan	All Patients Readmitted to Hospital	-2 – 3	0.8 (-2.5 – 4)
	Hospital Deaths (HSMR)	-11 – -5	-7.8 (-11.7 – -3.8)

*Only one hospital value.

For peer review only

Subset of hospitals (n=81), with both Readmission and Hospital Deaths (HSMR) values, used in performance trends over time analysis

Provincial/territorial jurisdiction	Community — large hospitals	Teaching hospitals	Jurisdiction total
Alberta	4	7	11
British Columbia	11	6	17
Manitoba	1	2	3
New Brunswick	3	1	4
Newfoundland and Labrador	1	1	2
Nova Scotia	1	1	2
Ontario	21	10	31
Prince Edward Island	1	0	1
Quebec	2	4	6
Saskatchewan	0	4	4
**Total**	**45**	**36**	**81**

Facility characteristic averages by hospital peer-groups

Facility characteristic	Unit	Mean value, (n of hospitals)	
		Teaching hospitals	Community – Large hospitals
**Number of Acute Care Hospital Stays**	# of days	27,322 (n=53)	20,421 (n=66)
**Number of Acute Care Beds**	# of beds	474 (n=53)	328 (n=66)
**Number of Emergency Department Visits**	# of visits	83,441 (n=40)	86,962 (n=43)
**Average Acute Care Resource Use Intensity**	average RIW	1.6 (n=53)	1.2 (n=66)
**Total Acute Care Resource Use Intensity**	total RIWs	43,295 (n=53)	25,057 (n=66)
**Hospital Occupancy Rate**	% of occupancy	88.9 (n=44)	89.9 (n=61)
**Patients Admitted Through the Emergency Department (%)**	% of patients	44.4 (n=53)	54.4 (n=66)
**Patient Days in Alternate Level of Care (Percentage)**	%	11.4 (n=43)	15.4 (n=53)

For peer review only

<u>Correlation matrix (histogram) of both Teaching and Community-Large hospitals</u>

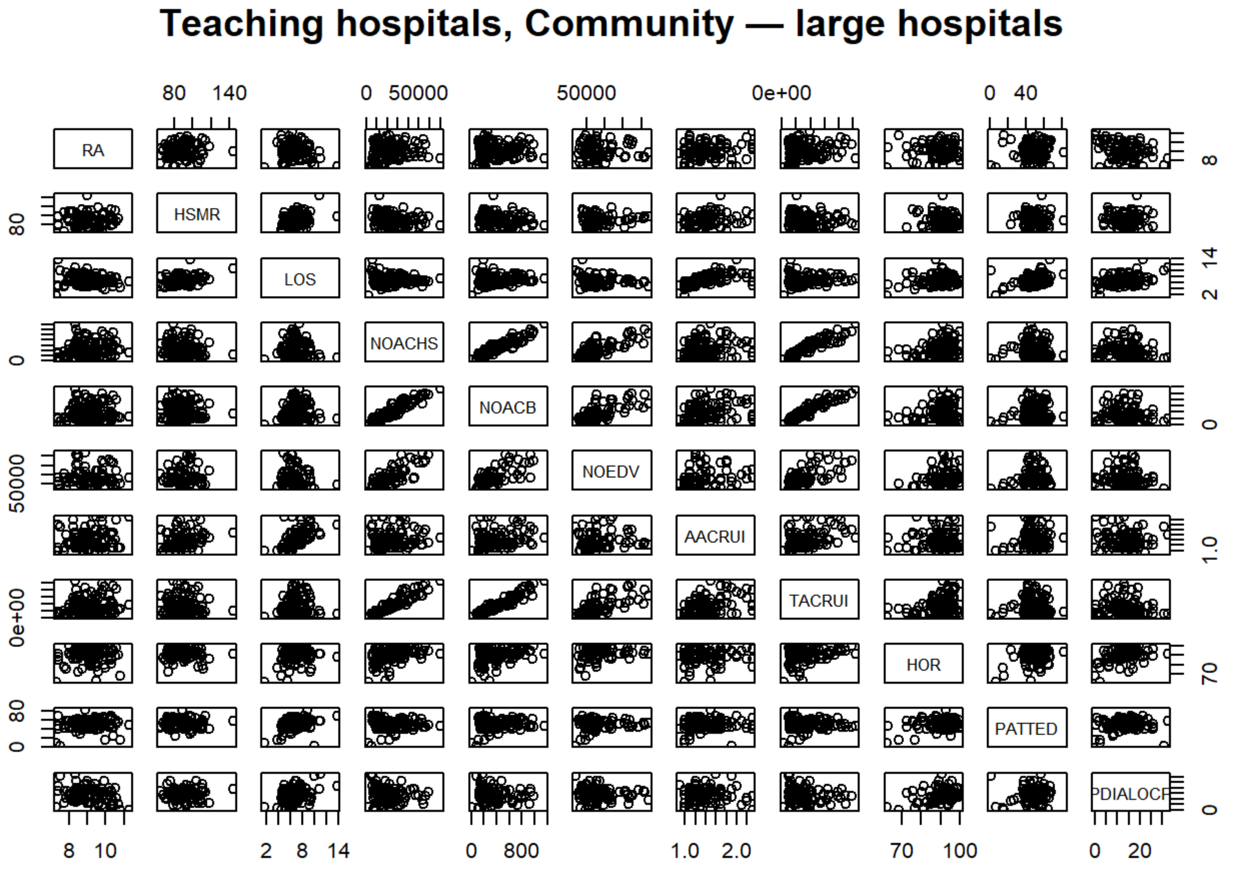

Indicator acronyms: All Patients Readmitted to Hospital (RA); Hospital Deaths (HSMR); Average Length of Stay (LOS); Number of Acute Care Hospital Stays (NOACHS); Number of Acute Care Beds (NOACB); Number of Emergency Department Visits (NOEDV); Average Acute Care Resource Use Intensity (AACRUI); Total Acute Care Resource Use Intensity (TACRUI); Hospital Occupancy Rate (HOR); Patients Admitted Through the Emergency Department (%) (PATTED); Patient Days in Alternate Level of Care (Percentage) (PDIALOCP).

Correlation matrix (histogram) of Teaching hospitals

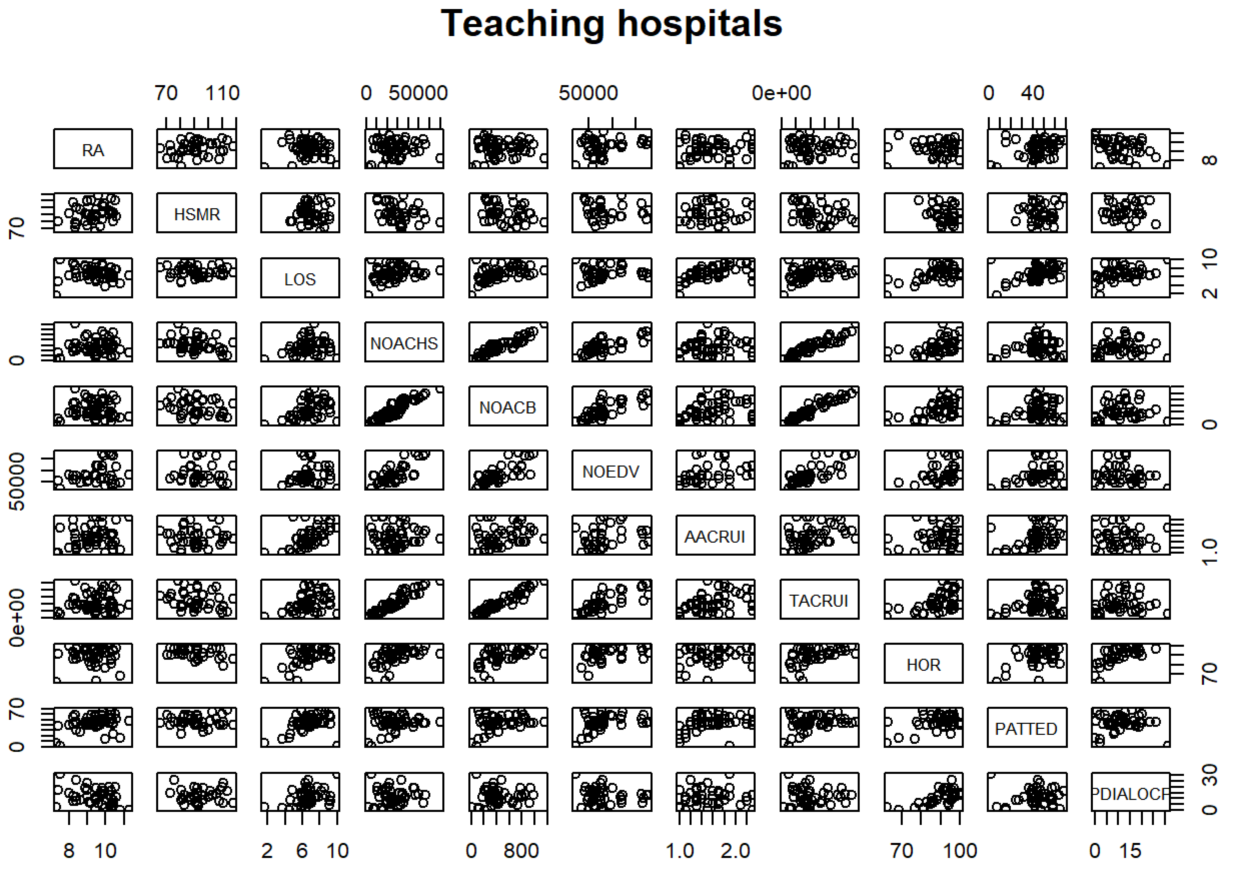

Indicator acronyms: All Patients Readmitted to Hospital (RA); Hospital Deaths (HSMR); Average Length of Stay (LOS); Number of Acute Care Hospital Stays (NOACHS); Number of Acute Care Beds (NOACB); Number of Emergency Department Visits (NOEDV); Average Acute Care Resource Use Intensity (AACRUI); Total Acute Care Resource Use Intensity (TACRUI); Hospital Occupancy Rate (HOR); Patients Admitted Through the Emergency Department (%) (PATTED); Patient Days in Alternate Level of Care (Percentage) (PDIALOCP).

Correlation matrix (histogram) of Community-Large hospitals

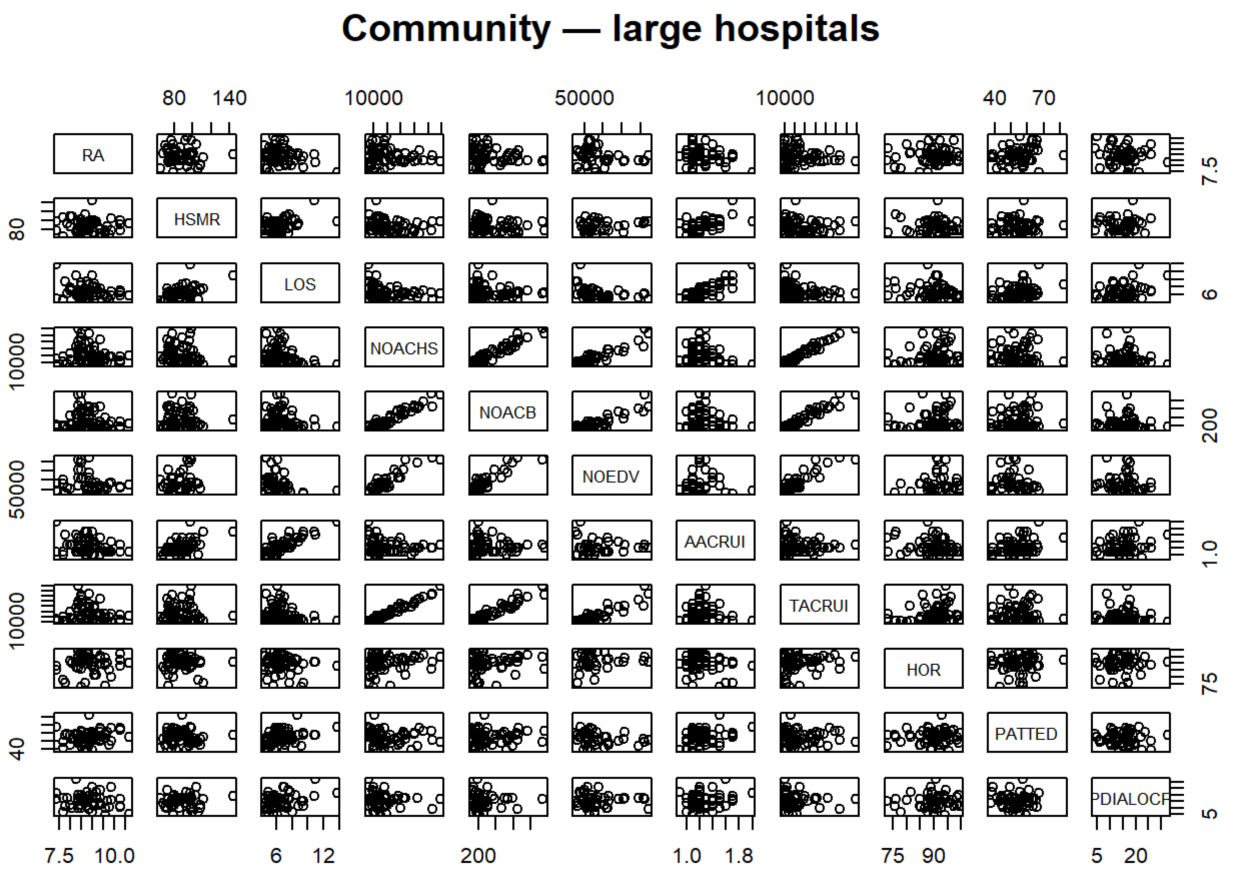

Indicator acronyms: All Patients Readmitted to Hospital (RA); Hospital Deaths (HSMR); Average Length of Stay (LOS); Number of Acute Care Hospital Stays (NOACHS); Number of Acute Care Beds (NOACB); Number of Emergency Department Visits (NOEDV); Average Acute Care Resource Use Intensity (AACRUI); Total Acute Care Resource Use Intensity (TACRUI); Hospital Occupancy Rate (HOR); Patients Admitted Through the Emergency Department (%) (PATTED); Patient Days in Alternate Level of Care (Percentage) (PDIALOCP).

References

[1] Oliver Groene, Jutta K. H. Skau, Anne Frølich; An international review of projects on hospital performance assessment, International Journal for Quality in Health Care, Volume 20, Issue 3, 1 June 2008, Pages 162–171,

[2] Helene Eckhardt, Peter Smith, Wilm Quentin. (2019). Pay for Quality: using financial incentives to improve quality of care. In R. Busse, N. Klazinga, D. Panteli, W. Quentin (Eds). Improving healthcare quality in Europe. Characteristics, effectiveness and implementation of different strategies. WHO Regional Office Europe. Copenhagen 2019, pp. 233-264.

[3] Litvak, Eugene & Bisognano, Maureen. (2011). More Patients, Less Payment: Increasing Hospital Efficiency In The Aftermath Of Health Reform. Health affairs (Project Hope). 30. 76-80. 10.1377/hlthaff.2010.1114.

[4] Shahian, D. M., Iezzoni, L. I., Meyer, G. S., Kirle, L., & Normand, S.-L. T. (2012). Hospital-wide Mortality as a Quality Metric: Conceptual and Methodological Challenges. American Journal of Medical Quality, 27(2), 112–123.

[5] Fischer C, Lingsma HF, Marang-van de Mheen PJ, Kringos DS, Klazinga NS, Steyerberg EW. Is the readmission rate a valid quality indicator? A review of the evidence [published correction appears in PLoS One. 2015;10(2):e0118968]. PLoS One. 2014;9(11).

[6] Cylus, Jonathan, Papanicolas, Irene and Smith, Peter C. (2016) Conclusions. In: Cylus, Jonathan, Papanicolas, Irene and Smith, Peter C., (eds.) Health system efficiency: How to make measurement matter for policy and management. Health Policy Series. European Observatory on Health Systems and Policies, Brussels, Belgium, pp. 225-241.

[7] Lingsma HF, Bottle A, Middleton S, et al. Evaluation of hospital outcomes: the relation between length-of-stay, readmission, and mortality in a large international administrative database. BMC Health Serv Res. 2018;18(1):116.

[8] Kaboli PJ, Go JT, Hockenberry J, et al. Associations Between Reduced Hospital Length of Stay and 30-Day Readmission Rate and Mortality: 14-Year Experience in 129 Veterans Affairs Hospitals. Ann Intern Med. 2012;157:837–845.

[9] Dharmarajan K, Wang Y, Lin Z, et al. Association of Changing Hospital Readmission Rates With Mortality Rates After Hospital Discharge. JAMA. 2017;318(3):270–278.

[10] Hofstede SN, van Bodegom-Vos L, Kringos DS, et al. Mortality, readmission and length of stay have different relationships using hospital-level versus patient-level data: an example of the ecological fallacy affecting hospital performance indicators. BMJ Quality & Safety 2018;27:474-483.

For peer review only

[11] Auger, K. A., Teufel, R. J., Harris, J. M., Gay, J. C., Del Beccaro, M. A., Neuman, M. I., ... Shah, S. S. (2017). Children's hospital characteristics and readmission metrics. *Pediatrics*, *139*(2).

[12] Paterson JM, Williams JI, Kreder HJ, et al. Provider volumes and early outcomes of primary total joint replacement in Ontario. Can J Surg. 2010;53(3):175–183.

[13] Canadian Institute for Health Information. Your Health System – In Depth [internet]. Ottawa: CIHI, 2019 https://yourhealthsystem.ca (accessed 18 November 2019).

[14] Canadian Institute for Health Information. Indicator Library [internet]. Ottawa: CIHI; 2019 https://www.cihi.ca/en/indicator-library (accessed 18 November 2019).

[15] Canadian Institute for Health Information. Your Health System: In Depth. Technical Notes for Contextual Measures (October 2019). Ottawa: CIHI; 2019.

[16] Canadian Institute for Health Information. Indicator Library: Peer Group Methodology. Ottawa: CIHI; 2016.

[17] Canadian Institute for Health Information. HSMR: Frequently asked questions [internet]. Ottawa: CIHI; 2020 https://www.cihi.ca/en/hospital-standardized-mortality-ratio-hsmr-frequently-asked-questions#_faq22 (accessed 13 April 2020).

[18] BMJ. 11. Correlation and regression [internet]. London; BMJ; 2020 https://www.bmj.com/about-bmj/resources-readers/publications/statistics-square-one/11-correlation-and-regression (accessed 13 April 2020).

[19] Fekri O, Manukyan E, Klazinga N. Appropriateness, effectiveness, and safety of care delivered in Canadian hospitals: a longitudinal assessment on the utility of publicly reported performance trend data between 2012-2013 and 2016-2017. BMJ Open 2020; **O**:e035447. Doi:10.1136/bmjopen-2019-035447

[20] Martin M, Davies H. Public release of information on quality of care: how are health services and the public expected to respond?, J Health Serv Res Policy, 2001, vol. 6 (pg. 158-62)

[21] Smith, P. (1995) On the Unintended Consequences of Publishing Performance Data in the Public Sector. International Journal of Public Administration, 18, 277-310.

[22] Canadian Institute for Health Information. A Performance Measurement Framework for Canadian Hospitals, 2013. Ottawa: CIHI; 2013.

For peer review only

[23] Howlett, K. Health-care providers unite in bid to improve care, cut costs. 2011; https://www.theglobeandmail.com/news/toronto/health-care-providers-unite-in-bid-to-improve-care-cut-costs/article575685/ (accessed 23 December 2019).

[24] Huynh TM, Baker GR, Bierman A, et al. Exploring accountable care in Canada: integrating financial and quality incentives for physicians and hospitals. Ottawa: Canadian Foundation for Healthcare Improvement; 2014.

[25] Hibbard, J. H., Greene, J., & Daniel, D. (2010). What Is Quality Anyway? Performance Reports That Clearly Communicate to Consumers the Meaning of Quality of Care. Medical Care Research and Review, 67(3), 275–293.

[26] Canadian Institute for Health Information. A performance measurement framework for the Canadian health system. Ottawa: Canadian Institute for Health Information; 2013.

For peer review only

Supplementary file

Provincial/territorial range of % change difference (2013-14 vs. 2017-18), mean % change (and 95% Confidence Intervals), combined Teaching and Community-Large hospitals

Province/territory	Indicator	Range of % change (2013-14 vs. 2017-18)	Mean % change (95% CI)
Alberta	All Patients Readmitted to Hospital	-6 - 17	3.1 (-0.7 – 6.9)
	Hospital Deaths (HSMR)	-21 - 22	-0.7 (-9.3 – 7.9)
British Columbia	All Patients Readmitted to Hospital	-12 - 12	1.9 (-1.2 – 5)
	Hospital Deaths (HSMR)	-33 - 11	-6.5 (-11.4 – -1.6)
Manitoba	All Patients Readmitted to Hospital	3 - 10	6.7 (-2.1 – 15.4)
	Hospital Deaths (HSMR)	-13 - 12	-1.3 (-32.6 – 29.9)
New Brunswick	All Patients Readmitted to Hospital	-8 - 2	-3.2 (-11.2 – 4.7)
	Hospital Deaths (HSMR)	-11 - 10	-2.5 (-16.8 – 11.8)
Newfoundland and Labrador	All Patients Readmitted to Hospital	1 - 10	5.5 (-51.7 – 62.7)
	Hospital Deaths (HSMR)	-6 - 6	0.0 (-76.2 – 76.2)
Nova Scotia	All Patients Readmitted to Hospital	-4 - 11	3.5 (-91.8 – 98.8)
	Hospital Deaths (HSMR)	1 - 21	11.0 (-116.1 – 138.1)
Ontario	All Patients Readmitted to Hospital	-14 - 9	0.9 (-1 – 2.8)
	Hospital Deaths (HSMR)	-24 - 8	-5.8 (-9.2 – -2.5)
Prince Edward Island*	All Patients Readmitted to Hospital	-5 – -5	N/A
	Hospital Deaths (HSMR)	-22 – -22	N/A
Quebec	All Patients Readmitted to Hospital	1 – 9	4.8 (1.8 – 7.8)
	Hospital Deaths (HSMR)	-21 – -1	-12.0 (-19.5 – -4.5)
Saskatchewan	All Patients Readmitted to Hospital	-2 – 3	0.8 (-2.5 – 4)
	Hospital Deaths (HSMR)	-11 – -5	-7.8 (-11.7 – -3.8)

*Only one hospital value.

For peer review only

Subset of hospitals (n=81), with both Readmission and Hospital Deaths (HSMR) values, used in performance trends over time analysis

Provincial/territorial jurisdiction	Community — large hospitals	Teaching hospitals	Jurisdiction total
Alberta	4	7	11
British Columbia	11	6	17
Manitoba	1	2	3
New Brunswick	3	1	4
Newfoundland and Labrador	1	1	2
Nova Scotia	1	1	2
Ontario	21	10	31
Prince Edward Island	1	0	1
Quebec	2	4	6
Saskatchewan	0	4	4
**Total**	**45**	**36**	**81**

Facility characteristic averages by hospital peer-groups

Facility characteristic	Unit	Mean value, (n of hospitals)	
		Teaching hospitals	Community – Large hospitals
**Number of Acute Care Hospital Stays**	# of days	27,322 (n=53)	20,421 (n=66)
**Number of Acute Care Beds**	# of beds	474 (n=53)	328 (n=66)
**Number of Emergency Department Visits**	# of visits	83,441 (n=40)	86,962 (n=43)
**Average Acute Care Resource Use Intensity**	average RIW	1.6 (n=53)	1.2 (n=66)
**Total Acute Care Resource Use Intensity**	total RIWs	43,295 (n=53)	25,057 (n=66)
**Hospital Occupancy Rate**	% of occupancy	88.9 (n=44)	89.9 (n=61)
**Patients Admitted Through the Emergency Department (%)**	% of patients	44.4 (n=53)	54.4 (n=66)
**Patient Days in Alternate Level of Care (Percentage)**	%	11.4 (n=43)	15.4 (n=53)

For peer review only

Correlation matrix (histogram) of both Teaching and Community-Large hospitals

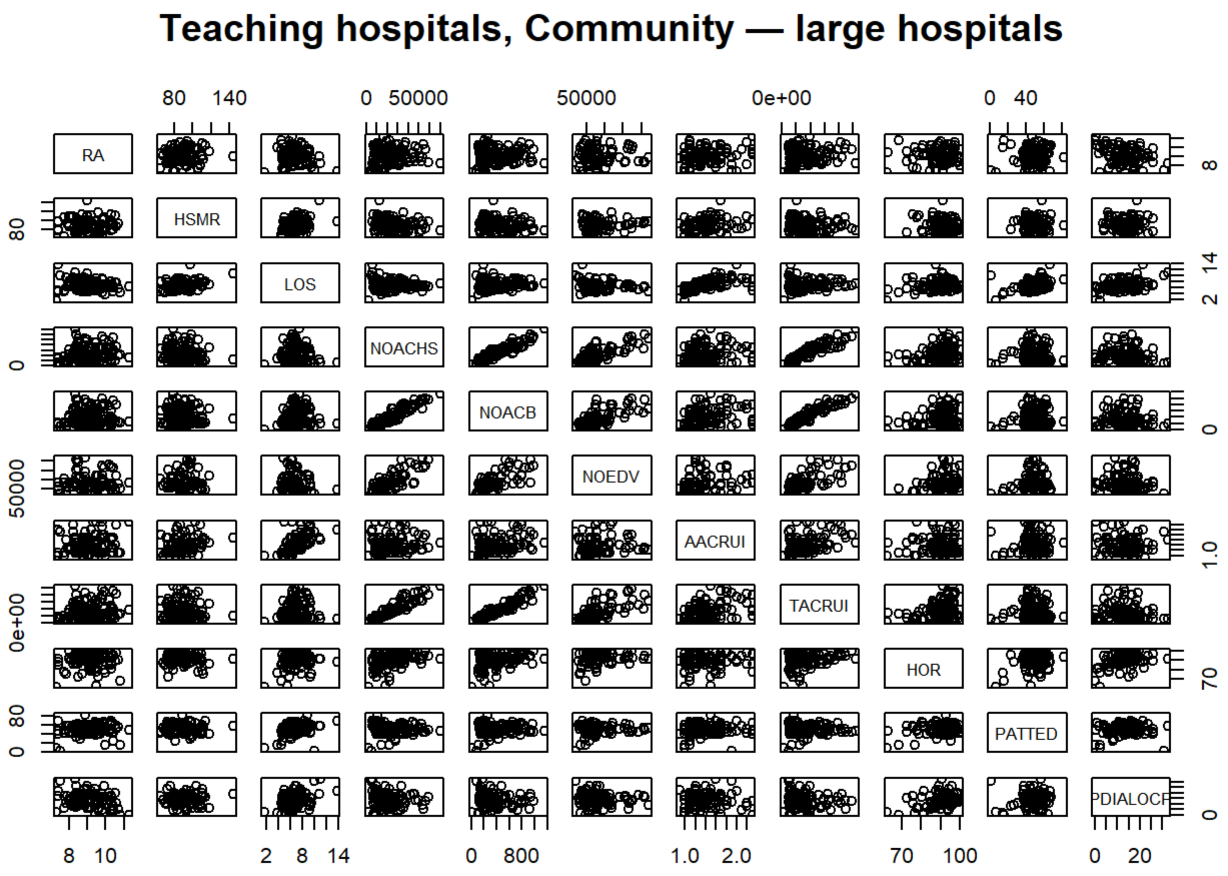

Indicator acronyms: All Patients Readmitted to Hospital (RA); Hospital Deaths (HSMR); Average Length of Stay (LOS); Number of Acute Care Hospital Stays (NOACHS); Number of Acute Care Beds (NOACB); Number of Emergency Department Visits (NOEDV); Average Acute Care Resource Use Intensity (AACRUI); Total Acute Care Resource Use Intensity (TACRUI); Hospital Occupancy Rate (HOR); Patients Admitted Through the Emergency Department (%) (PATTED); Patient Days in Alternate Level of Care (Percentage) (PDIALOCP).

Correlation matrix (histogram) of Teaching hospitals

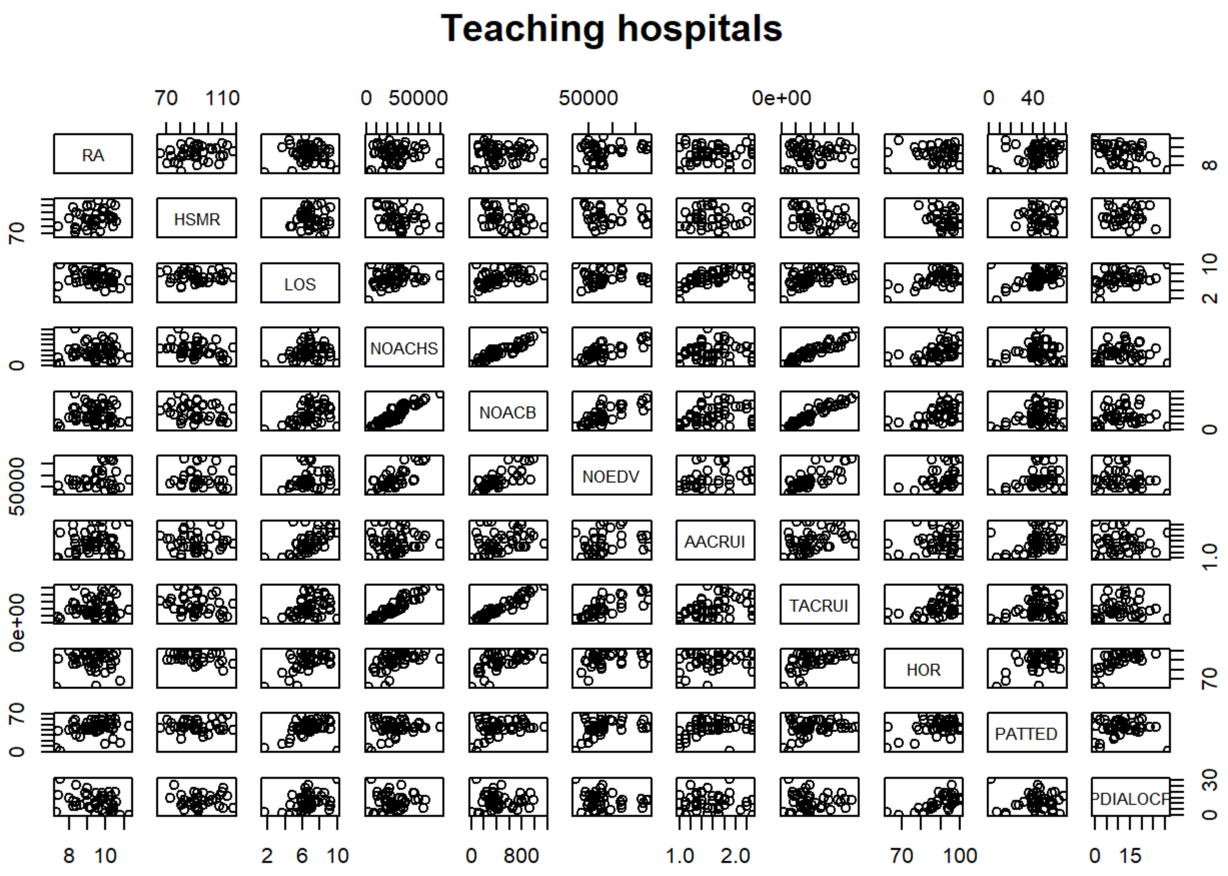

Indicator acronyms: All Patients Readmitted to Hospital (RA); Hospital Deaths (HSMR); Average Length of Stay (LOS); Number of Acute Care Hospital Stays (NOACHS); Number of Acute Care Beds (NOACB); Number of Emergency Department Visits (NOEDV); Average Acute Care Resource Use Intensity (AACRUI); Total Acute Care Resource Use Intensity (TACRUI); Hospital Occupancy Rate (HOR); Patients Admitted Through the Emergency Department (%) (PATTED); Patient Days in Alternate Level of Care (Percentage) (PDIALOCP).

Correlation matrix (histogram) of Community-Large hospitals

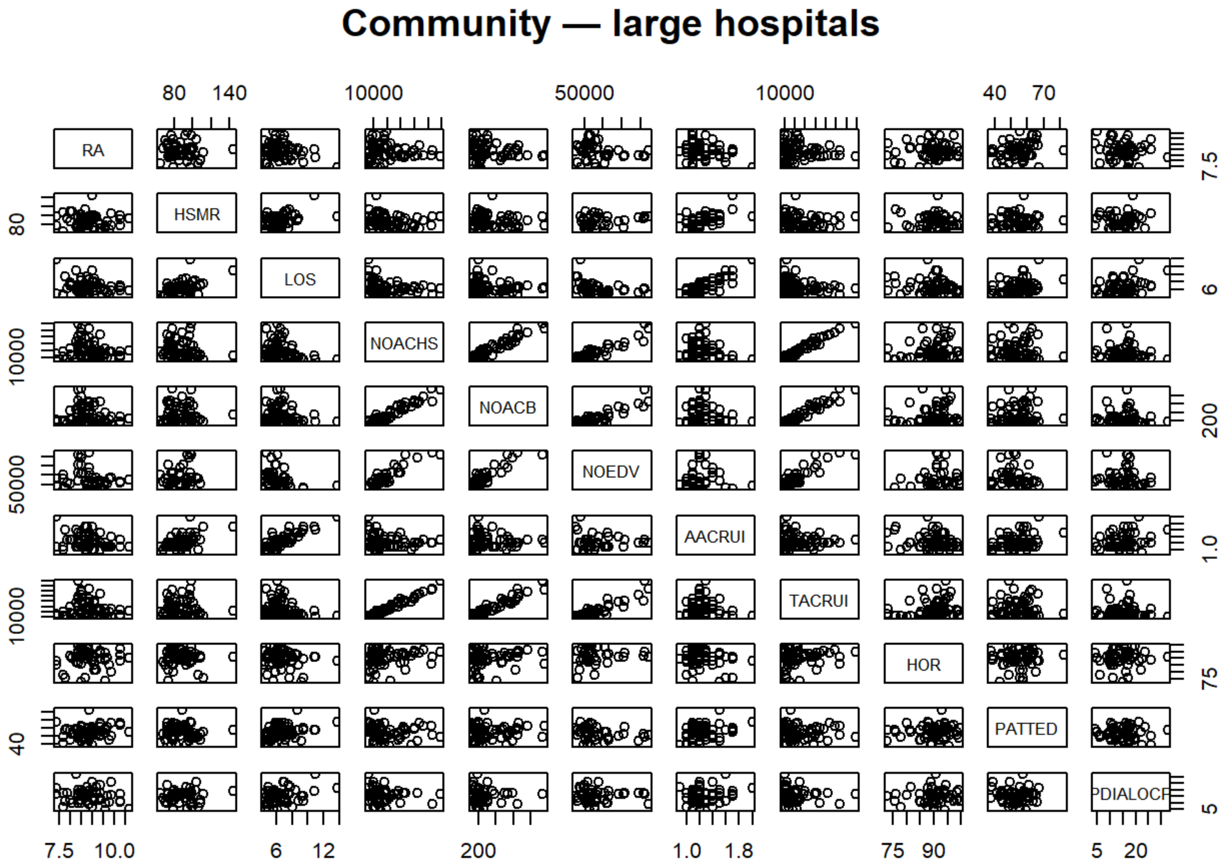

Indicator acronyms: All Patients Readmitted to Hospital (RA); Hospital Deaths (HSMR); Average Length of Stay (LOS); Number of Acute Care Hospital Stays (NOACHS); Number of Acute Care Beds (NOACB); Number of Emergency Department Visits (NOEDV); Average Acute Care Resource Use Intensity (AACRUI); Total Acute Care Resource Use Intensity (TACRUI); Hospital Occupancy Rate (HOR); Patients Admitted Through the Emergency Department (%) (PATTED); Patient Days in Alternate Level of Care (Percentage) (PDIALOCP).

The RECORD statement – checklist of items, extended from the STROBE statement, that should be reported in observational studies using routinely collected health data.

	Item No.	STROBE items	Location in manuscript where items are reported	RECORD items	Location in manuscript where items are reported
**Title and abstract**					
	1	(a) Indicate the study's design with a commonly used term in the title or the abstract (b) Provide in the abstract an informative and balanced summary of what was done and what was found		RECORD 1.1: The type of data used should be specified in the title or abstract. When possible, the name of the databases used should be included.<br><br>RECORD 1.2: If applicable, the geographic region and timeframe within which the study took place should be reported in the title or abstract.<br><br>RECORD 1.3: If linkage between databases was conducted for the study, this should be clearly stated in the title or abstract.	1.1 Noted in title and abstract.<br><br>1.2 Noted in title and abstract.<br><br>1.3. Not applicable as no linkages were performed.
**Introduction**					
Background rationale	2	Explain the scientific background and rationale for the investigation being reported			Introduction paragraphs 1-4
Objectives	3	State specific objectives, including any prespecified hypotheses			Introduction paragraph 4
**Methods**					
Study Design	4	Present key elements of study design early in the paper			Methods section
Setting	5	Describe the setting, locations, and relevant dates, including periods of recruitment, exposure, follow-up, and data collection			Methods paragraphs 1-11
Participants	6	*(a) Cohort study* - Give the eligibility criteria, and the sources and methods of selection of participants. Describe methods of follow-up<br>*Case-control study* - Give the eligibility criteria, and the sources and methods of case ascertainment and control selection. Give the rationale for the choice of cases and controls<br>*Cross-sectional study* - Give the eligibility criteria, and the sources and methods of selection of participants<br><br>*(b) Cohort study* - For matched studies, give matching criteria and number of exposed and unexposed<br>*Case-control study* - For matched studies, give matching criteria and the number of controls per case		RECORD 6.1: The methods of study population selection (such as codes or algorithms used to identify subjects) should be listed in detail. If this is not possible, an explanation should be provided.<br><br>RECORD 6.2: Any validation studies of the codes or algorithms used to select the population should be referenced. If validation was conducted for this study and not published elsewhere, detailed methods and results should be provided.<br><br>RECORD 6.3: If the study involved linkage of databases, consider use of a flow diagram or other graphical display to demonstrate the data linkage process, including the number of individuals with linked data at each stage.	6.1 N/A<br><br>6.2 N/A<br><br>6.3 N/A
Variables	7	Clearly define all outcomes, exposures, predictors, potential confounders, and effect modifiers. Give diagnostic criteria, if applicable.		RECORD 7.1: A complete list of codes and algorithms used to classify exposures, outcomes, confounders, and effect modifiers should be provided. If these cannot be reported, an explanation should be provided.	7.1 Outcomes and variables described in the Methods section paragraphs 1-7, 9, 10
Data sources/ measurement	8	For each variable of interest, give sources of data and details of methods of assessment (measurement).<br>Describe comparability of assessment methods if there is more than one group			Data source described in Methods paragraph 1

For peer review only

Bias	9	Describe any efforts to address potential sources of bias			Bias of available data described in Methods paragraph 5
Study size	10	Explain how the study size was arrived at			Methods paragraph 5
Quantitative variables	11	Explain how quantitative variables were handled in the analyses. If applicable, describe which groupings were chosen, and why			Groupings described in Methods paragraph 6<br><br>Quantitative variables described in Methods paragraphs 7-10
Statistical methods	12	(a) Describe all statistical methods, including those used to control for confounding<br>(b) Describe any methods used to examine subgroups and interactions<br>(c) Explain how missing data were addressed<br>(d) *Cohort study* - If applicable, explain how loss to follow-up was addressed<br>*Case-control study* - If applicable, explain how matching of cases and controls was addressed<br>*Cross-sectional study* - If applicable, describe analytical methods taking account of sampling strategy<br>(e) Describe any sensitivity analyses			Methods paragraphs 6-10
Data access and cleaning methods		..		RECORD 12.1: Authors should describe the extent to which the investigators had access to the database population used to create the study population.<br><br>RECORD 12.2: Authors should provide information on the data cleaning methods used in the study.	12.1 Noted in methods section that data is publicly available for use. Also described in Data Availability Statement at conclusion of manuscript.<br><br>12.2 No data cleaning methods were used in the study.
Linkage		..		RECORD 12.3: State whether the study included person-level, institutional-level, or other data linkage across two or more databases. The methods of linkage and methods of linkage quality evaluation should be provided.	12.3 No data linkage was performed.
**Results**					
Participants	13	(a) Report the numbers of individuals at each stage of the study (*e.g.*, numbers potentially eligible, examined for eligibility, confirmed eligible, included in the study, completing follow-up, and analysed)<br>(b) Give reasons for non-participation at each stage.<br>(c) Consider use of a flow diagram		RECORD 13.1: Describe in detail the selection of the persons included in the study (*i.e.,* study population selection) including filtering based on data quality, data availability and linkage. The selection of included persons can be described in the text and/or by means of the study flow diagram.	13.1 No person-level data was used in the study. Number of hospitals included in study described in Methods paragraph 6, and Results section Table 1, and supplementary file
Descriptive data	14	(a) Give characteristics of study participants (*e.g.*, demographic, clinical, social) and information on exposures and potential confounders<br>(b) Indicate the number of participants with missing data for each variable of interest<br>(c) *Cohort study* - summarise follow-up time (*e.g.*, average and total amount)			Descriptive information on hospitals are stated in Methods section, and in Table 1 of Results section.

For peer review only

Outcome data	15	*Cohort study* - Report numbers of outcome events or summary measures over time<br>*Case-control study* - Report numbers in each exposure category, or summary measures of exposure<br>*Cross-sectional study* - Report numbers of outcome events or summary measures			Reported in Table 2 of Results section.
Main results	16	(a) Give unadjusted estimates and, if applicable, confounder-adjusted estimates and their precision (e.g., 95% confidence interval). Make clear which confounders were adjusted for and why they were included<br>(b) Report category boundaries when continuous variables were categorized<br>(c) If relevant, consider translating estimates of relative risk into absolute risk for a meaningful time period			The Results section contains three main headings (corresponding to research questions 1,2,3, with the 4th addressed concurrently).
Other analyses	17	Report other analyses done—e.g., analyses of subgroups and interactions, and sensitivity analyses			Subgroup analyses by hospital type/size are described throughout Results section, notably tables 1,2,3 & figures 1,2,3.<br><br>Jurisdictional and hospital type/size breakdowns provided in supplementary file.
**Discussion**					
Key results	18	Summarise key results with reference to study objectives			Discussion paragraph 2<br>Conclusion paragraph 1
Limitations	19	Discuss limitations of the study, taking into account sources of potential bias or imprecision. Discuss both direction and magnitude of any potential bias		RECORD 19.1: Discuss the implications of using data that were not created or collected to answer the specific research question(s). Include discussion of misclassification bias, unmeasured confounding, missing data, and changing eligibility over time, as they pertain to the study being reported.	19.1 Noted under paragraph 2 of Strengths & Limitations section of Discussion section.
Interpretation	20	Give a cautious overall interpretation of results considering objectives, limitations, multiplicity of analyses, results from similar studies, and other relevant evidence			Paragraph 10 of Discussion section.
Generalisability	21	Discuss the generalisability (external validity) of the study results			Discussion paragraph 4
**Other Information**					
Funding	22	Give the source of funding and the role of the funders for the present study and, if applicable, for the original study on which the present article is based			Funding statement
Accessibility of protocol, raw data, and programming code		..		RECORD 22.1: Authors should provide information on how to access any supplemental information such as the study protocol, raw data, or programming code.	22.1 Noted in Data Availability Statement, and cited in Methods section.

For peer review only

*Reference: Benchimol EI, Smeeth L, Guttmann A, Harron K, Moher D, Petersen I, Sørensen HT, von Elm E, Langan SM, the RECORD Working Committee. The REporting of studies Conducted using Observational Routinely-collected health Data (RECORD) Statement. *PLoS Medicine* 2015; in press.

*Checklist is protected under Creative Commons Attribution (CC BY) license.

For peer review only

BMJ Open

Associations between Hospital Deaths (HSMR), Readmission and Length of Stay (LOS): a longitudinal assessment of performance results and facility characteristics of teaching and large-sized hospitals in Canada between 2013-14 to 2017-18

Journal:	*BMJ Open*
Manuscript ID	bmjopen-2020-041648.R1
Article Type:	Original research
Date Submitted by the Author:	27-Oct-2020

Complete List of Authors:	Fekri, Omid; University of Amsterdam, Academic Medical Center<br>Manukyan, Edgar; University of Amsterdam, Academic Medical Center<br>Klazinga, Niek; University of Amsterdam, Academic Medical Center

**Primary Subject Heading**:	Health services research
Secondary Subject Heading:	Public health, Epidemiology, Health policy, Health services research
Keywords:	Quality in health care < HEALTH SERVICES ADMINISTRATION & MANAGEMENT, Organisation of health services < HEALTH SERVICES ADMINISTRATION & MANAGEMENT, Clinical governance < HEALTH SERVICES ADMINISTRATION & MANAGEMENT

SCHOLARONE™
Manuscripts

BMJ

I, the Submitting Author has the right to grant and does grant on behalf of all authors of the Work (as defined in the below author licence), an exclusive licence and/or a non-exclusive licence for contributions from authors who are: i) UK Crown employees; ii) where BMJ has agreed a CC-BY licence shall apply, and/or iii) in accordance with the terms applicable for US Federal Government officers or employees acting as part of their official duties; on a worldwide, perpetual, irrevocable, royalty-free basis to BMJ Publishing Group Ltd ("BMJ") its licensees and where the relevant Journal is co-owned by BMJ to the co-owners of the Journal, to publish the Work in this journal and any other BMJ products and to exploit all rights, as set out in our licence.

The Submitting Author accepts and understands that any supply made under these terms is made by BMJ to the Submitting Author unless you are acting as an employee on behalf of your employer or a postgraduate student of an affiliated institution which is paying any applicable article publishing charge ("APC") for Open Access articles. Where the Submitting Author wishes to make the Work available on an Open Access basis (and intends to pay the relevant APC), the terms of reuse of such Open Access shall be governed by a Creative Commons licence – details of these licences and which Creative Commons licence will apply to this Work are set out in our licence referred to above.

Other than as permitted in any relevant BMJ Author's Self Archiving Policies, I confirm this Work has not been accepted for publication elsewhere, is not being considered for publication elsewhere and does not duplicate material already published. I confirm all authors consent to publication of this Work and authorise the granting of this licence.

For peer review only

Title page

Date: 27 October 2020

Manuscript title:

Associations between Hospital Deaths (HSMR), Readmission and Length of Stay (LOS): a longitudinal assessment of performance results and facility characteristics of teaching and large-sized hospitals in Canada between 2013-14 to 2017-18

Authors:

Omid Fekri (corresponding author: o.fekri@amsterdamumc.nl)

Edgar Manukyan

Niek Klazinga

Affiliation: University Medical Centers - University of Amsterdam

Word count: 4915 (excluding tables, graphs, abstract, references)

For peer review only

Associations between Hospital Deaths (HSMR), Readmission and Length of Stay (LOS): a longitudinal assessment of performance results and facility characteristics of teaching and large-sized hospitals in Canada between 2013-14 to 2017-18

Abstract

Objectives: To examine the association between Hospital Deaths (HSMR), Readmission, Length of Stay (LOS), and eight hospital characteristics.

Design: Longitudinal observational study.

Setting: A total of 119 teaching and large-sized hospitals in Canada between fiscal years 2013–14 and 2017-18.

Participants: Analysis focused on indicator results and characteristics of individual Canadian hospitals.

Primary and secondary outcomes: Hospital Deaths (HSMR); All Patients Readmitted to Hospital; Average Length of Stay (LOS); and a series of eight hospital characteristic summary measures: Number of Acute Care Hospital Stays; Number of Acute Care Beds; Number of Emergency Department Visits; Average Acute Care Resource Intensity Weight; Total Acute Care Resource Intensity Weight; Hospital Occupancy Rate; Patients Admitted Through the Emergency Department (%); Patient Days in Alternate Level of Care (%).

Results: Comparing 2013-14 to 2017-18, Hospital deaths (HSMR) largely declined, while readmissions increased; 69% of hospitals decreased their hospital deaths (HSMR), while 65% of hospitals increased their readmissions rates. A greater proportion of Community-Large hospitals (31%, n=14) improved on both hospital deaths (HSMR) and readmission compared to Teaching hospitals (13.9%, n=5). Hospital deaths (HSMR), readmission and LOS largely showed very weak and non-significant correlations. LOS was largely positively and statistically significantly correlated with the suite of eight hospital characteristics. Hospital deaths (HSMR) was largely negatively (not statistically significantly) correlated with the hospital characteristics. Readmission was largely not statistically significantly correlated and showed no clear pattern of correlation (direction) with hospital characteristics.

Conclusions: Examining publicly-reported hospital performance results can reveal meaningful insights into the association among outcome indicators and hospital characteristics. Good or bad hospital performance in one care domain does not necessarily reflect similar performance in other care domains. Thus, caution is warranted in a narrow use of outcome indicators in the design and operationalization of hospital performance measurement and governance models (namely pay-for-performance schemes). Analysis such as this can also inform quality-improvement strategies and targeted efforts to address domains of care experiencing declining performance over time; further granular subdivision of the analyses, for example by hospital peer-groups, can reveal notable differences in performance.

Article Summary - Strengths and limitations of this study

- Assessed correlations across eight hospital characteristics and three hospital performance indicators.
- Assessed five years of performance data.

For peer review only

- Examined the majority of Teaching and Community-Large hospitals in Canada.
- Inability to apply more complex statistical modelling techniques due to limitations on the use of aggregate hospital-level data in secondary analyses.
- LOS is an aggregate of all hospitalizations, and could not be restricted to condition-specific cases (of hospital death or readmission).

For peer review only

Introduction

Over the last two decades, there has been substantial interest in hospital performance[1], and with financing of hospitals increasingly tied to improving the quality of care delivered[2]. Along with improving the quality of care, a tandem goal of hospital reforms has been to improve efficiency[3] (i.e., reducing waste, streamlining care pathways, increasing patient throughput, optimizing the use of technology, etc.). Hospital deaths[4] and readmission to hospital[5] are among the most commonly used indicators to measure quality of hospital care, while average Length of Stay (LOS) is often used as a measure of efficiency[6]. The three measures together (hospital deaths, readmission and LOS) have been the subject of increased interest in recent years to assist with more reliable interpretations of hospital performance[7].

However, the goals of achieving quality and efficiency can at times be opposing. For example, it seems warranted to investigate whether a hastened hospital stay (shorter LOS) would lead to an increased chance of readmission to hospital[8]. Similarly, do efforts to reduce hospital readmissions have the unintended consequence of increasing the likelihood of mortality after hospitalization[9]? While hospital deaths and readmission are both desired to be reduced, it is not definite (and varying across diseases and clinical procedures) whether a patient's LOS should be lower or higher in order to minimize readmission or in-hospital mortality. However, what can be deduced is that the relationships between LOS, in-hospital mortality and readmission are intertwined and interdependent. Hence governance of hospitals based on these publicly reported indicators should be based on acknowledgment and consideration of these interdependencies.

Yet, despite a sizeable research community investigating the interrelationship between these indicators, the evidence-base on the patterns of these interdependencies remains inconclusive due to wide heterogeneity in methods and findings across studies (which speaks to the complexity of the topic). For example, a switch between the unit of analysis (from patient-level to hospital-level), on the same underlying admissions data, will yield inconsistent, and even inverse, results[10]. In recent years, researchers have also examined hospital characteristics, such as hospital volumes[11] or hospital teaching status[12] to better understand any associations between LOS, readmission and in-hospital mortality.

Much of the afore cited literature originates from the United States and Europe. With a scarcity of local examples, this study used a large, nationally-representative dataset of hospital performance measures (produced by the Canadian Institute for Health Information (CIHI)) to expand interest and add evidence for the Canadian context. Specifically, we investigate the relationship between hospital deaths, readmission and LOS, and explore any associations with hospital characteristics. Our specific research questions are:

1. How have hospitals performed in both the hospital deaths (HSMR) and readmission indicators over time?
2. What is the correlation between hospital deaths (HSMR), readmissions and LOS?
3. How do a series of eight hospital characteristics correlate with hospital deaths (HSMR), readmissions and LOS?
4. Do the results of the aforementioned research questions show differences between peer groups of Teaching hospitals and Community-Large hospitals?

For peer review only

Methods

Data

We used the all data export report file from CIHI's Your Health System In Depth online tool[13] to perform the analyses. The data file contains results per hospital for all indicators published on the online tool as well as contextual measures and additional variables to assist with analysis and interpretation. Five singleton fiscal year (1 April to 31 March) data points were available covering 2013–14 to 2017–18 for the indicators capturing Hospital Deaths (HSMR) and All Patients Readmitted to Hospital (henceforth referred to 'Readmission'), while LOS and eight hospital characteristics measures were only available for the most recent year (2017-18).

Definition of variables

The following indicators were used for the analysis: Hospital Deaths (HSMR) (Hospital Standardized Mortality Ratio), Readmission (%), and LOS (days); and eight contextual measures of hospital facility characteristics: Number of Acute Care Hospital Stays; Number of Acute Care Beds; Number of Emergency Department Visits; Average Acute Care Resource Intensity Weight (RIW); Total Acute Care RIW; Hospital Occupancy Rate; Patients Admitted Through the Emergency Department; Patient Days in Alternate Level of Care (%).

HSMR (hospital standardized mortality ratio) and other variations of summary hospital mortality measures are commonly-used indicators to assess hospital performance. The Hospital Deaths (HSMR) indicator is a ratio of observed to expected in-hospital mortality, capturing the 72 leading causes of hospital death (representing ~80% of all in-hospital mortality). The Readmission indicator captures all urgent patient readmissions within 30-days. The average LOS indicator is a sum of all valid days spent in hospital, divided by the total number of inpatient cases. Detailed technical notes on these indicators[14], and on hospital facility characteristics[15], are made available by CIHI through its Indicator Library.

Both hospital deaths (HSMR) and readmission indicators are risk-adjusted. Hospital deaths (HSMR) risk-adjustment variables are: age, sex, LOS, admission category, comorbidity (Charlson Index Score), and transfers. As the Readmission indicator is an aggregate of four sub-categories of readmission (medical, surgical, obstetric, paediatric), the Readmission risk-adjustment variables are not constant across the four sub-categories; this range of risk-adjustment variables for are: age, sex, acute care hospitalizations in previous 6 months, admission category, comorbidity (Charlson Index Score), and case-mix groupings. Detailed information on model specifications and coefficients used in calculations are available elsewhere[16, 17].

CIHI classifies the approximately 600 hospitals in Canada into four distinct peer-group types: Teaching hospitals; Community—Large hospitals; Community—Medium hospitals; and Community—Small hospitals. This classification facilitates meaningful comparisons across hospitals of similar structural characteristics, patient volume, and clinical complexity[18]. Since characteristics of hospitals are not included in risk-adjustment models, any comparison of two or more hospitals' individual performance should be done within their respective hospital peer-groups.

A hospital is designated as 'Teaching' by provincial/territorial ministries of health, or was identified as such in the provincial/territorial ministry's submission to CIHI's Management Information System (MIS) Database. Community—Large hospitals meet two of the following three criteria: more than 8,000 inpatient cases; more than 10,000 weighted cases; or more than 50,000 inpatient days.

In order to qualify for public-reporting of results for the Hospital Deaths (HSMR) indicator, a hospital must meet a minimum of 2,500 eligible Hospital Deaths (HSMR) cases for each of the most recent three consecutive

For peer review only

years[19]. Consequently, no Community—Small hospitals met this criteria to have publicly-reported Hospital deaths (HSMR) results. Of the 93 Community—Medium hospitals only 11 hospitals met the minimum reporting requirements and had Hospital deaths (HSMR) results reported. Since this represents only 8.5% of the entire peer-group, it was decided to also exclude Community—Medium hospitals, alongside Community—Small hospitals, in this analysis. Hospitals with only one year of data available, for both Readmission and Hospital Deaths (HSMR) indicators, for either 2013-14 or 2017-18 only, were excluded from performance trend analysis. Therefore, a total of 119 hospitals were included in the overall study, 53 Teaching hospitals and 66 Community-Large hospitals (representing 67.9% and 68.2% of all hospitals in their respective peer-group totals in the available online dataset). A subset of 81 hospitals were included in the performance trend analysis.

Statistical analyses

Descriptive statistics for the analysis of LOS, Hospital Deaths (HSMR) and Readmission indicators are presented by range of values, peer-group means and 95% confidence intervals (CI), and coefficient of variation (CoV) (see Table 1). Trend over time is calculated as the percent-change difference between first and last year of data (2013-14 and 2017-18). A paired-t test was used to determine whether absolute changes in rates between 2013-14 and 2017-18 were significant.

To compare indicator rates per hospital across 2013-14 to 2017-18, three possible outcomes are inferred: a decrease in rate (2013-14 > 2017-18); an increase in rate (2013-14 < 2017-18); and no change in rate (2013-14 = 2017-18). Multiplying these three outcomes by the two indicators of interest (Hospital Deaths (HSMR) and Readmission), in tandem, yields a total of nine trend outcomes (see Table 2).

Graphical representation of the aforementioned tests are shown via scatterplots depicting: 1) percent-change over time for Hospital Deaths (HSMR) and Readmission (delineated by peer-group) (see Figure 1); and 2) 2017-18 data year results on Hospital Deaths (HSMR) and Readmission, with LOS depicted as the size of the bubble plot (see Figures 2 & 3).

A Spearman's Rank Correlation test examines the association between LOS, Hospital Deaths (HSMR) and Readmission on 2017-18 data year values (with breakdowns for Teaching and Community—Large hospital peer-groups). Strengths of correlations, the absolute value of R_s (positive and negative) are defined as: .00-.19 very weak; .20-.39 weak; .40-.59 moderate; .60-.79 strong; .80-1.0 very strong[20].

Lastly, a Spearman's Rank Correlation test was also used to assess the correlation between eight hospital facility characteristics against LOS, Hospital Deaths (HSMR) and Readmission values for 2017-18. All analyses were performed on R version 3.5.0 (R Foundation for Statistical Computing, Vienna, Austria).

Patient and public involvement

Patients or public were not involved in the design of this longitudinal, observational study. However, all data used are available in the public domain.

For peer review only

Results

Combined performance of hospital mortality (HSMR) and readmission over time

In comparing 2013-14 and 2017-18 indicator rates, Hospital deaths (HSMR) showed a mean improvement of lowering rates, while readmissions showed a mean increase in rates (see table 1). A paired-t test showed statistically significant changes in trend over time for both indicators: hospital deaths (HSMR) improved by a mean of -5.1 (95% CI, -7.33 to -2.9, p<.001), and readmission rates increased by a mean of 0.15% (95% CI, 0.04 to 0.26, p=.006). While not statistically significant, the Community-Large hospital peer-group showed a greater mean improvement in hospital deaths (HSMR) by -6.0% (95% CI -9.1 to -2.8), while Teaching hospitals improved by -4.1% (95% CI -7.5 to -0.8). Both hospital peer groups experienced a mean increase in readmission rates, with Community-Large hospitals at 1.6% (95% CI -0.3 to 3.4), and Teaching hospitals at 2.1% (95% CI 0.7 to 3.6). When examining the 2017-18 data year, Community-Large hospitals had a statistically significant lower rate of readmissions at 8.9 (95% CI, 8.7 to 9.1) compared to Teaching hospitals at 9.4 (95% CI, 9.2 to 9.6). Table 2 provides a lens on how individual hospitals performed in both indicators. Nine possible outcomes of performance are shown. Overall, 56 (69%) out of the total 81 hospitals assessed decreased their hospital deaths (HSMR), while only 23 (28%) hospitals decreasing their readmissions rates.

Figure 1 illustrates the combined percent change of hospital deaths (HSMR) and readmissions rates (comparing 2013-14 and 2017-18 individual hospital rates) delineated by hospital peer group. While coefficient of variation values are largely similar between the two peer groups for the two outcome indicators, nearly three times as many Community-Large hospitals (n=14) showed greater improvement in the bottom left quadrant of Figure 1 (decrease in both hospital deaths (HSMR) and readmission), than Teaching hospitals (n=5). These clear trends of overall decreasing hospital deaths and rising readmissions have been confirmed in our previous analysis[21].

For peer review only

Table 1 Descriptive statistics for combined analysis of hospital deaths (HSMR), readmission and LOS

	Teaching hospitals				Community-large hospitals			
**Number of hospitals, n**	36				45			
**Range of values for 2017-18 data year**	**Range of values**	**Teaching Peer-group mean* (95%CI)**	**Coefficient of variation, % (95%CI)**	**Median (IQR)**	**Range of values**	**Community-large Peer-group mean* (95%CI)**	**Coefficient of variation, %(95%CI)**	**Median (IQR)**
**LOS (days)**	4.6 to 9.2	7.1 (6.7 to 7.4)	16 (13 to 21)	6.9 (1.4)	4.5 to 13.7	6.5 (6.1 to 6.9)	24 (20 to 29)	6.2 (1.4)
**Hospital Deaths (HSMR)**	66 to 118	91.8 (87.8 to 95.7)	14 (11 to 18)	92 (18)	65 to 144	87.5 (83.9 to 91)	16 (13 to 19)	86 (19.5)
**Readmission (%)**	7.4 to 10.6	9.4 (9.2 to 9.6)	8 (7 to 11)	9.5 (0.9)	7.4 to 10.7	8.9 (8.7 to 9.1)	8 (7 to 10)	8.8 (0.8)
**Percent-change difference 2013-14 vs. 2017-18 (%)**	**Range of % change**	**Mean Teaching Peer-group % change* (95%CI)**			**Range of % change**	**Mean Community-large Peer-group % change* (95%CI)**		
**Hospital Deaths (HSMR)**	-21 to 22	-4.1 (-7.5 to -0.8)			-33 to 21	-6.0 (-9.1 to -2.8)		
**Readmission (%)**	-12 to 12	2.1 (0.7 to 3.6)			-14 to 17	1.6 (-0.3 to 3.4)		

*calculated by summing values of all hospitals within peer-group and dividing by number of hospitals

Table 2 Hospital outcomes on HSMR and Readmission changes over time

Trend outcome	Hospital deaths (HSMR)	Readmission	Teaching hospitals (total n=36) Number, (%)	Community-Large hospitals (total n=45) Number, (%)	Total of all hospitals, number, (%)
Decrease in both HSMR & Readmission	↓	↓	5 (13.9%)	14 (31.1%)	19 (23.5%)
Decrease in HSMR, increase in Readmission	↓	↑	20 (55.6%)	14 (31.1%)	34 (42.0%)
Decrease in HSMR, no change in Readmission	↓	=	1 (2.8%)	2 (4.4%)	3 (3.7%)
Increase in HSMR, decrease in Readmission	↑	↓	2 (5.6%)	1 (2.2%)	3 (3.7%)
Increase in both HSMR & Readmission	↑	↑	7 (19.4%)	8 (17.8%)	15 (18.5%)
Increase in HSMR, no change in Readmission	↑	=	1 (2.8%)	0	1 (1.2%)
No change in HSMR, decrease in Readmission	=	↓	0	1 (2.2%)	1 (1.2%)
No change in HSMR, increase in Readmission	=	↑	0	4 (8.9%)	4 (4.9%)
No change in both HSMR & Readmission	=	=	0	1 (2.2%)	1 (1.2%)

↑=signifies increasing rate; ↓=signifies decreasing rate; = signifies no change

For peer review only - http://bmjopen.bmj.com/site/about/guidelines.xhtml

Figure 1 Scatterplot of percent change between 2013-14 – 2017-18 for Readmission and HSMR (by hospital peer-group)

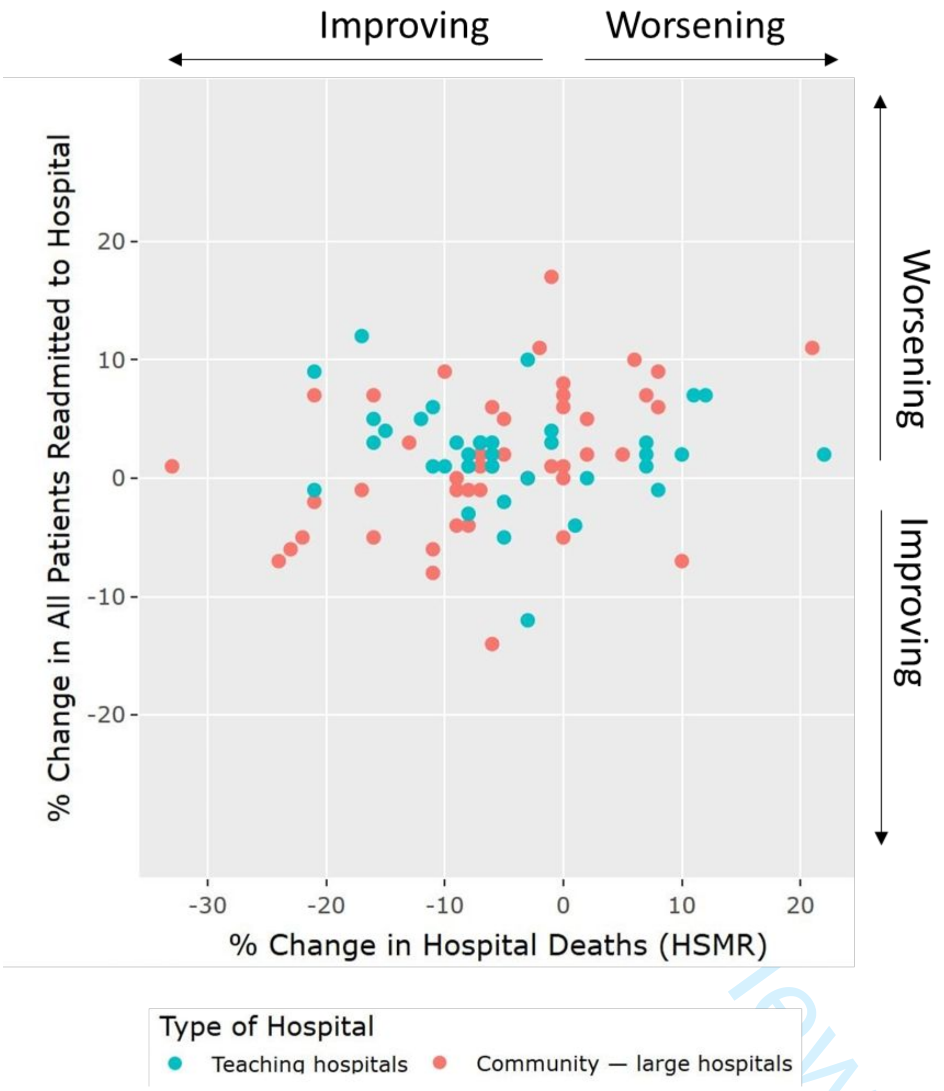

Hospital deaths (HSMR), readmissions and LOS (2017-18)

In examining hospital deaths (HSMR), readmission and LOS for potential associations, only very weak to weak non-statistically significant results were observed. (see table 3). While Community-Large hospitals showed greater variation in LOS values compared to Teaching hospitals (CoV=24% compared to 16%), their mean peer group LOS values were still lower than Teaching hospitals (6.5 days compared to Teaching hospitals at 7.1) (see table 1). Mean LOS of patients in Community-Large hospitals was 0.6 days shorter, or roughly half a day, compared to Teaching hospitals (6.5 vs. 7.1 days). Figures 2 and 3 illustrate LOS, hospital deaths (HSMR) and readmission values for the 2017-18 data year (with LOS delineated in size and shading of bubble plot).

Table 3 Correlations between Hospital Deaths (HSMR), Readmission and LOS (breakdowns by Teaching and Community-Large hospitals) ***(2017-18)***

	LOS		Hospital deaths (HSMR)	
**Readmission**	Teaching:	**-0.04 (-0.41 to 0.33)**	Teaching:	**0.22 (-0.09 to 0.54)**
	Community-Large:	**0.04 (-0.23 to 0.31)**	Community-Large:	**-0.13 (-0.42 to 0.15)**

* p less than .01; ^ p less than .05; Direction of correlation is shown as Blue (positive) and Red (negative), and intensity of cell-colouring reflects strength of correlation. Correlation strength classification: .00-.19 very weak; .20-.39 weak; .40-.59 moderate; .60-.79 strong; .80-1.0 very strong.

For peer review only - http://bmjopen.bmj.com/site/about/guidelines.xhtml

Figure 2 Scatterplot of Teaching hospital values for Hospital Deaths (HSMR), Readmission and LOS (2017-18)

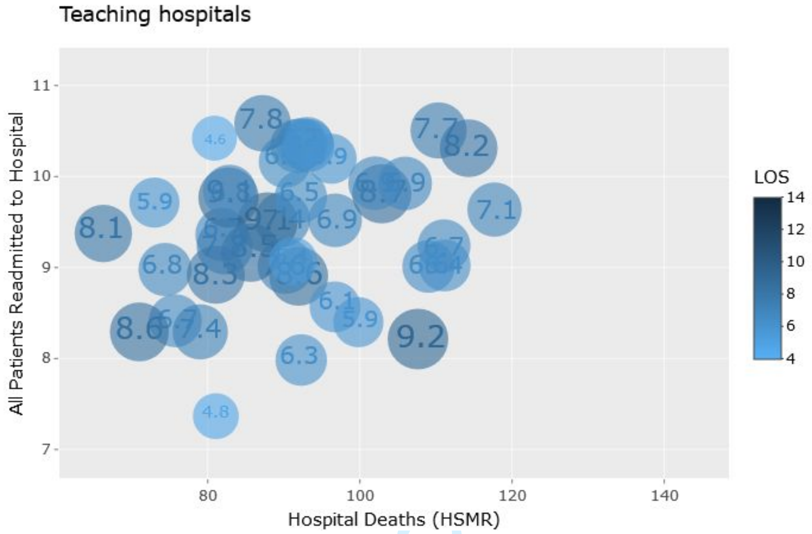

Figure 3 Scatterplot of Community-large hospital values for Hospital Deaths (HSMR), Readmission and LOS (2017-18)

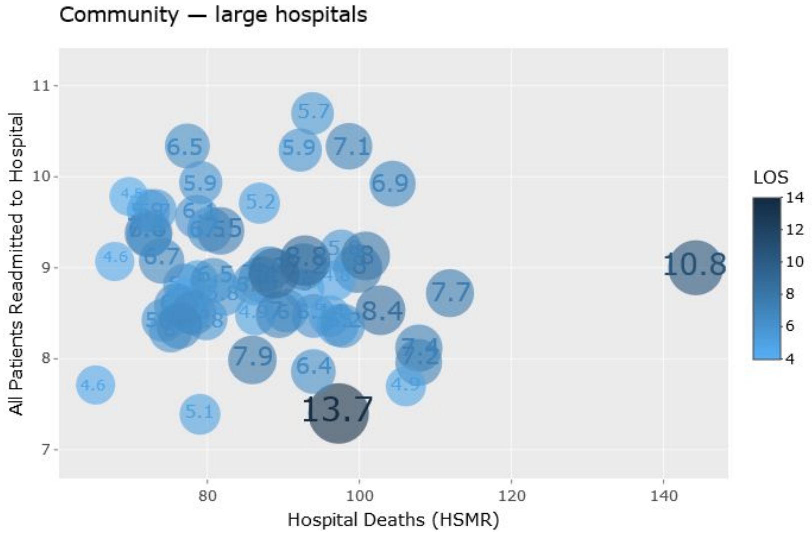

Correlation between hospital characteristics, LOS, Hospital Deaths (HSMR) and Readmission

Table 4 Correlations between hospital characteristics on LOS, HSMR and Readmission ***(2017-18)***

Hospital characteristic	Unit	Length of stay Correlation coefficient (95% CI)		Hospital deaths (HSMR) Correlation coefficient (95% CI)		Readmission Correlation coefficient (95% CI)	
**Number of Acute Care Hospital Stays**	# of days	All:	-0.04 (-0.24 to 0.16)	All:	-0.14 (-0.34 to 0.05)	All:	0.07 (-0.12 to 0.26)
		Teaching:	0.26 (-0.02 to 0.54)	Teaching:	-0.30 (-0.61 to 0.01)	Teaching:	0.07 (-0.23 to 0.37)
		Community-Large:	-0.36* (-0.59 to -0.13)	Community-Large:	-0.20 (-0.45 to 0.05)	Community-Large:	-0.11 (-0.36 to 0.15)
**Number of Acute Care Beds**	# of beds	All:	0.24* (0.05 to 0.42)	All:	-0.01 (-0.20 to 0.19)	All:	0.03 (-0.16 to 0.22)
		Teaching:	0.50* (0.23 to 0.76)	Teaching:	-0.24 (-0.54 to 0.07)	Teaching:	-0.03 (-0.35 to 0.29)
		Community-Large:	-0.02 (-0.24 to 0.20)	Community-Large:	0.01 (-0.25 to 0.26)	Community-Large:	-0.17 (-0.41 to 0.07)
**Number of Emergency Department Visits**	# of visits	All:	-0.13 (-0.37 to 0.10)	All:	0.03 (-0.21 to 0.27)	All:	0.04 (-0.18 to 0.27)
		Teaching:	0.17 (-0.20 to 0.55)	Teaching:	-0.14 (-0.53 to 0.26)	Teaching:	0.18 (-0.16 to 0.52)
		Community-Large:	-0.44* (-0.70 to -0.17)	Community-Large:	0.13 (-0.20 to 0.46)	Community-Large:	-0.20 (-0.49 to 0.09)
**Average RIW**	Average RIW	All:	0.68* (0.56 to 0.80)	All:	0.39* (0.20 to 0.57)	All:	0.15 (-0.04 to 0.35)
		Teaching:	0.55* (0.31 to 0.80)	Teaching:	0.00 (-0.31 to 0.31)	Teaching:	0.12 (-0.20 to 0.45)
		Community-Large:	0.76* (0.62 to 0.89)	Community-Large:	0.53* (0.32 to 0.74)	Community-Large:	-0.20 (-0.44 to 0.05)
**Total RIW**	Total RIW	All:	0.13 (-0.06 to 0.33)	All:	-0.02 (-0.22 to 0.17)	All:	0.13 (-0.06 to 0.32)
		Teaching:	0.43* (0.16 to 0.70)	Teaching:	-0.25 (-0.55 to 0.06)	Teaching:	0.11 (-0.20 to 0.41)
		Community-Large:	-0.16 (-0.40 to 0.08)	Community-Large:	-0.06 (-0.32 to 0.19)	Community-Large:	-0.13 (-0.39 to 0.12)
**Hospital Occupancy Rate**	% of occupancy	All:	0.09 (-0.12 to 0.29)	All:	-0.14 (-0.37 to 0.08)	All:	0.01 (-0.20 to 0.23)
		Teaching:	0.37^ (0.07 to 0.67)	Teaching:	-0.28 (-0.61 to 0.06)	Teaching:	0.00 (-0.34 to 0.34)
		Community-Large:	-0.12 (-0.39 to 0.14)	Community-Large:	-0.10 (-0.41 to 0.21)	Community-Large:	0.01 (-0.27 to 0.29)
**Patients Admitted Through the Emergency Department**	% of patients	All:	0.30* (0.13 to 0.48)	All:	-0.11 (-0.31 to 0.08)	All:	0.12 (-0.08 to 0.31)
		Teaching:	0.47* (0.18 to 0.75)	Teaching:	-0.04 (-0.41 to 0.32)	Teaching:	0.29^ (0.00 to 0.58)
		Community-Large:	0.39* (0.16 to 0.61)	Community-Large:	-0.10 (-0.36 to 0.16)	Community-Large:	0.27^ (0.03 to 0.52)

For peer review only - http://bmjopen.bmj.com/site/about/guidelines.xhtml

**Patient Days in Alternate Level of Care**	%	All:	0.23^ (0.03 to 0.43)	All:	-0.01 (-0.24 to 0.22)	All:	-0.29* (-0.50 to -0.09)
		Teaching:	0.36^ (0.06 to 0.66)	Teaching:	0.02 (-0.37 to 0.42)	Teaching:	-0.28 (-0.62 to 0.05)
		Community-Large:	0.24 (-0.04 to 0.52)	Community-Large:	0.07 (-0.27 to 0.40)	Community-Large:	-0.13 (-0.43 to 0.17)

* p less than .01; ^ p less than .05; Direction of correlation is shown as Blue (positive) and Red (negative), and intensity of cell-colouring reflects strength of correlation.
Correlation strength classification: .00-.19 very weak; .20-.39 weak; .40-.59 moderate; .60-.79 strong; .80-1.0 very strong.
RIW (Acute Care Resource Intensity Weight)

For peer review only

Table 4 shows the correlation between hospital characteristics and LOS, hospital deaths (HSMR) and readmissions. LOS was largely positively correlated (and statistically significant) with the series of eight hospital characteristics. Hospital deaths (HSMR) was largely weak to very weakly negatively correlated. Readmissions were mixed with positive and negative weak to very weak correlations. Correlations between Hospital deaths (HSMR) and readmissions with the eight hospital characteristics were largely not statistically significant (aside from patient days in alternate level of care, patients admitted through the emergency department, and average acute care RIW).

The Number of Acute Care Hospital Stays was only statistically significantly correlated with LOS (negatively weakly) in Community-Large hospitals (r= -0.36, p < 0.01). Teaching hospitals had a moderate positive and statistically significant correlation in the Number of Acute Care Beds and LOS (r =0.5, p <0.01). The Number of Emergency Department Visits and LOS were negatively moderately correlated in Community-Large hospitals (r= -0.44, p <0.01). The Average Acute Care RIW was positively strongly correlated with LOS (r= 0.68, p <0.01) when assessing both hospital peer groups. With respect to hospital deaths (HSMR), the average acute care RIW was positively moderately correlated in Community-Large hospitals (r=0.53, p <0.01). Total Acute Care RIW was only moderately positively correlated with LOS for Teaching hospitals (r=0.43, p <0.01). Hospital Occupancy Rate was only statistically significantly correlated with LOS for Teaching hospitals (r=0.37, p <0.05). With respect to hospital deaths (HSMR), a hospital's occupancy rate is very weak to weakly negatively correlated (and not statistically significant). Patients Admitted Through the Emergency Department had a positive weak to moderate correlation with LOS (Teaching hospitals r=0.47, p <0.01; Community-large hospitals r=0.39, p <0.01), and a positive weak correlation with readmissions (Teaching hospitals r=0.29, p <0.05; Community-large hospitals r=0.27, p <0.05). The percentage of Patient Days in Alternate Level of Care (a measurement of days patients spend in inpatient acute care, when unneeded, while waiting for discharge to home care or other supports are ready) had a positive weak correlation with LOS in Teaching hospitals (r=0.36, p <0.05), and a weak negative correlation with readmissions for all hospitals combined (r=-0.29, p <0.01).

Supplementary data files include descriptive statistics (mean/percent-change values, CIs, range of values, and number of hospitals) by indicator, facility characteristics, provincial/territorial jurisdiction, and hospital type/size, and correlation matrix scatterplots.

Discussion

In recent years, there has been growing interest in the association between hospital deaths, readmission and LOS[7]. It is logical to investigate the strength and directionality of correlation between these three components of hospital performance, and with hospital characteristics. There is wide heterogeneity in the available evidence in this research area. Aside from the natural differences across studies that narrow their scope in terms of disease or procedure-specific indicators, limited clinical settings within hospitals, and small denominator groups, even a change in the unit of analysis on the same underlying data, from patient-level data to hospital-level data, can yield disparate results[10].

This secondary analysis of hospital performance data aimed to provide a high-level overview of the association between hospital deaths, readmission and LOS across a majority of Teaching and Community-Large hospitals in Canada between 2013-14 and 2017-18. The classification and assignment of hospital peer groups allows

For peer review only

for more meaningful and valid comparisons of performance of hospitals across similar structural characteristics, patient volumes, and clinical services offered. Therefore, any comparison of individual hospital performance should be restricted to within a respective peer-group. Delineating the results of this study's analyses by Teaching and Community-Large hospitals allows for a more granular interpretation of hospital performance at peer group level. Of the three outcome indicators, only with the readmissions indicator was there a statistically significant result of Community-Large hospital peer-group showing a lower peer-group average than that of the Teaching peer-group. Detailed data on eight hospital characteristics were also available in the dataset published by the data steward. As this study was exploratory in nature, we additionally included these hospital characteristics in the correlation analyses to explore any meaningful relationships with the aforementioned three main indicators, and delineated by hospital peer group type.

Our earlier research[21] established that, over time, Canadian hospitals have largely improved on in-hospital mortality; readmission rates have been trending upward; and that good or bad performance in one domain of care does not automatically reflect the same performance in other domains. What this present study aimed to add is whether a hospital's improvement or weakening performance over time, in either hospital deaths (HSMR) or readmission, had a positive or negative association on the other; our results showed that 42% of hospitals, the largest proportion across the possible outcomes, in fact decreased hospital deaths (HSMR) while increasing readmission rates. Furthermore, we added LOS to the research question as a proxy of hospital efficiency. Eight hospital characteristics showed trends in strength and directionality of correlation with hospital deaths (HSMR), readmission and LOS. As this study was exploratory in nature, in both using aggregate hospital-level data and hospital characteristics in the analyses, we did not have an explicit hypothesis on the degree of association between hospital characteristics and the three outcome indicators. We note (and continued to include in the analyses) an outlier hospital (see Figure 3) with a high Hospital Deaths (HSMR) indicator value, a long LOS, and average Readmission rate.

Strengths and limitations of this study

The main strengths of this study are the quality and extent of data used; all Teaching and Community-Large hospitals across Canada that had publicly-available reported performance results were included in the analysis. The 'all readmission' indicator captures, as the title suggests, all readmission to hospital within 30-days; the hospital deaths (HSMR) indicator captures ~80% of all in-hospital mortality; and the LOS indicator quantifies the mean duration across all hospitalizations. Eight diverse hospital characteristics also provided summary measures that capture numerous aspects of a hospital's performance context. While results for LOS and the eight hospital characteristics were only available for the most-recent year (2017-18), for hospital deaths (HSMR) and readmission indicators, five fiscal year data points were available to measure trend over time differences.

There are limitations in this study with respect to its generalisability beyond Canada; differences in risk-adjustment methodologies, indicator definitions and calculation methods, and hospital type/size definitions, pose challenges to make apples-to-apples comparisons across countries. However, the categorical outcomes of performance simultaneously comparing hospital deaths and readmission, along with the correlation tests of these indicators and hospital characteristics, is available and worthwhile to other settings. Community-Medium and Community-Small hospitals in Canada treat fewer patients, and offer less-complex clinical services. This large group of hospitals (comprising of more than half within the country) are omitted from this study due to an absence of publicly-reported indicator values for hospital deaths. Furthermore, as a result of mergers between disparate

For peer review only

hospitals, historic indicator values (i.e., 2013-14 data year) are omitted from the reporting platform. Thus, this inhibits a longitudinal comparison (i.e., performance trend over time). However, current indicator values and hospital characteristics data is available and was included in analyses that only required 2017-18 data year (namely, correlation analyses on hospital characteristics).

An important limitation of this study, inherent to the constraints of using aggregate-level hospital data, is the inability to perform more complex analyses. Previous, more granular analyses by researchers have been able to employ more sophisticated statistical techniques, including modelling, controlling for confounding factors, calculation of composite indicators, application of more refined case inclusion/exclusion criteria, and stratification of analyses across different disease groups. Another such example of a limitation exists with the LOS measure reflecting the average of all hospitalizations, and the inability to select just those applicable to Hospital Deaths (HSMR) or Readmission patients respectively. Acknowledging these limitations of performing secondary analyses on aggregate, publicly-available hospital performance data, we nonetheless pursued our four research questions, with the data available at hand, to determine what, if any, level of association exists at the hospital indicator level.

The two main outcome indicators themselves, Hospital Deaths (HSMR) and Readmission, also have methodological limitations due to the inability of including non-hospital death data. The Hospital Deaths (HSMR) indicator, unlike the Summary Hospital-level Mortality Indicator (SHMI), can only account for deaths that occur in hospitals. Similarly, the Readmission indicator cannot exclude patients from the denominator that have passed away in the community following hospital discharge. While the indicators of Hospital Deaths (HSMR) and Readmission are risk-adjusted (as described in the Methods section), not all risk-factors can be adjusted for (due to reasons such as viability)[22]. For example, detailed data on patient socio-demographics or access to primary care services is unavailable for risk-adjustment modelling. Lastly, as correlation does not equal causation, the correlation-based results of this study should be interpreted with caution.

Reflections on the study's findings

Public reporting of performance results poses challenges to hospital administrators and the broader public. Public reporting has become a staple in health systems and hospital performance management. But the practice of public reporting is not without concerns[23]. Tunnel vision and myopia by hospital governance and performance managers can run the risk of sub-optimisation; the unintended consequences of shifting concentration disproportionately towards areas prioritized for immediate measurement at the expense of other areas of care and broader/long-term organizational goals[24].

Pay for performance schemes are commonplace in hospital governance. A governance model that assesses hospitals through isolated performance measures, runs the risk of unintended consequences in other factors of care and performance not under immediate scrutiny[8]. The results and methods of this study support the notion that quantification of hospital performance should not be done via isolated or single measures at a time, but rather in a more broad and informed mechanism of considering complementary aspects of hospital performance (such as those in the CIHI Hospital Performance Framework: access to services, clinical effectiveness, safety, coordination of care, patient-centeredness, and hospital efficiency)[25]. Furthermore, a poorly conceptualized pay-for-performance scheme may be mal-aligned to take into consideration the correlation (and potential causality) of intensifying efforts to reduce, for example, LOS or hospital mortality, on the increase of readmission rates.

Moreover, government officials charged with hospital governance must take into account inequality across hospital facilities and hospital corporations. Beginning in the 1990s, but increasing rapidly in recent years, there has

For peer review only

been a trend of mergers between multiple hospitals and between hospitals and rehabilitation institutes into a singular hospital corporation[26]. These larger hospital corporations in turn have near-exclusive coordination of care between acute-care patients served in hospitals and subsequently their transfer to rehabilitation services. Rural and more-remote hospitals (especially those without paired rehabilitation services) could face higher LOS and occupancy rates, greater number of days and percentage of patients in alternate level of care, and greater resource utilization. If analysis of these amalgamated hospitals and rehabilitation services proves they perform better than hospitals without direct rehabilitation services, this consideration should also be included in the contextual interpretation (and perhaps risk-adjustment) of hospital performance and governance. Similarly, readmission to hospital may also be a proxy of the strength and availability of primary health care services in the community. Thus, the necessity to consider hospital performance in the broader context of an integrated health service delivery system, a tenet of the accountable care organization movement[27].

Government bodies and professional associations charged with supporting quality improvement initiatives can use the methods and findings of this type of analysis to identify best practices and top-performing hospitals so as to learn from their effective practices. Similarly, hospitals in an unfavourable quadrant (long LOS, and high hospital mortality and readmissions) should receive tailored programs to support their improvement in quality and efficiency of care.

The general public, too, requires consideration when publicly reporting performance results. Efforts in describing indicators in plain language and providing a framework for contextualization can increase the public's assimilation of performance results (especially demographic groups with fewer skills or resources)[28]. CIHI's applies these practices in their online YHS tool, providing their health system performance[29] and hospital performance frameworks[25] as a basis for the curation of performance results, and describing both performance indicators and hospital characteristics in plain language.

The results of this study do not provide a definitive outcome to the debate on the complementarity between LOS, hospital deaths, readmission and hospital characteristics. The underlying pathways and differences between hospitals in functions, and scope of services provided, makes the hospital a complex unit of analyses. The corpus of past studies illustrates the wide heterogeneity of research methods and degree of association outcomes. The embedding of this type of analysis into hospital governance formulation can only better-inform those charged with policy-making, and administrators of hospitals. Subdividing the research methods of this study, into disease and/or procedure-specific analysis, can help facilitate addressing quality improvement concerns on specific clinical areas; but caution is stressed so as to not unintentionally cause clinicians and hospital administrators to experience tunnel vision.

Conclusions

This study shows that secondary analyses of publicly-reported hospital performance results can reveal meaningful insights into the association among outcome indicators and hospital characteristics. Good or bad hospital performance in one care domain does not necessarily reflect similar performance in other care domains. Thus, caution is warranted in a narrow use of outcome indicators in the design and operationalization of hospital performance measurement and governance models (namely pay-for-performance schemes). Analysis such as this can also inform quality-improvement strategies and targeted efforts to address domains of care experiencing declining performance over time; further granular subdivision of the analyses, for example by hospital peer-groups, can reveal notable differences in performance.

For peer review only

Contributors:

OF initially conceived of the study, reviewed the literature, performed data analysis, interpreted results, and drafted the manuscript. EM assisted in the design of the study, performed and validated data analysis, interpreted results, and reviewed the manuscript. NK assisted with the design of the study, interpreted results, and assisted in the drafting of the manuscript.

Funding:

This research received no specific grant from any funding agency in the public, commercial or not-for-profit sectors.

Competing interests:

None declared. The results and views expressed are those of the authors alone.

Patient consent for publication:

Not required.

Ethics approval:

Not required.

Provenance and peer review:

Not commissioned; externally peer reviewed.

Data availability statement:

All hospital performance and characteristics data used in this study is publicly available via CIHI's Your Health System online tool (http://yourhealthsystem.cihi.ca/).

For peer review only

References

[1] Oliver Groene, Jutta K. H. Skau, Anne Frølich; An international review of projects on hospital performance assessment, International Journal for Quality in Health Care, Volume 20, Issue 3, 1 June 2008, Pages 162–171,

[2] Helene Eckhardt, Peter Smith, Wilm Quentin. (2019). Pay for Quality: using financial incentives to improve quality of care. In R. Busse, N. Klazinga, D. Panteli, W. Quentin (Eds). Improving healthcare quality in Europe. Characteristics, effectiveness and implementation of different strategies. WHO Regional Office Europe. Copenhagen 2019, pp. 233-264.

[3] Litvak, Eugene & Bisognano, Maureen. (2011). More Patients, Less Payment: Increasing Hospital Efficiency In The Aftermath Of Health Reform. Health affairs (Project Hope). 30. 76-80. 10.1377/hlthaff.2010.1114.

[4] Shahian, D. M., Iezzoni, L. I., Meyer, G. S., Kirle, L., & Normand, S.-L. T. (2012). Hospital-wide Mortality as a Quality Metric: Conceptual and Methodological Challenges. American Journal of Medical Quality, 27(2), 112–123.

[5] Fischer C, Lingsma HF, Marang-van de Mheen PJ, Kringos DS, Klazinga NS, Steyerberg EW. Is the readmission rate a valid quality indicator? A review of the evidence [published correction appears in PLoS One. 2015;10(2):e0118968]. PLoS One. 2014;9(11).

[6] Cylus, Jonathan, Papanicolas, Irene and Smith, Peter C. (2016) Conclusions. In: Cylus, Jonathan, Papanicolas, Irene and Smith, Peter C., (eds.) Health system efficiency: How to make measurement matter for policy and management. Health Policy Series. European Observatory on Health Systems and Policies, Brussels, Belgium, pp. 225-241.

[7] Lingsma HF, Bottle A, Middleton S, et al. Evaluation of hospital outcomes: the relation between length-of-stay, readmission, and mortality in a large international administrative database. BMC Health Serv Res. 2018;18(1):116.

[8] Kaboli PJ, Go JT, Hockenberry J, et al. Associations Between Reduced Hospital Length of Stay and 30-Day Readmission Rate and Mortality: 14-Year Experience in 129 Veterans Affairs Hospitals. Ann Intern Med. 2012;157:837–845.

[9] Dharmarajan K, Wang Y, Lin Z, et al. Association of Changing Hospital Readmission Rates With Mortality Rates After Hospital Discharge. JAMA. 2017;318(3):270–278.

[10] Hofstede SN, van Bodegom-Vos L, Kringos DS, et al. Mortality, readmission and length of stay have different relationships using hospital-level versus patient-level data: an example of the ecological fallacy affecting hospital performance indicators. BMJ Quality & Safety 2018;27:474-483.

For peer review only

[11] Auger, K. A., Teufel, R. J., Harris, J. M., Gay, J. C., Del Beccaro, M. A., Neuman, M. I., ... Shah, S. S. (2017). Children's hospital characteristics and readmission metrics. *Pediatrics*, *139*(2).

[12] Paterson JM, Williams JI, Kreder HJ, et al. Provider volumes and early outcomes of primary total joint replacement in Ontario. Can J Surg. 2010;53(3):175–183.

[13] Canadian Institute for Health Information. Your Health System – In Depth [internet]. Ottawa: CIHI, 2019 https://yourhealthsystem.ca (accessed 18 November 2019).

[14] Canadian Institute for Health Information. Indicator Library [internet]. Ottawa: CIHI; 2019 https://www.cihi.ca/en/indicator-library (accessed 18 November 2019).

[15] Canadian Institute for Health Information. Your Health System: In Depth. Technical Notes for Contextual Measures (October 2019). Ottawa: CIHI; 2019.

[16] Canadian Institute for Health Information. Model specifications — clinical indicators. 2019. Ottawa: CIHI, 2019.

[17] Canadian Institute for Health Information. Hospital Standardized Mortality Ratio - Technical Notes, September 2019. Ottawa, ON: CIHI; 2019.

[18] Canadian Institute for Health Information. Indicator Library: Peer Group Methodology. Ottawa: CIHI; 2016.

[19] Canadian Institute for Health Information. HSMR: Frequently asked questions [internet]. Ottawa: CIHI; 2020 https://www.cihi.ca/en/hospital-standardized-mortality-ratio-hsmr-frequently-asked-questions#_faq22 (accessed 13 April 2020).

[20] BMJ. 11. Correlation and regression [internet]. London; BMJ; 2020 https://www.bmj.com/about-bmj/resources-readers/publications/statistics-square-one/11-correlation-and-regression (accessed 13 April 2020).

[21] Fekri O, Manukyan E, Klazinga N. Appropriateness, effectiveness, and safety of care delivered in Canadian hospitals: a longitudinal assessment on the utility of publicly reported performance trend data between 2012-2013 and 2016-2017. BMJ Open 2020; **O**:e035447. Doi:10.1136/bmjopen-2019-035447

[22] Canadian Institute for Health Information. Indicator Library: General Methodology Notes – Clinical Indicators May 2020. Ottawa. CIHI; 2020.

[23] Martin M, Davies H. Public release of information on quality of care: how are health services and the public expected to respond?, J Health Serv Res Policy, 2001, vol. 6 (pg. 158-62)

For peer review only

[24] Smith, P. (1995) On the Unintended Consequences of Publishing Performance Data in the Public Sector. International Journal of Public Administration, 18, 277-310.

[25] Canadian Institute for Health Information. A Performance Measurement Framework for Canadian Hospitals, 2013. Ottawa: CIHI; 2013.

[26] Howlett, K. Health-care providers unite in bid to improve care, cut costs. 2011; https://www.theglobeandmail.com/news/toronto/health-care-providers-unite-in-bid-to-improve-care-cut-costs/article575685/ (accessed 23 December 2019).

[27] Huynh TM, Baker GR, Bierman A, et al. Exploring accountable care in Canada: integrating financial and quality incentives for physicians and hospitals. Ottawa: Canadian Foundation for Healthcare Improvement; 2014.

[28] Hibbard, J. H., Greene, J., & Daniel, D. (2010). What Is Quality Anyway? Performance Reports That Clearly Communicate to Consumers the Meaning of Quality of Care. Medical Care Research and Review, 67(3), 275–293.

[29] Canadian Institute for Health Information. A performance measurement framework for the Canadian health system. Ottawa: Canadian Institute for Health Information; 2013.

For peer review only

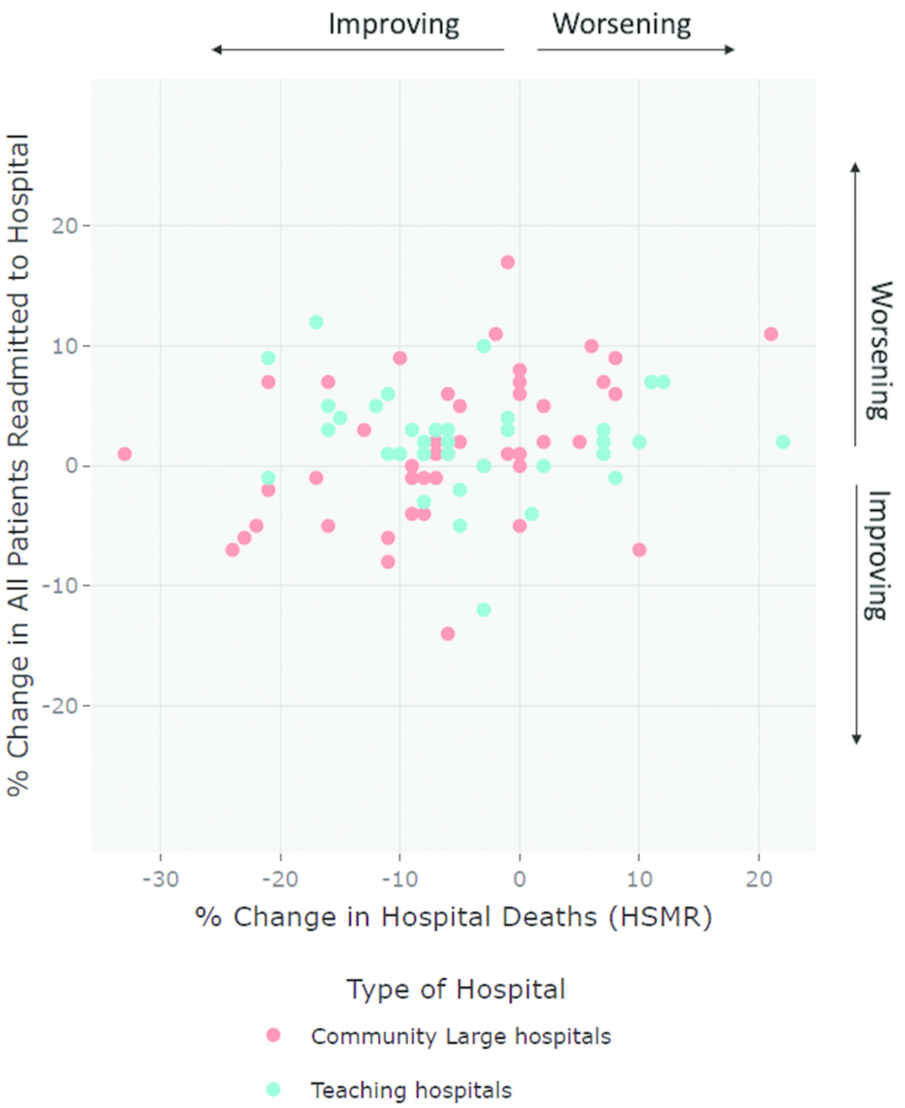

Figure 1 Scatterplot of percent change between 2013-14 – 2017-18 for Readmission and HSMR (by hospital peer-group)

87x103mm (300 x 300 DPI)

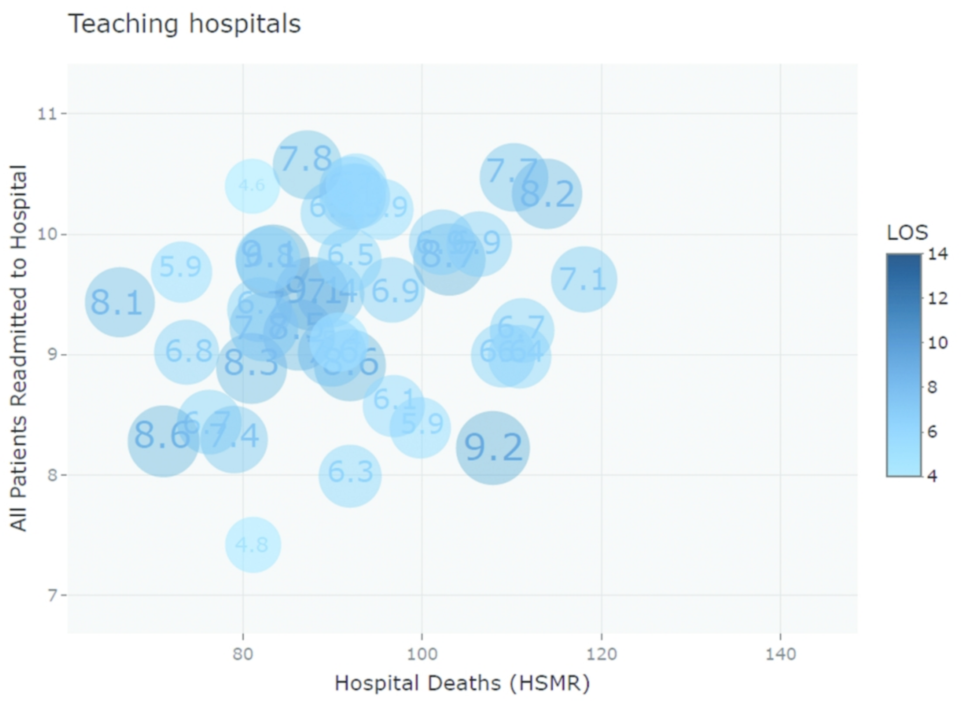

Figure 2 Scatterplot of Teaching hospital values for Hospital Deaths (HSMR), Readmission and LOS (2017-18)

119x90mm (300 x 300 DPI)

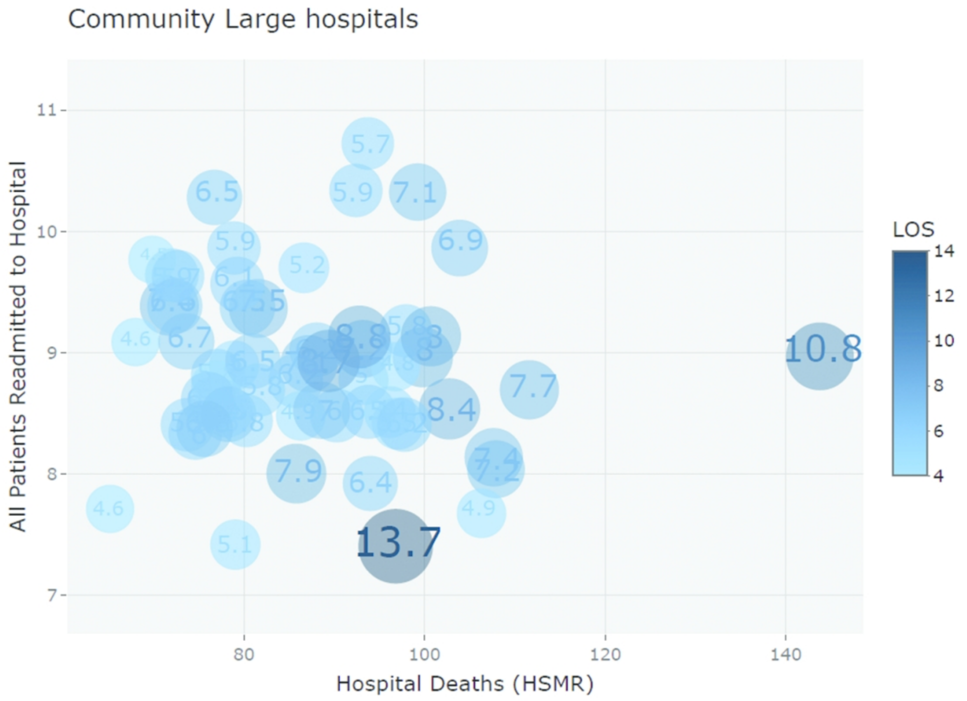

Figure 3 Scatterplot of Community-large hospital values for Hospital Deaths (HSMR), Readmission and LOS (2017-18)

119x90mm (300 x 300 DPI)

Supplementary file

Provincial/territorial range of % change difference (2013-14 vs. 2017-18), mean % change (and 95% Confidence Intervals), combined Teaching and Community-Large hospitals

Province/territory	Indicator	Range of % change (2013-14 vs. 2017-18)	Mean % change (95% CI)
Alberta	All Patients Readmitted to Hospital	-6 to 17	3.1 (-0.7 to 6.9)
	Hospital Deaths (HSMR)	-21 to 22	-0.7 (-9.3 to 7.9)
British Columbia	All Patients Readmitted to Hospital	-12 to 12	1.9 (-1.2 to 5)
	Hospital Deaths (HSMR)	-33 to 11	-6.5 (-11.4 to -1.6)
Manitoba	All Patients Readmitted to Hospital	3 to 10	6.7 (-2.1 to 15.4)
	Hospital Deaths (HSMR)	-13 to 12	-1.3 (-32.6 to 29.9)
New Brunswick	All Patients Readmitted to Hospital	-8 to 2	-3.2 (-11.2 to 4.7)
	Hospital Deaths (HSMR)	-11 to 10	-2.5 (-16.8 to 11.8)
Newfoundland and Labrador	All Patients Readmitted to Hospital	1 to 10	5.5 (-51.7 to 62.7)
	Hospital Deaths (HSMR)	-6 to 6	0.0 (-76.2 to 76.2)
Nova Scotia	All Patients Readmitted to Hospital	-4 to 11	3.5 (-91.8 to 98.8)
	Hospital Deaths (HSMR)	1 to 21	11.0 (-116.1 to 138.1)
Ontario	All Patients Readmitted to Hospital	-14 to 9	0.9 (-1 to 2.8)
	Hospital Deaths (HSMR)	-24 to 8	-5.8 (-9.2 to -2.5)
Prince Edward Island*	All Patients Readmitted to Hospital	-5 to -5	N/A
	Hospital Deaths (HSMR)	-22 to -22	N/A
Quebec	All Patients Readmitted to Hospital	1 to 9	4.8 (1.8 to 7.8)
	Hospital Deaths (HSMR)	-21 to -1	-12.0 (-19.5 to -4.5)
Saskatchewan	All Patients Readmitted to Hospital	-2 to 3	0.8 (-2.5 to 4)
	Hospital Deaths (HSMR)	-11 to -5	-7.8 (-11.7 to -3.8)

*Only one hospital value.

For peer review only

Subset of hospitals (n=81), with both Readmission and Hospital Deaths (HSMR) values, used in performance trends over time analysis

Provincial/territorial jurisdiction	Community — large hospitals	Teaching hospitals	Jurisdiction total
Alberta	4	7	11
British Columbia	11	6	17
Manitoba	1	2	3
New Brunswick	3	1	4
Newfoundland and Labrador	1	1	2
Nova Scotia	1	1	2
Ontario	21	10	31
Prince Edward Island	1	0	1
Quebec	2	4	6
Saskatchewan	0	4	4
**Total**	**45**	**36**	**81**

Facility characteristic averages by hospital peer-groups

Facility characteristic	Unit	Mean value, (n of hospitals)	
		Teaching hospitals	Community – Large hospitals
**Number of Acute Care Hospital Stays**	# of days	27,322 (n=53)	20,421 (n=66)
**Number of Acute Care Beds**	# of beds	474 (n=53)	328 (n=66)
**Number of Emergency Department Visits**	# of visits	83,441 (n=40)	86,962 (n=43)
**Average Acute Care Resource Intensity Weight (RIW)**	average RIW	1.6 (n=53)	1.2 (n=66)
**Total Acute Care RIW**	total RIW	43,295 (n=53)	25,057 (n=66)
**Hospital Occupancy Rate**	% of occupancy	88.9 (n=44)	89.9 (n=61)
**Patients Admitted Through the Emergency Department (%)**	% of patients	44.4 (n=53)	54.4 (n=66)
**Patient Days in Alternate Level of Care (Percentage)**	%	11.4 (n=43)	15.4 (n=53)

For peer review only

Correlation matrix (scatterplot) of both Teaching and Community-Large hospitals

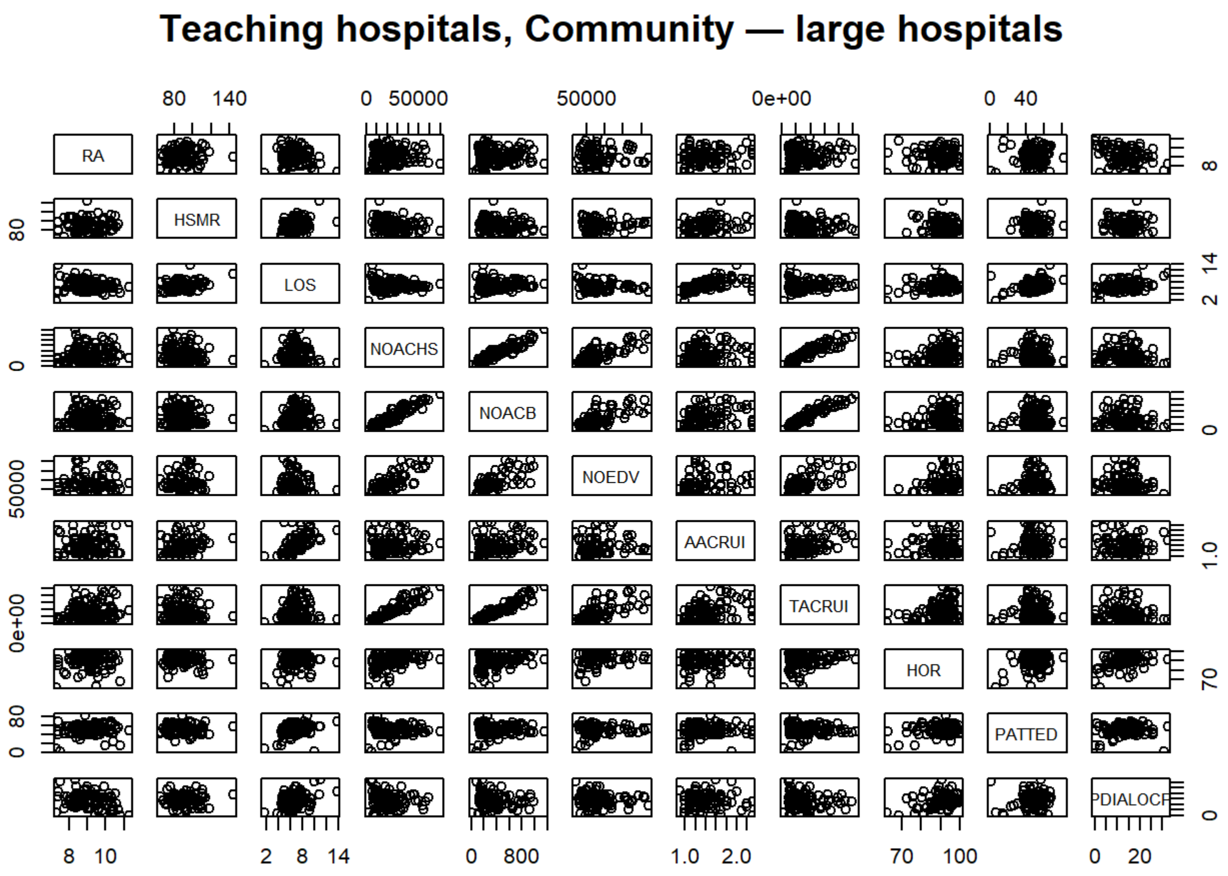

Indicator acronyms: All Patients Readmitted to Hospital (RA); Hospital Deaths (HSMR); Average Length of Stay (LOS); Number of Acute Care Hospital Stays (NOACHS); Number of Acute Care Beds (NOACB); Number of Emergency Department Visits (NOEDV); Average Acute Care Resource Intensity Weight (AACRUI); Total Acute Care Resource Intensity Weight (TACRUI); Hospital Occupancy Rate (HOR); Patients Admitted Through the Emergency Department (%) (PATTED); Patient Days in Alternate Level of Care (Percentage) (PDIALOCP).

Correlation matrix (scatterplot) of Teaching hospitals

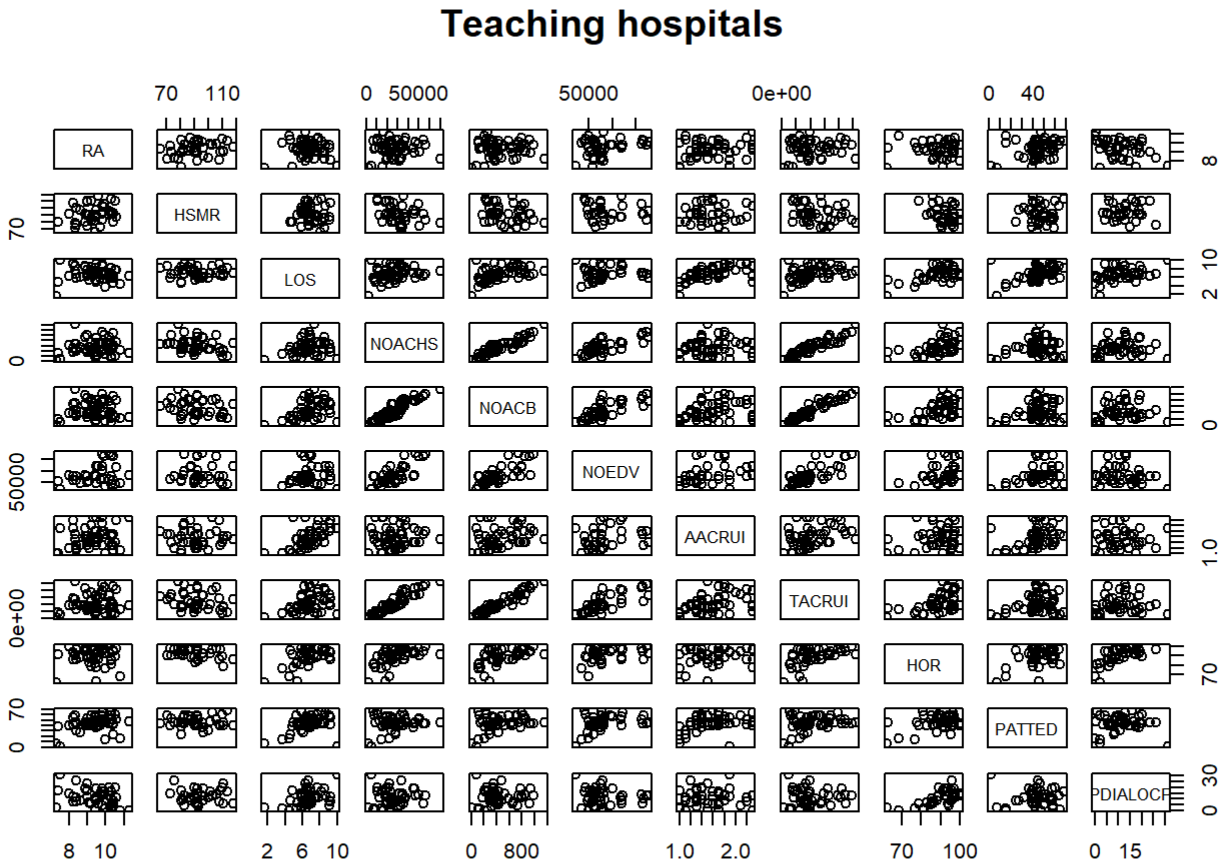

Indicator acronyms: All Patients Readmitted to Hospital (RA); Hospital Deaths (HSMR); Average Length of Stay (LOS); Number of Acute Care Hospital Stays (NOACHS); Number of Acute Care Beds (NOACB); Number of Emergency Department Visits (NOEDV); Average Acute Care Resource Intensity Weight (AACRUI); Total Acute Care Resource Intensity Weight (TACRUI); Hospital Occupancy Rate (HOR); Patients Admitted Through the Emergency Department (%) (PATTED); Patient Days in Alternate Level of Care (Percentage) (PDIALOCP).

Correlation matrix (scatterplot) of Community-Large hospitals

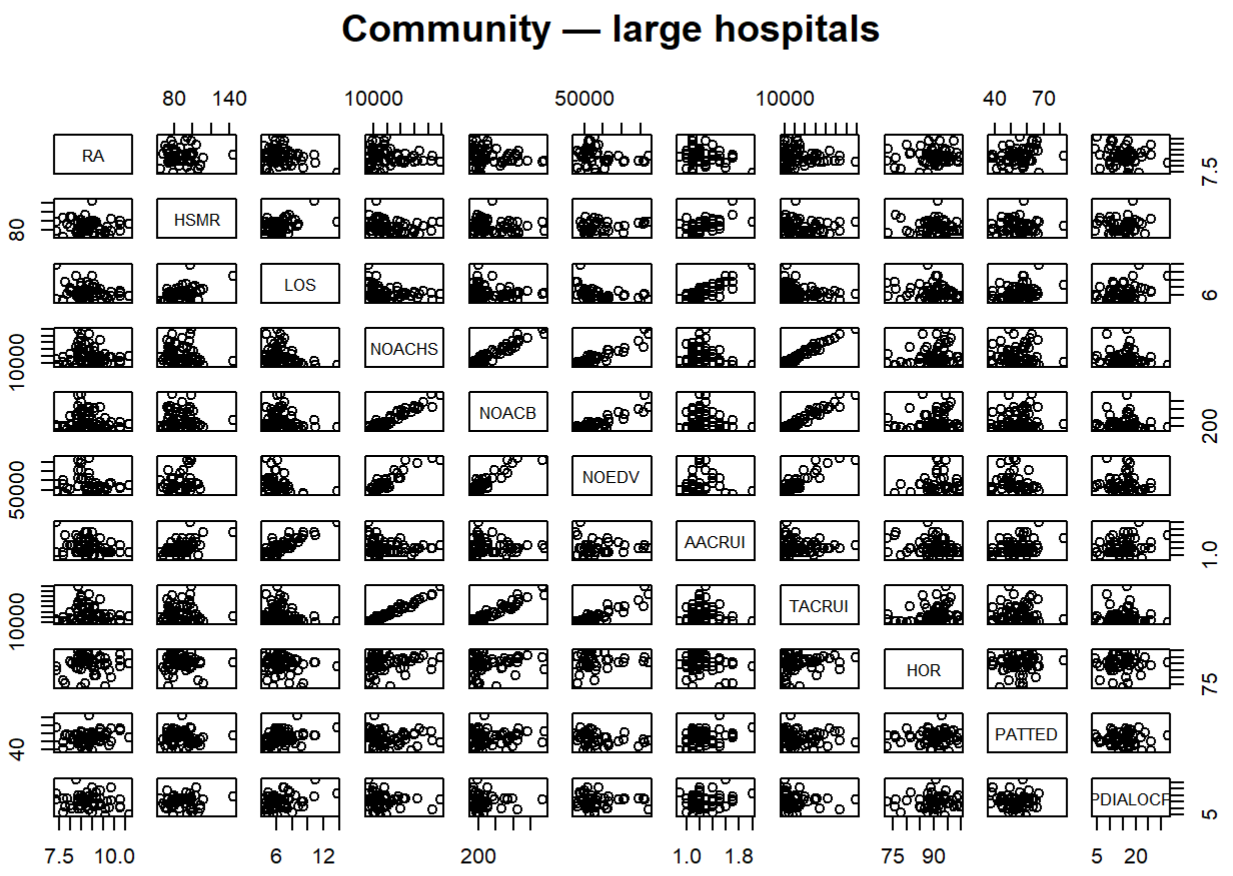

Indicator acronyms: All Patients Readmitted to Hospital (RA); Hospital Deaths (HSMR); Average Length of Stay (LOS); Number of Acute Care Hospital Stays (NOACHS); Number of Acute Care Beds (NOACB); Number of Emergency Department Visits (NOEDV); Average Acute Care Resource Intensity Weight (AACRUI); Total Acute Care Resource Intensity Weight (TACRUI); Hospital Occupancy Rate (HOR); Patients Admitted Through the Emergency Department (%) (PATTED); Patient Days in Alternate Level of Care (Percentage) (PDIALOCP).

The RECORD statement – checklist of items, extended from the STROBE statement, that should be reported in observational studies using routinely collected health data.

	Item No.	STROBE items	Location in manuscript where items are reported	RECORD items	Location in manuscript where items are reported
**Title and abstract**					
	1	(a) Indicate the study's design with a commonly used term in the title or the abstract (b) Provide in the abstract an informative and balanced summary of what was done and what was found		RECORD 1.1: The type of data used should be specified in the title or abstract. When possible, the name of the databases used should be included.<br><br>RECORD 1.2: If applicable, the geographic region and timeframe within which the study took place should be reported in the title or abstract.<br><br>RECORD 1.3: If linkage between databases was conducted for the study, this should be clearly stated in the title or abstract.	1.1 Noted in title and abstract.<br><br>1.2 Noted in title and abstract.<br><br>1.3. Not applicable as no linkages were performed.
**Introduction**					
Background rationale	2	Explain the scientific background and rationale for the investigation being reported			Introduction paragraphs 1-4
Objectives	3	State specific objectives, including any prespecified hypotheses			Introduction paragraph 4
**Methods**					
Study Design	4	Present key elements of study design early in the paper			Methods section
Setting	5	Describe the setting, locations, and relevant dates, including periods of recruitment, exposure, follow-up, and data collection			Methods paragraphs 1-12
Participants	6	*(a) Cohort study* - Give the eligibility criteria, and the sources and methods of selection of participants. Describe methods of follow-up<br>*Case-control study* - Give the eligibility criteria, and the sources and methods of case ascertainment and control selection. Give the rationale for the choice of cases and controls<br>*Cross-sectional study* - Give the eligibility criteria, and the sources and methods of selection of participants<br><br>*(b) Cohort study* - For matched studies, give matching criteria and number of exposed and unexposed<br>*Case-control study* - For matched studies, give matching criteria and the number of controls per case		RECORD 6.1: The methods of study population selection (such as codes or algorithms used to identify subjects) should be listed in detail. If this is not possible, an explanation should be provided.<br><br>RECORD 6.2: Any validation studies of the codes or algorithms used to select the population should be referenced. If validation was conducted for this study and not published elsewhere, detailed methods and results should be provided.<br><br>RECORD 6.3: If the study involved linkage of databases, consider use of a flow diagram or other graphical display to demonstrate the data linkage process, including the number of individuals with linked data at each stage.	6.1 N/A<br><br>6.2 N/A<br><br>6.3 N/A
Variables	7	Clearly define all outcomes, exposures, predictors, potential confounders, and effect modifiers. Give diagnostic criteria, if applicable.		RECORD 7.1: A complete list of codes and algorithms used to classify exposures, outcomes, confounders, and effect modifiers should be provided. If these cannot be reported, an explanation should be provided.	7.1 Outcomes and variables described in the Methods section paragraphs 1-7, 9, 10
Data sources/ measurement	8	For each variable of interest, give sources of data and details of methods of assessment (measurement).<br>Describe comparability of assessment methods if there is more than one group			Data source described in Methods paragraph 1

For peer review only

Bias	9	Describe any efforts to address potential sources of bias			Bias of available data described in Methods paragraph 4-6
Study size	10	Explain how the study size was arrived at			Methods paragraph 5
Quantitative variables	11	Explain how quantitative variables were handled in the analyses. If applicable, describe which groupings were chosen, and why			Groupings described in Methods paragraphs 5-7<br><br>Quantitative variables described in Methods paragraphs 8-11
Statistical methods	12	(a) Describe all statistical methods, including those used to control for confounding<br>(b) Describe any methods used to examine subgroups and interactions<br>(c) Explain how missing data were addressed<br>(d) *Cohort study* - If applicable, explain how loss to follow-up was addressed<br>*Case-control study* - If applicable, explain how matching of cases and controls was addressed<br>*Cross-sectional study* - If applicable, describe analytical methods taking account of sampling strategy<br>(e) Describe any sensitivity analyses			Methods paragraphs 8-12
Data access and cleaning methods		..		RECORD 12.1: Authors should describe the extent to which the investigators had access to the database population used to create the study population.<br><br>RECORD 12.2: Authors should provide information on the data cleaning methods used in the study.	12.1 Noted in methods section that data is publicly available for use. Also described in Data Availability Statement at conclusion of manuscript.<br><br>12.2 No data cleaning methods were used in the study.
Linkage		..		RECORD 12.3: State whether the study included person-level, institutional-level, or other data linkage across two or more databases. The methods of linkage and methods of linkage quality evaluation should be provided.	12.3 No data linkage was performed.
**Results**					
Participants	13	(a) Report the numbers of individuals at each stage of the study (*e.g.*, numbers potentially eligible, examined for eligibility, confirmed eligible, included in the study, completing follow-up, and analysed)<br>(b) Give reasons for non-participation at each stage.<br>(c) Consider use of a flow diagram		RECORD 13.1: Describe in detail the selection of the persons included in the study (*i.e.,* study population selection) including filtering based on data quality, data availability and linkage. The selection of included persons can be described in the text and/or by means of the study flow diagram.	13.1 No person-level data was used in the study. Number of hospitals included in study described in Methods paragraph 7, and Results section Table 1, and supplementary file
Descriptive data	14	(a) Give characteristics of study participants (*e.g.*, demographic, clinical, social) and information on exposures and potential confounders<br>(b) Indicate the number of participants with missing data for each variable of interest<br>(c) *Cohort study* - summarise follow-up time (*e.g.*, average and total amount)			Descriptive information on hospitals are stated in Methods section, and in Table 1 of Results section.

For peer review only

Outcome data	15	*Cohort study* - Report numbers of outcome events or summary measures over time<br>*Case-control study* - Report numbers in each exposure category, or summary measures of exposure<br>*Cross-sectional study* - Report numbers of outcome events or summary measures			Reported in Table 2 of Results section.
Main results	16	(a) Give unadjusted estimates and, if applicable, confounder-adjusted estimates and their precision (e.g., 95% confidence interval). Make clear which confounders were adjusted for and why they were included<br>(b) Report category boundaries when continuous variables were categorized<br>(c) If relevant, consider translating estimates of relative risk into absolute risk for a meaningful time period			The Results section contains three main headings (corresponding to research questions 1,2,3, with the 4th addressed concurrently).
Other analyses	17	Report other analyses done—e.g., analyses of subgroups and interactions, and sensitivity analyses			Subgroup analyses by hospital type/size are described throughout Results section, notably tables 1,2,3 & figures 1,2,3.<br><br>Jurisdictional and hospital type/size breakdowns provided in supplementary file.
**Discussion**					
Key results	18	Summarise key results with reference to study objectives			Discussion paragraphs 2-3<br>Conclusion paragraph 1
Limitations	19	Discuss limitations of the study, taking into account sources of potential bias or imprecision. Discuss both direction and magnitude of any potential bias		RECORD 19.1: Discuss the implications of using data that were not created or collected to answer the specific research question(s). Include discussion of misclassification bias, unmeasured confounding, missing data, and changing eligibility over time, as they pertain to the study being reported.	19.1 Noted under paragraphs 2-4 of Strengths & Limitations section of Discussion section.
Interpretation	20	Give a cautious overall interpretation of results considering objectives, limitations, multiplicity of analyses, results from similar studies, and other relevant evidence			Paragraphs 2-3 of Discussion section.
Generalisability	21	Discuss the generalisability (external validity) of the study results			Paragraph 5 of Discussion section.
**Other Information**					
Funding	22	Give the source of funding and the role of the funders for the present study and, if applicable, for the original study on which the present article is based			Funding statement
Accessibility of protocol, raw data, and programming code		..		RECORD 22.1: Authors should provide information on how to access any supplemental information such as the study protocol, raw data, or programming code.	22.1 Noted in Data Availability Statement, and cited in Methods section.

For peer review only

*Reference: Benchimol EI, Smeeth L, Guttmann A, Harron K, Moher D, Petersen I, Sørensen HT, von Elm E, Langan SM, the RECORD Working Committee. The REporting of studies Conducted using Observational Routinely-collected health Data (RECORD) Statement. *PLoS Medicine* 2015; in press.

*Checklist is protected under Creative Commons Attribution (CC BY) license.

For peer review only

BMJ Open

Associations between Hospital Deaths (HSMR), Readmission and Length of Stay (LOS): a longitudinal assessment of performance results and facility characteristics of teaching and large-sized hospitals in Canada between 2013-14 to 2017-18

Journal:	*BMJ Open*
Manuscript ID	bmjopen-2020-041648.R2
Article Type:	Original research
Date Submitted by the Author:	14-Dec-2020
Complete List of Authors:	Fekri, Omid; University of Amsterdam, Academic Medical Center<br>Fekri, Omid; Academic Medical Center,<br>Manukyan, Edgar; University of Amsterdam, Academic Medical Center<br>Klazinga, Niek; University of Amsterdam, Academic Medical Center
**Primary Subject Heading**:	Health services research
Secondary Subject Heading:	Public health, Epidemiology, Health policy, Health services research
Keywords:	Quality in health care < HEALTH SERVICES ADMINISTRATION & MANAGEMENT, Organisation of health services < HEALTH SERVICES ADMINISTRATION & MANAGEMENT, Clinical governance < HEALTH SERVICES ADMINISTRATION & MANAGEMENT

SCHOLARONE™ Manuscripts

I, the Submitting Author has the right to grant and does grant on behalf of all authors of the Work (as defined in the below author licence), an exclusive licence and/or a non-exclusive licence for contributions from authors who are: i) UK Crown employees; ii) where BMJ has agreed a CC-BY licence shall apply, and/or iii) in accordance with the terms applicable for US Federal Government officers or employees acting as part of their official duties; on a worldwide, perpetual, irrevocable, royalty-free basis to BMJ Publishing Group Ltd ("BMJ") its licensees and where the relevant Journal is co-owned by BMJ to the co-owners of the Journal, to publish the Work in this journal and any other BMJ products and to exploit all rights, as set out in our licence.

The Submitting Author accepts and understands that any supply made under these terms is made by BMJ to the Submitting Author unless you are acting as an employee on behalf of your employer or a postgraduate student of an affiliated institution which is paying any applicable article publishing charge ("APC") for Open Access articles. Where the Submitting Author wishes to make the Work available on an Open Access basis (and intends to pay the relevant APC), the terms of reuse of such Open Access shall be governed by a Creative Commons licence – details of these licences and which Creative Commons licence will apply to this Work are set out in our licence referred to above.

Other than as permitted in any relevant BMJ Author's Self Archiving Policies, I confirm this Work has not been accepted for publication elsewhere, is not being considered for publication elsewhere and does not duplicate material already published. I confirm all authors consent to publication of this Work and authorise the granting of this licence.

For peer review only

Title page

Date: 12 December 2020

Manuscript title:

Associations between Hospital Deaths (HSMR), Readmission and Length of Stay (LOS): a longitudinal assessment of performance results and facility characteristics of teaching and large-sized hospitals in Canada between 2013-14 to 2017-18

Authors:

Omid Fekri (corresponding author: o.fekri@amsterdamumc.nl)

Edgar Manukyan

Niek Klazinga

Affiliation: University Medical Centers - University of Amsterdam

Word count: 4977 (excluding tables, graphs, abstract, references)

For peer review only

Associations between Hospital Deaths (HSMR), Readmission and Length of Stay (LOS): a longitudinal assessment of performance results and facility characteristics of teaching and large-sized hospitals in Canada between 2013-14 to 2017-18

Abstract

Objectives: To examine the association between Hospital Deaths (HSMR), Readmission, Length of Stay (LOS), and eight hospital characteristics.

Design: Longitudinal observational study.

Setting: A total of 119 teaching and large-sized hospitals in Canada between fiscal years 2013–14 and 2017-18.

Participants: Analysis focused on indicator results and characteristics of individual Canadian hospitals.

Primary and secondary outcomes: Hospital Deaths (HSMR); All Patients Readmitted to Hospital; Average Length of Stay (LOS); and a series of eight hospital characteristic summary measures: Number of Acute Care Hospital Stays; Number of Acute Care Beds; Number of Emergency Department Visits; Average Acute Care Resource Intensity Weight; Total Acute Care Resource Intensity Weight; Hospital Occupancy Rate; Patients Admitted Through the Emergency Department (%); Patient Days in Alternate Level of Care (%).

Results: Comparing 2013-14 to 2017-18, Hospital deaths (HSMR) largely declined, while readmissions increased; 69% of hospitals decreased their hospital deaths (HSMR), while 65% of hospitals increased their readmissions rates. A greater proportion of Community-Large hospitals (31%, n=14) improved on both hospital deaths (HSMR) and readmission compared to Teaching hospitals (13.9%, n=5). Hospital deaths (HSMR), readmission and LOS largely showed very weak and non-significant correlations. LOS was largely positively and statistically significantly correlated with the suite of eight hospital characteristics. Hospital deaths (HSMR) was largely negatively (not statistically significantly) correlated with the hospital characteristics. Readmission was largely not statistically significantly correlated and showed no clear pattern of correlation (direction) with hospital characteristics.

Conclusions: Examining publicly-reported hospital performance results can reveal meaningful insights into the association among outcome indicators and hospital characteristics. Good or bad hospital performance in one care domain does not necessarily reflect similar performance in other care domains. Thus, caution is warranted in a narrow use of outcome indicators in the design and operationalization of hospital performance measurement and governance models (namely pay-for-performance schemes). Analysis such as this can also inform quality-improvement strategies and targeted efforts to address domains of care experiencing declining performance over time; further granular subdivision of the analyses, for example by hospital peer-groups, can reveal notable differences in performance.

Article Summary - Strengths and limitations of this study

- Assessed correlations across eight hospital characteristics and three hospital performance indicators.
- Assessed five years of performance data.

For peer review only

- Examined the majority of Teaching and Community-Large hospitals in Canada.
- Inability to apply more complex statistical modelling techniques due to limitations on the use of aggregate hospital-level data in secondary analyses.
- LOS is an aggregate of all hospitalizations, and could not be restricted to condition-specific cases (of hospital death or readmission).

For peer review only

Introduction

Over the last two decades, there has been substantial interest in hospital performance[1], and with financing of hospitals increasingly tied to improving the quality of care delivered[2]. Along with improving the quality of care, a tandem goal of hospital reforms has been to improve efficiency[3] (i.e., reducing waste, streamlining care pathways, increasing patient throughput, optimizing the use of technology, etc.). Hospital deaths[4] and readmission to hospital[5] are among the most commonly used indicators to measure quality of hospital care, while average Length of Stay (LOS) is often used as a measure of efficiency[6]. The three measures together (hospital deaths, readmission and LOS) have been the subject of increased interest in recent years to assist with more reliable interpretations of hospital performance[7].

However, the goals of achieving quality and efficiency can at times be opposing. For example, it seems warranted to investigate whether a hastened hospital stay (shorter LOS) would lead to an increased chance of readmission to hospital[8]. Similarly, do efforts to reduce hospital readmissions have the unintended consequence of increasing the likelihood of mortality after hospitalization[9]? While hospital deaths and readmission are both desired to be reduced, it is not definite (and varying across diseases and clinical procedures) whether a patient's LOS should be lower or higher in order to minimize readmission or in-hospital mortality. However, what can be deduced is that the relationships between LOS, in-hospital mortality and readmission are intertwined and interdependent. Hence governance of hospitals based on these publicly reported indicators should be based on acknowledgment and consideration of these interdependencies.

Yet, despite a sizeable research community investigating the interrelationship between these indicators, the evidence-base on the patterns of these interdependencies remains inconclusive due to wide heterogeneity in methods and findings across studies (which speaks to the complexity of the topic). For example, a switch between the unit of analysis (from patient-level to hospital-level), on the same underlying admissions data, will yield inconsistent, and even inverse, results[10]. In recent years, researchers have also examined hospital characteristics, such as hospital volumes[11] or hospital teaching status[12] to better understand any associations between LOS, readmission and in-hospital mortality.

Much of the afore cited literature originates from the United States and Europe. With a scarcity of local examples, this study used a large, nationally-representative dataset of hospital performance measures (produced by the Canadian Institute for Health Information (CIHI)) to expand interest and add evidence for the Canadian context. Specifically, we investigate the relationship between hospital deaths, readmission and LOS, and explore any associations with hospital characteristics. Our specific research questions are:

1. How have hospitals performed in both the hospital deaths (HSMR) and readmission indicators over time?
2. What is the correlation between hospital deaths (HSMR), readmissions and LOS?
3. How do a series of eight hospital characteristics correlate with hospital deaths (HSMR), readmissions and LOS?
4. Do the results of the aforementioned research questions show differences between peer groups of Teaching hospitals and Community-Large hospitals?

For peer review only

Methods

Data

We used the all data export report file from CIHI's Your Health System In Depth online tool[13] to perform the analyses. The data file contains results per hospital for all indicators published on the online tool as well as contextual measures and additional variables to assist with analysis and interpretation. Five singleton fiscal year (1 April to 31 March) data points were available covering 2013–14 to 2017–18 for the indicators capturing Hospital Deaths (HSMR) and All Patients Readmitted to Hospital (henceforth referred to 'Readmission'), while LOS and eight hospital characteristics measures were only available for the most recent year (2017-18).

Definition of variables

The following indicators were used for the analysis: Hospital Deaths (HSMR) (Hospital Standardized Mortality Ratio), Readmission (%), and LOS (days); and eight contextual measures of hospital facility characteristics: Number of Acute Care Hospital Stays; Number of Acute Care Beds; Number of Emergency Department Visits; Average Acute Care Resource Intensity Weight (RIW); Total Acute Care RIW; Hospital Occupancy Rate; Patients Admitted Through the Emergency Department; Patient Days in Alternate Level of Care (%).

HSMR (hospital standardized mortality ratio) and other variations of summary hospital mortality measures are commonly-used indicators to assess hospital performance. The Hospital Deaths (HSMR) indicator is a ratio of observed to expected in-hospital mortality, capturing the 72 leading causes of hospital death (representing ~80% of all in-hospital mortality). The Readmission indicator captures all urgent patient readmissions within 30-days. The average LOS indicator is a sum of all valid days spent in hospital, divided by the total number of inpatient cases. Detailed technical notes on these indicators[14], and on hospital facility characteristics[15], are made available by CIHI through its Indicator Library.

Both hospital deaths (HSMR) and readmission indicators are risk-adjusted. Hospital deaths (HSMR) risk-adjustment variables are: age, sex, LOS, admission category, comorbidity (Charlson Index Score), and transfers. As the Readmission indicator is an aggregate of four sub-categories of readmission (medical, surgical, obstetric, paediatric), the Readmission risk-adjustment variables are not constant across the four sub-categories; this range of risk-adjustment variables are: age, sex, acute care hospitalizations in previous 6 months, admission category, comorbidity (Charlson Index Score), and case-mix groupings. Detailed information on model specifications and coefficients used in calculations are available elsewhere[16, 17].

CIHI classifies the approximately 600 hospitals in Canada into four distinct peer-group types: Teaching hospitals; Community—Large hospitals; Community—Medium hospitals; and Community—Small hospitals. This classification facilitates meaningful comparisons across hospitals of similar structural characteristics, patient volume, and clinical complexity[18]. Since characteristics of hospitals are not included in risk-adjustment models, any comparison of two or more hospitals' individual performance should be done within their respective hospital peer-groups.

A hospital is designated as 'Teaching' by provincial/territorial ministries of health, or was identified as such in the provincial/territorial ministry's submission to CIHI's Management Information System (MIS) Database. Community—Large hospitals meet two of the following three criteria: more than 8,000 inpatient cases; more than 10,000 weighted cases; or more than 50,000 inpatient days.

In order to qualify for public-reporting of results for the Hospital Deaths (HSMR) indicator, a hospital must meet a minimum of 2,500 eligible Hospital Deaths (HSMR) cases for each of the most recent three consecutive

For peer review only - http://bmjopen.bmj.com/site/about/guidelines.xhtml

years[19]. Consequently, no Community—Small hospitals met this criteria to have publicly-reported Hospital deaths (HSMR) results. Of the 93 Community—Medium hospitals only 11 hospitals met the minimum reporting requirements and had Hospital deaths (HSMR) results reported. Since this represents only 8.5% of the entire peer-group, it was decided to also exclude Community—Medium hospitals, alongside Community—Small hospitals, in this analysis. Hospitals with only one year of data available, for both Readmission and Hospital Deaths (HSMR) indicators, for either 2013-14 or 2017-18 only, were excluded from performance trend analysis. Therefore, a total of 119 hospitals were included in the overall study, 53 Teaching hospitals and 66 Community-Large hospitals (representing 67.9% and 68.2% of all hospitals in their respective peer-group totals in the available online dataset). A subset of 81 hospitals were included in the performance trend analysis.

__Statistical analyses__

Descriptive statistics for the analysis of LOS, Hospital Deaths (HSMR) and Readmission indicators are presented by range of values, peer-group means and 95% confidence intervals (CI), and coefficient of variation (CoV) (see Table 1). Trend over time is calculated as the percent-change difference between first and last year of data (2013-14 and 2017-18). A paired-t test was used to determine whether absolute changes in rates between 2013-14 and 2017-18 were significant.

To compare indicator rates per hospital across 2013-14 to 2017-18, three possible outcomes are inferred: a decrease in rate (2013-14 > 2017-18); an increase in rate (2013-14 < 2017-18); and no change in rate (2013-14 = 2017-18). Multiplying these three outcomes by the two indicators of interest (Hospital Deaths (HSMR) and Readmission), in tandem, yields a total of nine trend outcomes (see Table 2).

Graphical representation of the aforementioned tests are shown via scatterplots depicting: 1) percent-change over time for Hospital Deaths (HSMR) and Readmission (delineated by peer-group) (see Figure 1); and 2) 2017-18 data year results on Hospital Deaths (HSMR) and Readmission, with LOS depicted as the size of the bubble plot (see Figures 2 & 3).

A Spearman's Rank Correlation test examines the association between LOS, Hospital Deaths (HSMR) and Readmission on 2017-18 data year values (with breakdowns for Teaching and Community—Large hospital peer-groups). Strengths of correlations, the absolute value of R_s (positive and negative) are defined as: .00-.19 very weak; .20-.39 weak; .40-.59 moderate; .60-.79 strong; .80-1.0 very strong[20].

Lastly, a Spearman's Rank Correlation test was also used to assess the correlation between eight hospital facility characteristics against LOS, Hospital Deaths (HSMR) and Readmission values for 2017-18. All analyses were performed on R version 3.5.0 (R Foundation for Statistical Computing, Vienna, Austria).

__Patient and public involvement__

Patients or public were not involved in the design of this longitudinal, observational study. However, all data used are available in the public domain.

For peer review only

Results

Combined performance of hospital mortality (HSMR) and readmission over time

In comparing 2013-14 and 2017-18 indicator rates, Hospital deaths (HSMR) largely declined, while readmissions increased (see table 1). A paired-t test showed statistically significant changes in trend over time for both indicators: hospital deaths (HSMR) improved by a mean of -5.1 (95% CI -7.33 to -2.9, p<.001), and readmission rates increased by a mean of 0.15% (95% CI 0.04 to 0.26, p=.006). While not statistically significant, the Community-Large hospital peer-group showed a greater mean improvement in hospital deaths (HSMR) by -6.0% (95% CI -9.1 to -2.8), while Teaching hospitals improved by -4.1% (95% CI -7.5 to -0.8). Both hospital peer groups experienced a mean increase in readmission rates, with Community-Large hospitals at 1.6% (95% CI -0.3 to 3.4), and Teaching hospitals at 2.1% (95% CI 0.7 to 3.6). When examining the 2017-18 data year, Community-Large hospitals had a statistically significant lower rate of readmissions at 8.9 (95% CI, 8.7 to 9.1) compared to Teaching hospitals at 9.4 (95% CI, 9.2 to 9.6). Table 2 provides a lens on how individual hospitals performed in both indicators. Nine possible outcomes of performance are shown. Overall, 56 (69%) out of the total 81 hospitals assessed decreased their hospital deaths (HSMR), while only 23 (28%) hospitals decreasing their readmissions rates.

Figure 1 illustrates the combined percent change of hospital deaths (HSMR) and readmissions rates (comparing 2013-14 and 2017-18 individual hospital rates) delineated by hospital peer group. While coefficient of variation values are largely similar between the two peer groups for the two outcome indicators, nearly three times as many Community-Large hospitals (n=14) showed greater improvement in the bottom left quadrant of Figure 1 (decrease in both hospital deaths (HSMR) and readmission), than Teaching hospitals (n=5). These clear trends of overall decreasing hospital deaths and rising readmissions have been confirmed in our previous analysis[21].

For peer review only

Table 1 Descriptive statistics for combined analysis of hospital deaths (HSMR), readmission and LOS

	Teaching hospitals				Community-large hospitals			
**Number of hospitals, n**	36				45			
**Range of values for 2017-18 data year**	**Range of values**	**Teaching Peer-group mean* (95%CI)**	**Coefficient of variation, % (95%CI)**	**Median (IQR Q1–Q3)**	**Range of values**	**Community-large Peer-group mean* (95%CI)**	**Coefficient of variation, % (95%CI)**	**Median (IQR Q1–Q3)**
**LOS (days)**	4.6 to 9.2	7.1 (6.7 to 7.4)	16 (13 to 21)	6.9 (6.4–7.8)	4.5 to 13.7	6.5 (6.1 to 6.9)	24 (20 to 29)	6.2 (5.7–7.1)
**Hospital Deaths (HSMR)**	66 to 118	91.8 (87.8 to 95.7)	14 (11 to 18)	92 (82–100)	65 to 144	87.5 (83.9 to 91)	16 (13 to 19)	86 (77–96.5)
**Readmission (%)**	7.4 to 10.6	9.4 (9.2 to 9.6)	8 (7 to 11)	9.5 (9–9.9)	7.4 to 10.7	8.9 (8.7 to 9.1)	8 (7 to 10)	8.8 (8.5–9.63)
**Percent-change difference 2013-14 vs. 2017-18 (%)**	**Range of % change**	**Mean Teaching Peer-group % change* (95%CI)**			**Range of % change**	**Mean Community-large Peer-group % change* (95%CI)**		
**Hospital Deaths (HSMR)**	-21 to 22	-4.1 (-7.5 to -0.8)			-33 to 21	-6.0 (-9.1 to -2.8)		
**Readmission (%)**	-12 to 12	2.1 (0.7 to 3.6)			-14 to 17	1.6 (-0.3 to 3.4)		

*calculated by summing values of all hospitals within peer-group and dividing by number of hospitals

Table 2 Hospital outcomes on HSMR and Readmission changes over time

**Trend outcome**	**Hospital deaths (HSMR)**	**Readmission**	**Teaching hospitals (total n=36)** Number, (%)	**Community-Large hospitals (total n=45)** Number, (%)	**Total of all hospitals, number, (%)**
Decrease in both HSMR & Readmission	↓	↓	5 (13.9%)	14 (31.1%)	19 (23.5%)
Decrease in HSMR, increase in Readmission	↓	↑	20 (55.6%)	14 (31.1%)	34 (42.0%)
Decrease in HSMR, no change in Readmission	↓	=	1 (2.8%)	2 (4.4%)	3 (3.7%)
Increase in HSMR, decrease in Readmission	↑	↓	2 (5.6%)	1 (2.2%)	3 (3.7%)
Increase in both HSMR & Readmission	↑	↑	7 (19.4%)	8 (17.8%)	15 (18.5%)
Increase in HSMR, no change in Readmission	↑	=	1 (2.8%)	0	1 (1.2%)
No change in HSMR, decrease in Readmission	=	↓	0	1 (2.2%)	1 (1.2%)
No change in HSMR, increase in Readmission	=	↑	0	4 (8.9%)	4 (4.9%)
No change in both HSMR & Readmission	=	=	0	1 (2.2%)	1 (1.2%)

↑=signifies increasing rate; ↓=signifies decreasing rate; = signifies no change

For peer review only - http://bmjopen.bmj.com/site/about/guidelines.xhtml

Hospital deaths (HSMR), readmissions and LOS (2017-18)

In examining hospital deaths (HSMR), readmission and LOS for potential associations, only very weak to weak non-statistically significant results were observed (see table 3). The Community-Large hospital peer group showed greater variation in LOS values (CoV=24%, 95% CI 20 to 29) compared to the Teaching hospital peer group (CoV=16%, 95% CI 13 to 21). While not statistically significant, the Community-Large hospital peer group had a shorter mean LOS of 6.5 days (95% CI 6.1 to 6.9) compared to the Teaching hospital peer group of 7.1 days (95% CI 6.7 to 7.4) (see table 1). Figures 2 and 3 illustrate LOS, hospital deaths (HSMR) and readmission values for the 2017-18 data year (with LOS delineated in size and shading of bubble plot).

Table 3 Correlations between Hospital Deaths (HSMR), Readmission and LOS (breakdowns by Teaching and Community-Large hospitals) ***(2017-18)***

	LOS		Hospital deaths (HSMR)	
**Readmission**	Teaching:	**-0.04 (-0.41 to 0.33)**	Teaching:	**0.22 (-0.09 to 0.54)**
	Community-Large:	**0.04 (-0.23 to 0.31)**	Community-Large:	**-0.13 (-0.42 to 0.15)**

* p less than .01; ^ p less than .05; Direction of correlation is shown as Blue (positive) and Red (negative), and intensity of cell-colouring reflects strength of correlation. Correlation strength classification: .00-.19 very weak; .20-.39 weak; .40-.59 moderate; .60-.79 strong; .80-1.0 very strong.

For peer review only

Correlation between hospital characteristics, LOS, Hospital Deaths (HSMR) and Readmission

Table 4 Correlations between hospital characteristics on LOS, HSMR and Readmission ***(2017-18)***

**Hospital characteristic**	**Unit**		**Length of stay** Correlation coefficient (95% CI)	**Hospital deaths (HSMR)** Correlation coefficient (95% CI)	**Readmission** Correlation coefficient (95% CI)
**Number of Acute Care Hospital Stays**	# of days	All:	-0.04 (-0.24 to 0.16)	-0.14 (-0.34 to 0.05)	0.07 (-0.12 to 0.26)
		Teaching:	0.26 (-0.02 to 0.54)	-0.30 (-0.61 to 0.01)	0.07 (-0.23 to 0.37)
		Community-Large:	-0.36* (-0.59 to -0.13)	-0.20 (-0.45 to 0.05)	-0.11 (-0.36 to 0.15)
**Number of Acute Care Beds**	# of beds	All:	0.24* (0.05 to 0.42)	-0.01 (-0.20 to 0.19)	0.03 (-0.16 to 0.22)
		Teaching:	0.50* (0.23 to 0.76)	-0.24 (-0.54 to 0.07)	-0.03 (-0.35 to 0.29)
		Community-Large:	-0.02 (-0.24 to 0.20)	0.01 (-0.25 to 0.26)	-0.17 (-0.41 to 0.07)
**Number of Emergency Department Visits**	# of visits	All:	-0.13 (-0.37 to 0.10)	0.03 (-0.21 to 0.27)	0.04 (-0.18 to 0.27)
		Teaching:	0.17 (-0.20 to 0.55)	-0.14 (-0.53 to 0.26)	0.18 (-0.16 to 0.52)
		Community-Large:	-0.44* (-0.70 to -0.17)	0.13 (-0.20 to 0.46)	-0.20 (-0.49 to 0.09)
**Average RIW**	Average RIW	All:	0.68* (0.56 to 0.80)	0.39* (0.20 to 0.57)	0.15 (-0.04 to 0.35)
		Teaching:	0.55* (0.31 to 0.80)	0.00 (-0.31 to 0.31)	0.12 (-0.20 to 0.45)
		Community-Large:	0.76* (0.62 to 0.89)	0.53* (0.32 to 0.74)	-0.20 (-0.44 to 0.05)
**Total RIW**	Total RIW	All:	0.13 (-0.06 to 0.33)	-0.02 (-0.22 to 0.17)	0.13 (-0.06 to 0.32)
		Teaching:	0.43* (0.16 to 0.70)	-0.25 (-0.55 to 0.06)	0.11 (-0.20 to 0.41)
		Community-Large:	-0.16 (-0.40 to 0.08)	-0.06 (-0.32 to 0.19)	-0.13 (-0.39 to 0.12)
**Hospital Occupancy Rate**	% of occupancy	All:	0.09 (-0.12 to 0.29)	-0.14 (-0.37 to 0.08)	0.01 (-0.20 to 0.23)
		Teaching:	0.37^ (0.07 to 0.67)	-0.28 (-0.61 to 0.06)	0.00 (-0.34 to 0.34)
		Community-Large:	-0.12 (-0.39 to 0.14)	-0.10 (-0.41 to 0.21)	0.01 (-0.27 to 0.29)
**Patients Admitted Through the Emergency Department**	% of patients	All:	0.30* (0.13 to 0.48)	-0.11 (-0.31 to 0.08)	0.12 (-0.08 to 0.31)
		Teaching:	0.47* (0.18 to 0.75)	-0.04 (-0.41 to 0.32)	0.29^ (0.00 to 0.58)
		Community-Large:	0.39* (0.16 to 0.61)	-0.10 (-0.36 to 0.16)	0.27^ (0.03 to 0.52)

For peer review only - http://bmjopen.bmj.com/site/about/guidelines.xhtml

**Patient Days in Alternate Level of Care**	%	All:	0.23^ (0.03 to 0.43)	All:	-0.01 (-0.24 to 0.22)	All:	-0.29* (-0.50 to -0.09)
		Teaching:	0.36^ (0.06 to 0.66)	Teaching:	0.02 (-0.37 to 0.42)	Teaching:	-0.28 (-0.62 to 0.05)
		Community-Large:	0.24 (-0.04 to 0.52)	Community-Large:	0.07 (-0.27 to 0.40)	Community-Large:	-0.13 (-0.43 to 0.17)

* p less than .01; ^ p less than .05; Direction of correlation is shown as Blue (positive) and Red (negative), and intensity of cell-colouring reflects strength of correlation.
Correlation strength classification: .00-.19 very weak; .20-.39 weak; .40-.59 moderate; .60-.79 strong; .80-1.0 very strong.
RIW (Acute Care Resource Intensity Weight)

For peer review only

Table 4 shows the correlation between hospital characteristics and LOS, hospital deaths (HSMR) and readmissions. LOS was largely positively correlated (and statistically significant) with the series of eight hospital characteristics. Hospital deaths (HSMR) was largely weak to very weakly negatively correlated. Readmissions were mixed with positive and negative weak to very weak correlations. Correlations between Hospital deaths (HSMR) and readmissions with the eight hospital characteristics were largely not statistically significant (aside from patient days in alternate level of care, patients admitted through the emergency department, and average acute care RIW).

The Number of Acute Care Hospital Stays was only statistically significantly correlated with LOS (negatively weakly) in Community-Large hospitals (r= -0.36, 95% CI -0.59 to -0.13, $p < 0.01$). Teaching hospitals had a moderate positive and statistically significant correlation in the Number of Acute Care Beds and LOS (r =0.5, 95% CI 0.23 to 0.76, $p < 0.01$). The Number of Emergency Department Visits and LOS were negatively moderately correlated in Community-Large hospitals (r= -0.44, 95% CI -0.7 to -0.17, $p < 0.01$). The Average Acute Care RIW was positively strongly correlated with LOS (r= 0.68, 95% CI 0.56 to 0.8, $p < 0.01$) when assessing both hospital peer groups. With respect to hospital deaths (HSMR), the average acute care RIW was positively moderately correlated in Community-Large hospitals (r=0.53, 95% CI 0.32 to 0.74, $p < 0.01$). Total Acute Care RIW was only moderately positively correlated with LOS for Teaching hospitals (r=0.43, 95% CI 0.06 to 0.7, $p < 0.01$). Hospital Occupancy Rate was only statistically significantly correlated with LOS for Teaching hospitals (r=0.37, 95% CI 0.07 to 0.67, $p < 0.05$). With respect to hospital deaths (HSMR), a hospital's occupancy rate is very weak to weakly negatively correlated (and not statistically significant). Patients Admitted Through the Emergency Department had a positive weak to moderate correlation with LOS (Teaching hospitals r=0.47, 95% CI 0.18 to 0.75, $p < 0.01$; Community-large hospitals r=0.39, 95% 0.16 to 0.61, $p < 0.01$), and a positive weak correlation with readmissions (Teaching hospitals r=0.29, 95% CI 0 to 0.58, $p < 0.05$; Community-large hospitals r=0.27, 95% CI 0.03 to 0.52, $p < 0.05$). The percentage of Patient Days in Alternate Level of Care (a measurement of days patients spend in inpatient acute care, when unneeded, while waiting for discharge to home care or other supports are ready) had a positive weak correlation with LOS in Teaching hospitals (r=0.36, 95% CI 0.06 to 0.66, $p < 0.05$), and a weak negative correlation with readmissions for all hospitals combined (r=-0.29, 95% CI -0.5 to -0.09, $p < 0.01$).

Supplementary data files include descriptive statistics (mean/percent-change values, CIs, range of values, and number of hospitals) by indicator, facility characteristics, provincial/territorial jurisdiction, and hospital type/size, and correlation matrix scatterplots.

Discussion

In recent years, there has been growing interest in the association between hospital deaths, readmission and LOS[7]. It is logical to investigate the strength and directionality of correlation between these three components of hospital performance, and with hospital characteristics. There is wide heterogeneity in the available evidence in this research area. Aside from the natural differences across studies that narrow their scope in terms of disease or procedure-specific indicators, limited clinical settings within hospitals, and small denominator groups, even a change in the unit of analysis on the same underlying data, from patient-level data to hospital-level data, can yield disparate results[10].

For peer review only

This secondary analysis of hospital performance data aimed to provide a high-level overview of the association between hospital deaths, readmission and LOS across a majority of Teaching and Community-Large hospitals in Canada between 2013-14 and 2017-18. The classification and assignment of hospital peer groups allows for more meaningful and valid comparisons of performance of hospitals across similar structural characteristics, patient volumes, and clinical services offered. Therefore, any comparison of individual hospital performance should be restricted to within a respective peer-group. Delineating the results of this study's analyses by Teaching and Community-Large hospitals allows for a more granular interpretation of hospital performance at peer group level. Of the three outcome indicators, only with the readmissions indicator was there a statistically significant result of Community-Large hospital peer-group showing a lower peer-group average than that of the Teaching peer-group. Detailed data on eight hospital characteristics were also available in the dataset published by the data steward. As this study was exploratory in nature, we additionally included these hospital characteristics in the correlation analyses to explore any meaningful relationships with the aforementioned three main indicators, and delineated by hospital peer group type.

Our earlier research[21] established that, over time, Canadian hospitals have largely improved on in-hospital mortality; readmission rates have been trending upward; and that good or bad performance in one domain of care does not automatically reflect the same performance in other domains. What this present study aimed to add is whether a hospital's improvement or weakening performance over time, in either hospital deaths (HSMR) or readmission, had a positive or negative association on the other; our results showed that 42% of hospitals, the largest proportion across the possible outcomes, in fact decreased hospital deaths (HSMR) while increasing readmission rates. Furthermore, we added LOS to the research question as a proxy of hospital efficiency. Eight hospital characteristics showed trends in strength and directionality of correlation with hospital deaths (HSMR), readmission and LOS. As this study was exploratory in nature, in both using aggregate hospital-level data and hospital characteristics in the analyses, we did not have an explicit hypothesis on the degree of association between hospital characteristics and the three outcome indicators. We note (and continued to include in the analyses) an outlier hospital (see Figure 3) with a high Hospital Deaths (HSMR) indicator value, a long LOS, and average Readmission rate.

<u>Strengths and limitations of this study</u>

The main strengths of this study are the quality and extent of data used; all Teaching and Community-Large hospitals across Canada that had publicly-available reported performance results were included in the analysis. The 'all readmission' indicator captures, as the title suggests, all readmission to hospital within 30-days; the hospital deaths (HSMR) indicator captures ~80% of all in-hospital mortality; and the LOS indicator quantifies the mean duration across all hospitalizations. Eight diverse hospital characteristics also provided summary measures that capture numerous aspects of a hospital's performance context. While results for LOS and the eight hospital characteristics were only available for the most-recent year (2017-18), for hospital deaths (HSMR) and readmission indicators, five fiscal year data points were available to measure trend over time differences.

There are limitations in this study with respect to its generalisability beyond Canada; differences in risk-adjustment methodologies, indicator definitions and calculation methods, and hospital type/size definitions, pose challenges to make apples-to-apples comparisons across countries. However, the categorical outcomes of performance simultaneously comparing hospital deaths and readmission, along with the correlation tests of these indicators and hospital characteristics, is available and worthwhile to other settings. Community-Medium and

For peer review only

Community-Small hospitals in Canada treat fewer patients, and offer less-complex clinical services. This large group of hospitals (comprising of more than half within the country) are omitted from this study due to an absence of publicly-reported indicator values for hospital deaths. Furthermore, as a result of mergers between disparate hospitals, historic indicator values (i.e., 2013-14 data year) are omitted from the reporting platform. Thus, this inhibits a longitudinal comparison (i.e., performance trend over time). However, current indicator values and hospital characteristics data is available and was included in analyses that only required 2017-18 data year (namely, correlation analyses on hospital characteristics).

An important limitation of this study, inherent to the constraints of using aggregate-level hospital data, is the inability to perform more complex analyses. Previous, more granular analyses by researchers have been able to employ more sophisticated statistical techniques, including modelling, controlling for confounding factors, calculation of composite indicators, application of more refined case inclusion/exclusion criteria, and stratification of analyses across different disease groups. Another such example of a limitation exists with the LOS measure reflecting the average of all hospitalizations, and the inability to select just those applicable to Hospital Deaths (HSMR) or Readmission patients respectively. Acknowledging these limitations of performing secondary analyses on aggregate, publicly-available hospital performance data, we nonetheless pursued our four research questions, with the data available at hand, to determine what, if any, level of association exists at the hospital indicator level.

The two main outcome indicators themselves, Hospital Deaths (HSMR) and Readmission, also have methodological limitations due to the inability of including non-hospital death data. The Hospital Deaths (HSMR) indicator, unlike the Summary Hospital-level Mortality Indicator (SHMI), can only account for deaths that occur in hospitals. Similarly, the Readmission indicator cannot exclude patients from the denominator that have passed away in the community following hospital discharge. While the indicators of Hospital Deaths (HSMR) and Readmission are risk-adjusted (as described in the Methods section), not all risk-factors can be adjusted for (due to reasons such as viability)[22]. For example, detailed data on patient socio-demographics or access to primary care services is unavailable for risk-adjustment modelling. Lastly, as correlation does not equal causation, the correlation-based results of this study should be interpreted with caution.

Reflections on the study's findings

Public reporting of performance results poses challenges to hospital administrators and the broader public. Public reporting has become a staple in health systems and hospital performance management. But the practice of public reporting is not without concerns[23]. Tunnel vision and myopia by hospital governance and performance managers can run the risk of sub-optimisation; the unintended consequences of shifting concentration disproportionately towards areas prioritized for immediate measurement at the expense of other areas of care and broader/long-term organizational goals[24].

Pay for performance schemes are commonplace in hospital governance. A governance model that assesses hospitals through isolated performance measures, runs the risk of unintended consequences in other factors of care and performance not under immediate scrutiny[8]. The results and methods of this study support the notion that quantification of hospital performance should not be done via isolated or single measures at a time, but rather in a more broad and informed mechanism of considering complementary aspects of hospital performance (such as those in the CIHI Hospital Performance Framework: access to services, clinical effectiveness, safety, coordination of care, patient-centeredness, and hospital efficiency)[25]. Furthermore, a poorly conceptualized pay-for-performance scheme

For peer review only

may be mal-aligned to take into consideration the correlation (and potential causality) of intensifying efforts to reduce, for example, LOS or hospital mortality, on the increase of readmission rates.

Moreover, government officials charged with hospital governance must take into account inequality across hospital facilities and hospital corporations. Beginning in the 1990s, but increasing rapidly in recent years, there has been a trend of mergers between multiple hospitals and between hospitals and rehabilitation institutes into a singular hospital corporation[26]. These larger hospital corporations in turn have near-exclusive coordination of care between acute-care patients served in hospitals and subsequently their transfer to rehabilitation services. Rural and more-remote hospitals (especially those without paired rehabilitation services) could face higher LOS and occupancy rates, greater number of days and percentage of patients in alternate level of care, and greater resource utilization. If analysis of these amalgamated hospitals and rehabilitation services proves they perform better than hospitals without direct rehabilitation services, this consideration should also be included in the contextual interpretation (and perhaps risk-adjustment) of hospital performance and governance. Similarly, readmission to hospital may also be a proxy of the strength and availability of primary health care services in the community. Thus, the necessity to consider hospital performance in the broader context of an integrated health service delivery system, a tenet of the accountable care organization movement[27].

Government bodies and professional associations charged with supporting quality improvement initiatives can use the methods and findings of this type of analysis to identify best practices and top-performing hospitals so as to learn from their effective practices. Similarly, hospitals in an unfavourable quadrant (long LOS, and high hospital mortality and readmissions) should receive tailored programs to support their improvement in quality and efficiency of care.

The general public, too, requires consideration when publicly reporting performance results. Efforts in describing indicators in plain language and providing a framework for contextualization can increase the public's assimilation of performance results (especially demographic groups with fewer skills or resources)[28]. CIHI's applies these practices in their online YHS tool, providing their health system performance[29] and hospital performance frameworks[25] as a basis for the curation of performance results, and describing both performance indicators and hospital characteristics in plain language.

The results of this study do not provide a definitive outcome to the debate on the complementarity between LOS, hospital deaths, readmission and hospital characteristics. The underlying pathways and differences between hospitals in functions, and scope of services provided, makes the hospital a complex unit of analyses. The corpus of past studies illustrates the wide heterogeneity of research methods and degree of association outcomes. The embedding of this type of analysis into hospital governance formulation can only better-inform those charged with policy-making, and administrators of hospitals. Subdividing the research methods of this study, into disease and/or procedure-specific analysis, can help facilitate addressing quality improvement concerns on specific clinical areas; but caution is stressed so as to not unintentionally cause clinicians and hospital administrators to experience tunnel vision.

Conclusions

This study shows that secondary analyses of publicly-reported hospital performance results can reveal meaningful insights into the association among outcome indicators and hospital characteristics. Good or bad hospital performance in one care domain does not necessarily reflect similar performance in other care domains. Thus, caution is warranted in a narrow use of outcome indicators in the design and operationalization of hospital

For peer review only

performance measurement and governance models (namely pay-for-performance schemes). Analysis such as this can also inform quality-improvement strategies and targeted efforts to address domains of care experiencing declining performance over time; further granular subdivision of the analyses, for example by hospital peer-groups, can reveal notable differences in performance.

Contributors:

OF initially conceived of the study, reviewed the literature, performed data analysis, interpreted results, and drafted the manuscript. EM assisted in the design of the study, performed and validated data analysis, interpreted results, and reviewed the manuscript. NK assisted with the design of the study, interpreted results, and assisted in the drafting of the manuscript.

Funding:

This research received no specific grant from any funding agency in the public, commercial or not-for-profit sectors.

Competing interests:

None declared. The results and views expressed are those of the authors alone.

Patient consent for publication:

Not required.

Ethics approval:

Not required.

Provenance and peer review:

Not commissioned; externally peer reviewed.

Data availability statement:

All hospital performance and characteristics data used in this study is publicly available via CIHI's Your Health System online tool (http://yourhealthsystem.cihi.ca/).

Legend Abbreviation for Figures

For both Figures 2 and 3: LOS (Average Length of Stay)

For peer review only

References

[1] Oliver Groene, Jutta K. H. Skau, Anne Frølich; An international review of projects on hospital performance assessment, International Journal for Quality in Health Care, Volume 20, Issue 3, 1 June 2008, Pages 162–171,

[2] Helene Eckhardt, Peter Smith, Wilm Quentin. (2019). Pay for Quality: using financial incentives to improve quality of care. In R. Busse, N. Klazinga, D. Panteli, W. Quentin (Eds). Improving healthcare quality in Europe. Characteristics, effectiveness and implementation of different strategies. WHO Regional Office Europe. Copenhagen 2019, pp. 233-264.

[3] Litvak, Eugene & Bisognano, Maureen. (2011). More Patients, Less Payment: Increasing Hospital Efficiency In The Aftermath Of Health Reform. Health affairs (Project Hope). 30. 76-80. 10.1377/hlthaff.2010.1114.

[4] Shahian, D. M., Iezzoni, L. I., Meyer, G. S., Kirle, L., & Normand, S.-L. T. (2012). Hospital-wide Mortality as a Quality Metric: Conceptual and Methodological Challenges. American Journal of Medical Quality, 27(2), 112–123.

[5] Fischer C, Lingsma HF, Marang-van de Mheen PJ, Kringos DS, Klazinga NS, Steyerberg EW. Is the readmission rate a valid quality indicator? A review of the evidence [published correction appears in PLoS One. 2015;10(2):e0118968]. PLoS One. 2014;9(11).

[6] Cylus, Jonathan, Papanicolas, Irene and Smith, Peter C. (2016) Conclusions. In: Cylus, Jonathan, Papanicolas, Irene and Smith, Peter C., (eds.) Health system efficiency: How to make measurement matter for policy and management. Health Policy Series. European Observatory on Health Systems and Policies, Brussels, Belgium, pp. 225-241.

[7] Lingsma HF, Bottle A, Middleton S, et al. Evaluation of hospital outcomes: the relation between length-of-stay, readmission, and mortality in a large international administrative database. BMC Health Serv Res. 2018;18(1):116.

[8] Kaboli PJ, Go JT, Hockenberry J, et al. Associations Between Reduced Hospital Length of Stay and 30-Day Readmission Rate and Mortality: 14-Year Experience in 129 Veterans Affairs Hospitals. Ann Intern Med. 2012;157:837–845.

[9] Dharmarajan K, Wang Y, Lin Z, et al. Association of Changing Hospital Readmission Rates With Mortality Rates After Hospital Discharge. JAMA. 2017;318(3):270–278.

[10] Hofstede SN, van Bodegom-Vos L, Kringos DS, et al. Mortality, readmission and length of stay have different relationships using hospital-level versus patient-level data: an example of the ecological fallacy affecting hospital performance indicators. BMJ Quality & Safety 2018;27:474-483.

For peer review only

[11] Auger, K. A., Teufel, R. J., Harris, J. M., Gay, J. C., Del Beccaro, M. A., Neuman, M. I., ... Shah, S. S. (2017). Children's hospital characteristics and readmission metrics. *Pediatrics*, *139*(2).

[12] Paterson JM, Williams JI, Kreder HJ, et al. Provider volumes and early outcomes of primary total joint replacement in Ontario. Can J Surg. 2010;53(3):175–183.

[13] Canadian Institute for Health Information. Your Health System – In Depth [internet]. Ottawa: CIHI, 2019 https://yourhealthsystem.ca (accessed 18 November 2019).

[14] Canadian Institute for Health Information. Indicator Library [internet]. Ottawa: CIHI; 2019 https://www.cihi.ca/en/indicator-library (accessed 18 November 2019).

[15] Canadian Institute for Health Information. Your Health System: In Depth. Technical Notes for Contextual Measures (October 2019). Ottawa: CIHI; 2019.

[16] Canadian Institute for Health Information. Model specifications — clinical indicators. 2019. Ottawa: CIHI, 2019.

[17] Canadian Institute for Health Information. Hospital Standardized Mortality Ratio - Technical Notes, September 2019. Ottawa, ON: CIHI; 2019.

[18] Canadian Institute for Health Information. Indicator Library: Peer Group Methodology. Ottawa: CIHI; 2016.

[19] Canadian Institute for Health Information. HSMR: Frequently asked questions [internet]. Ottawa: CIHI; 2020 https://www.cihi.ca/en/hospital-standardized-mortality-ratio-hsmr-frequently-asked-questions#_faq22 (accessed 13 April 2020).

[20] BMJ. 11. Correlation and regression [internet]. London; BMJ; 2020 https://www.bmj.com/about-bmj/resources-readers/publications/statistics-square-one/11-correlation-and-regression (accessed 13 April 2020).

[21] Fekri O, Manukyan E, Klazinga N. Appropriateness, effectiveness, and safety of care delivered in Canadian hospitals: a longitudinal assessment on the utility of publicly reported performance trend data between 2012-2013 and 2016-2017. BMJ Open 2020; **O**:e035447. Doi:10.1136/bmjopen-2019-035447

[22] Canadian Institute for Health Information. Indicator Library: General Methodology Notes – Clinical Indicators May 2020. Ottawa. CIHI; 2020.

[23] Martin M, Davies H. Public release of information on quality of care: how are health services and the public expected to respond?, J Health Serv Res Policy, 2001, vol. 6 (pg. 158-62)

For peer review only

[24] Smith, P. (1995) On the Unintended Consequences of Publishing Performance Data in the Public Sector. International Journal of Public Administration, 18, 277-310.

[25] Canadian Institute for Health Information. A Performance Measurement Framework for Canadian Hospitals, 2013. Ottawa: CIHI; 2013.

[26] Howlett, K. Health-care providers unite in bid to improve care, cut costs. 2011; https://www.theglobeandmail.com/news/toronto/health-care-providers-unite-in-bid-to-improve-care-cut-costs/article575685/ (accessed 23 December 2019).

[27] Huynh TM, Baker GR, Bierman A, et al. Exploring accountable care in Canada: integrating financial and quality incentives for physicians and hospitals. Ottawa: Canadian Foundation for Healthcare Improvement; 2014.

[28] Hibbard, J. H., Greene, J., & Daniel, D. (2010). What Is Quality Anyway? Performance Reports That Clearly Communicate to Consumers the Meaning of Quality of Care. Medical Care Research and Review, 67(3), 275–293.

[29] Canadian Institute for Health Information. A performance measurement framework for the Canadian health system. Ottawa: Canadian Institute for Health Information; 2013.

For peer review only

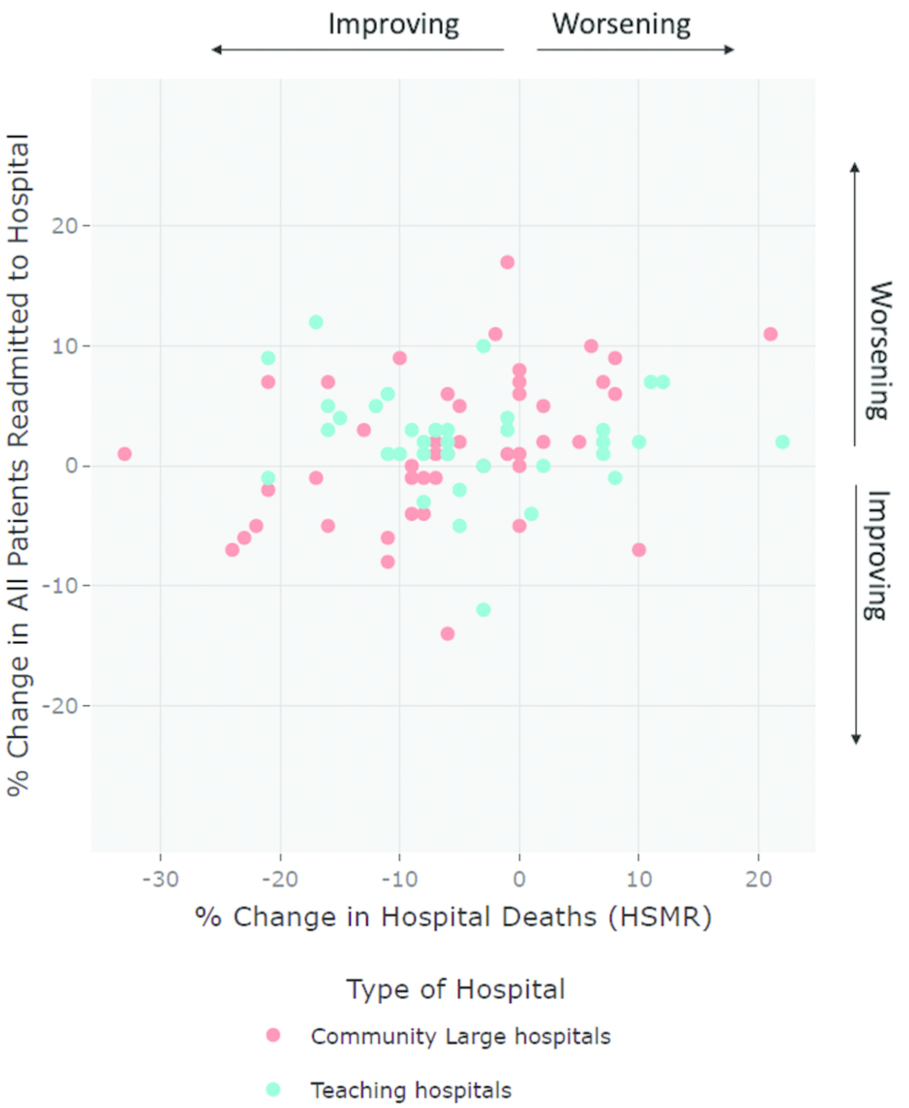

Figure 1 Scatterplot of percent change between 2013-14 – 2017-18 for Readmission and HSMR (by hospital peer-group)

87x103mm (300 x 300 DPI)

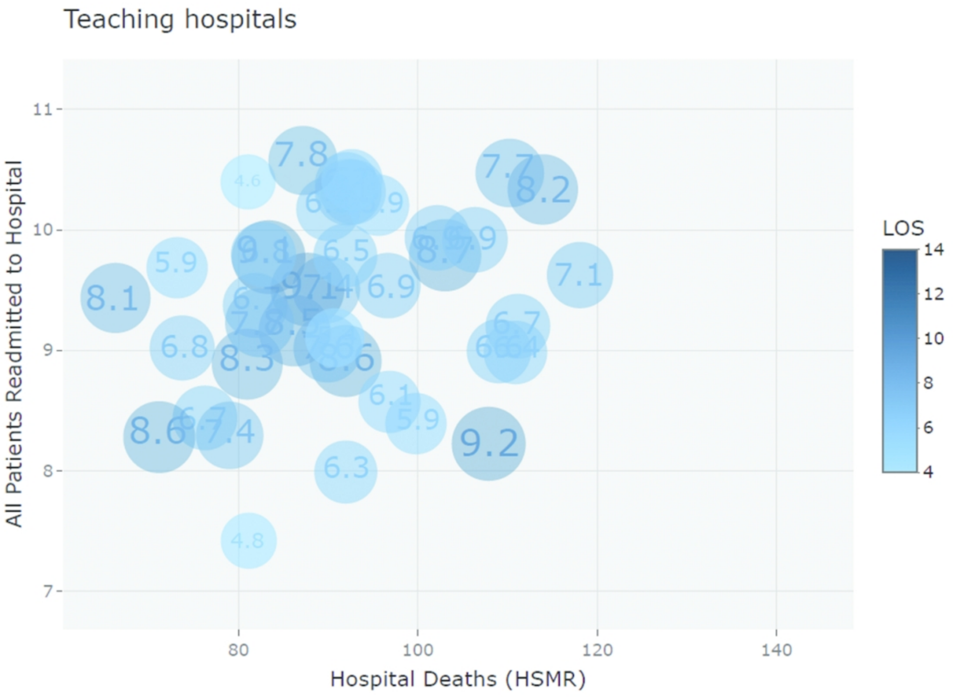

Figure 2 Scatterplot of Teaching hospital values for Hospital Deaths (HSMR), Readmission and LOS (2017-18)

119x90mm (300 x 300 DPI)

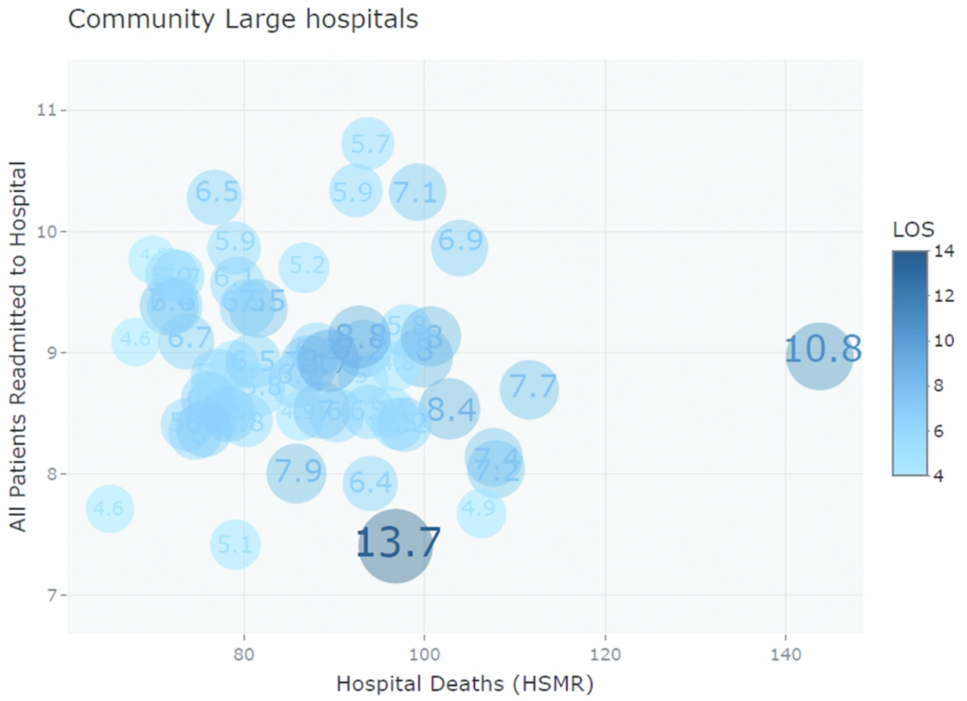

Figure 3 Scatterplot of Community-large hospital values for Hospital Deaths (HSMR), Readmission and LOS (2017-18)

119x90mm (300 x 300 DPI)

Supplementary file

Provincial/territorial range of % change difference (2013-14 vs. 2017-18), mean % change (and 95% Confidence Intervals), combined Teaching and Community-Large hospitals

Province/territory	Indicator	Range of % change (2013-14 vs. 2017-18)	Mean % change (95% CI)
Alberta	All Patients Readmitted to Hospital	-6 to 17	3.1 (-0.7 to 6.9)
	Hospital Deaths (HSMR)	-21 to 22	-0.7 (-9.3 to 7.9)
British Columbia	All Patients Readmitted to Hospital	-12 to 12	1.9 (-1.2 to 5)
	Hospital Deaths (HSMR)	-33 to 11	-6.5 (-11.4 to -1.6)
Manitoba	All Patients Readmitted to Hospital	3 to 10	6.7 (-2.1 to 15.4)
	Hospital Deaths (HSMR)	-13 to 12	-1.3 (-32.6 to 29.9)
New Brunswick	All Patients Readmitted to Hospital	-8 to 2	-3.2 (-11.2 to 4.7)
	Hospital Deaths (HSMR)	-11 to 10	-2.5 (-16.8 to 11.8)
Newfoundland and Labrador	All Patients Readmitted to Hospital	1 to 10	5.5 (-51.7 to 62.7)
	Hospital Deaths (HSMR)	-6 to 6	0.0 (-76.2 to 76.2)
Nova Scotia	All Patients Readmitted to Hospital	-4 to 11	3.5 (-91.8 to 98.8)
	Hospital Deaths (HSMR)	1 to 21	11.0 (-116.1 to 138.1)
Ontario	All Patients Readmitted to Hospital	-14 to 9	0.9 (-1 to 2.8)
	Hospital Deaths (HSMR)	-24 to 8	-5.8 (-9.2 to -2.5)
Prince Edward Island*	All Patients Readmitted to Hospital	-5 to -5	N/A
	Hospital Deaths (HSMR)	-22 to -22	N/A
Quebec	All Patients Readmitted to Hospital	1 to 9	4.8 (1.8 to 7.8)
	Hospital Deaths (HSMR)	-21 to -1	-12.0 (-19.5 to -4.5)
Saskatchewan	All Patients Readmitted to Hospital	-2 to 3	0.8 (-2.5 to 4)
	Hospital Deaths (HSMR)	-11 to -5	-7.8 (-11.7 to -3.8)

*Only one hospital value.

For peer review only

Subset of hospitals (n=81), with both Readmission and Hospital Deaths (HSMR) values, used in performance trends over time analysis

Provincial/territorial jurisdiction	Community — large hospitals	Teaching hospitals	Jurisdiction total
Alberta	4	7	11
British Columbia	11	6	17
Manitoba	1	2	3
New Brunswick	3	1	4
Newfoundland and Labrador	1	1	2
Nova Scotia	1	1	2
Ontario	21	10	31
Prince Edward Island	1	0	1
Quebec	2	4	6
Saskatchewan	0	4	4
**Total**	**45**	**36**	**81**

Facility characteristic averages by hospital peer-groups

Facility characteristic	Unit	Mean value, (n of hospitals)	
		Teaching hospitals	Community – Large hospitals
**Number of Acute Care Hospital Stays**	# of days	27,322 (n=53)	20,421 (n=66)
**Number of Acute Care Beds**	# of beds	474 (n=53)	328 (n=66)
**Number of Emergency Department Visits**	# of visits	83,441 (n=40)	86,962 (n=43)
**Average Acute Care Resource Intensity Weight (RIW)**	average RIW	1.6 (n=53)	1.2 (n=66)
**Total Acute Care RIW**	total RIW	43,295 (n=53)	25,057 (n=66)
**Hospital Occupancy Rate**	% of occupancy	88.9 (n=44)	89.9 (n=61)
**Patients Admitted Through the Emergency Department (%)**	% of patients	44.4 (n=53)	54.4 (n=66)
**Patient Days in Alternate Level of Care (Percentage)**	%	11.4 (n=43)	15.4 (n=53)

For peer review only

Correlation matrix (scatterplot) of both Teaching and Community-Large hospitals

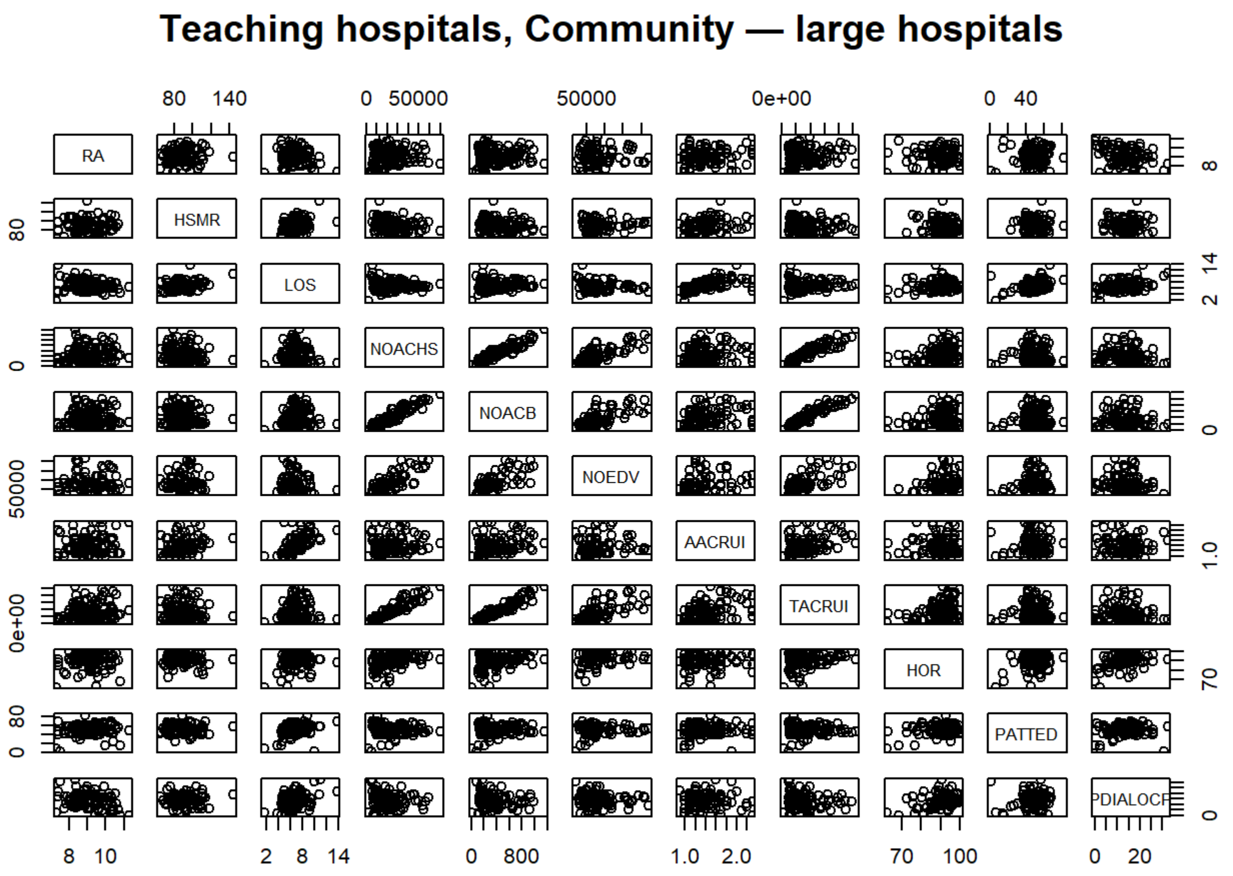

Indicator acronyms: All Patients Readmitted to Hospital (RA); Hospital Deaths (HSMR); Average Length of Stay (LOS); Number of Acute Care Hospital Stays (NOACHS); Number of Acute Care Beds (NOACB); Number of Emergency Department Visits (NOEDV); Average Acute Care Resource Intensity Weight (AACRUI); Total Acute Care Resource Intensity Weight (TACRUI); Hospital Occupancy Rate (HOR); Patients Admitted Through the Emergency Department (%) (PATTED); Patient Days in Alternate Level of Care (Percentage) (PDIALOCP).

For peer review only

<u>Correlation matrix (scatterplot) of Teaching hospitals</u>

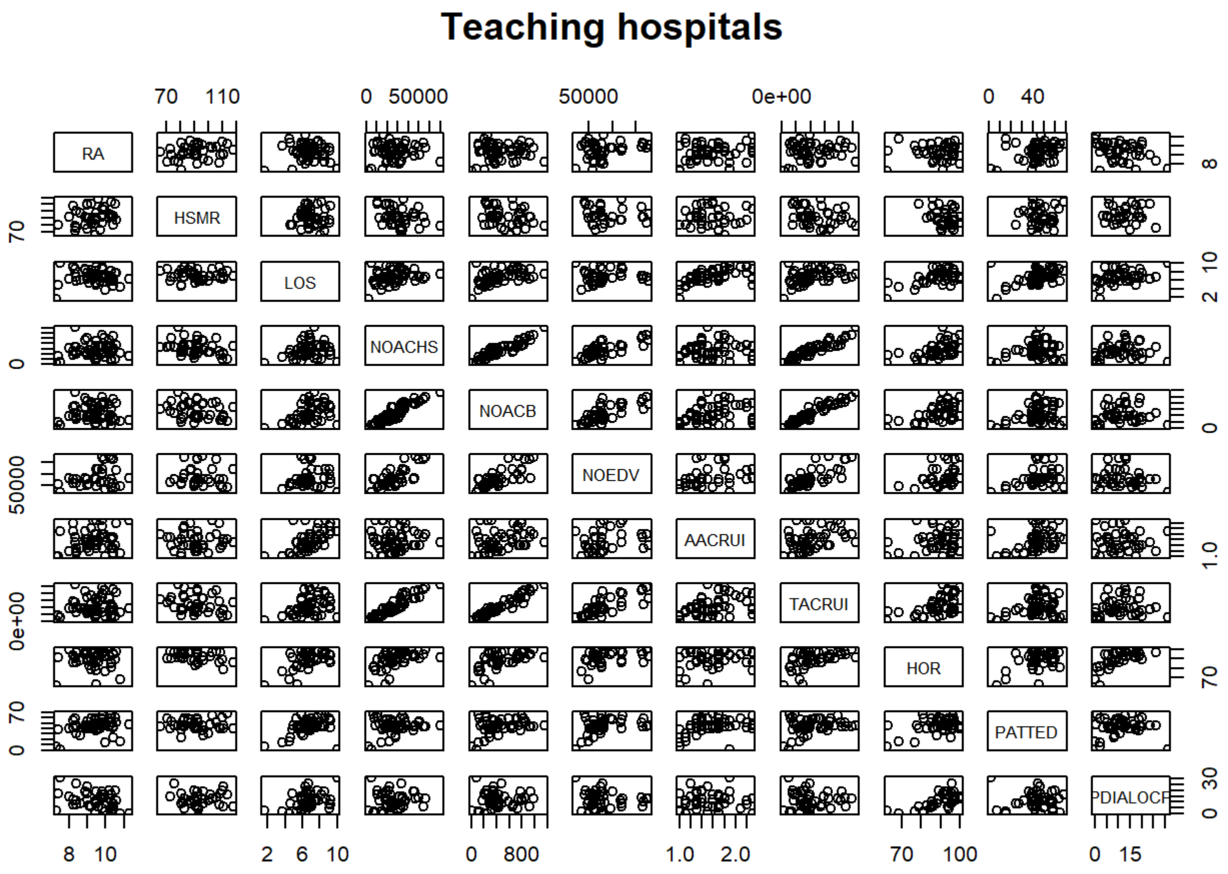

Indicator acronyms: All Patients Readmitted to Hospital (RA); Hospital Deaths (HSMR); Average Length of Stay (LOS); Number of Acute Care Hospital Stays (NOACHS); Number of Acute Care Beds (NOACB); Number of Emergency Department Visits (NOEDV); Average Acute Care Resource Intensity Weight (AACRUI); Total Acute Care Resource Intensity Weight (TACRUI); Hospital Occupancy Rate (HOR); Patients Admitted Through the Emergency Department (%) (PATTED); Patient Days in Alternate Level of Care (Percentage) (PDIALOCP).

<u>Correlation matrix (scatterplot) of Community-Large hospitals</u>

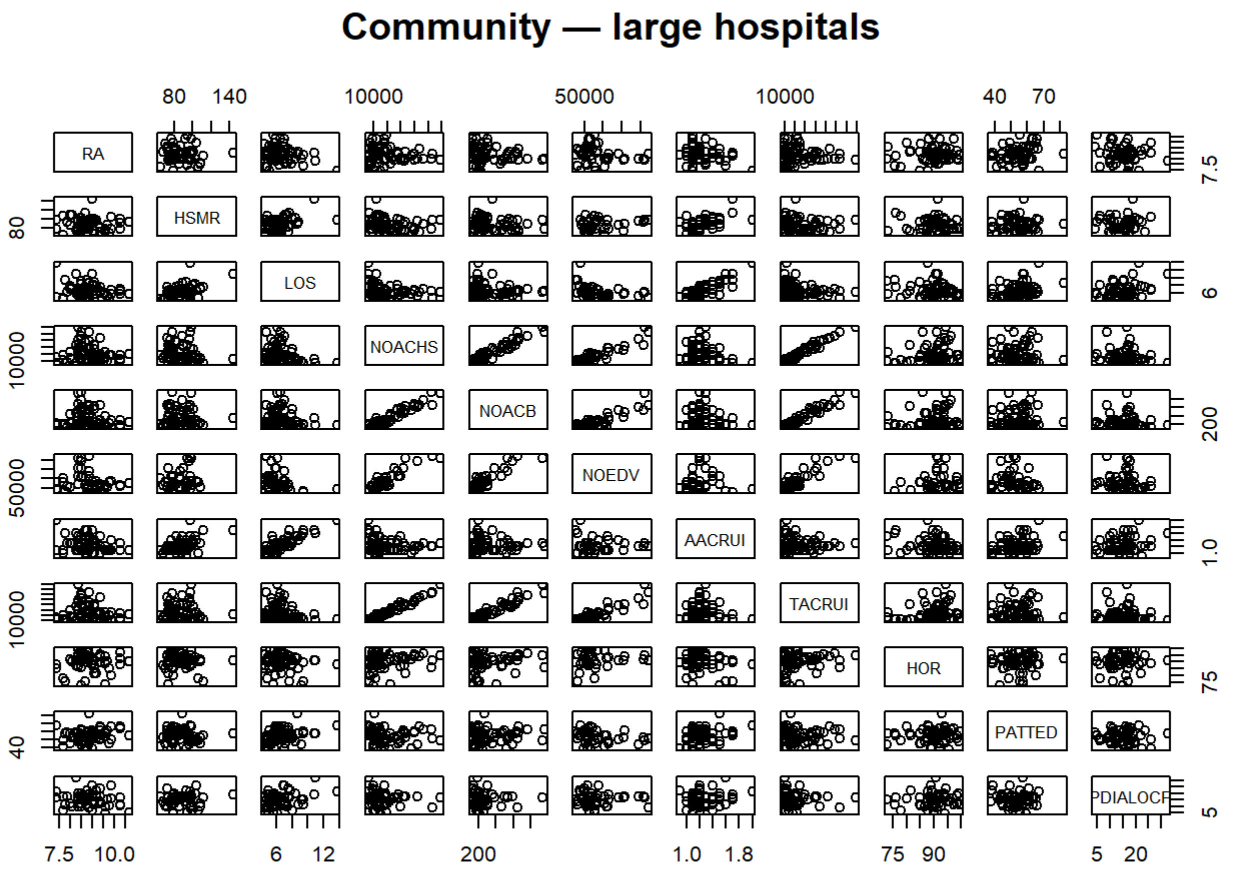

Indicator acronyms: All Patients Readmitted to Hospital (RA); Hospital Deaths (HSMR); Average Length of Stay (LOS); Number of Acute Care Hospital Stays (NOACHS); Number of Acute Care Beds (NOACB); Number of Emergency Department Visits (NOEDV); Average Acute Care Resource Intensity Weight (AACRUI); Total Acute Care Resource Intensity Weight (TACRUI); Hospital Occupancy Rate (HOR); Patients Admitted Through the Emergency Department (%) (PATTED); Patient Days in Alternate Level of Care (Percentage) (PDIALOCP).

For peer review only

The RECORD statement – checklist of items, extended from the STROBE statement, that should be reported in observational studies using routinely collected health data.

	Item No.	STROBE items	Location in manuscript where items are reported	RECORD items	Location in manuscript where items are reported
**Title and abstract**					
	1	(a) Indicate the study's design with a commonly used term in the title or the abstract (b) Provide in the abstract an informative and balanced summary of what was done and what was found		RECORD 1.1: The type of data used should be specified in the title or abstract. When possible, the name of the databases used should be included.<br><br>RECORD 1.2: If applicable, the geographic region and timeframe within which the study took place should be reported in the title or abstract.<br><br>RECORD 1.3: If linkage between databases was conducted for the study, this should be clearly stated in the title or abstract.	1.1 Noted in title and abstract.<br><br>1.2 Noted in title and abstract.<br><br>1.3. Not applicable as no linkages were performed.
**Introduction**					
Background rationale	2	Explain the scientific background and rationale for the investigation being reported			Introduction paragraphs 1-4
Objectives	3	State specific objectives, including any prespecified hypotheses			Introduction paragraph 4
**Methods**					
Study Design	4	Present key elements of study design early in the paper			Methods section
Setting	5	Describe the setting, locations, and relevant dates, including periods of recruitment, exposure, follow-up, and data collection			Methods paragraphs 1-12
Participants	6	*(a) Cohort study* - Give the eligibility criteria, and the sources and methods of selection of participants. Describe methods of follow-up<br>*Case-control study* - Give the eligibility criteria, and the sources and methods of case ascertainment and control selection. Give the rationale for the choice of cases and controls<br>*Cross-sectional study* - Give the eligibility criteria, and the sources and methods of selection of participants<br><br>*(b) Cohort study* - For matched studies, give matching criteria and number of exposed and unexposed<br>*Case-control study* - For matched studies, give matching criteria and the number of controls per case		RECORD 6.1: The methods of study population selection (such as codes or algorithms used to identify subjects) should be listed in detail. If this is not possible, an explanation should be provided.<br><br>RECORD 6.2: Any validation studies of the codes or algorithms used to select the population should be referenced. If validation was conducted for this study and not published elsewhere, detailed methods and results should be provided.<br><br>RECORD 6.3: If the study involved linkage of databases, consider use of a flow diagram or other graphical display to demonstrate the data linkage process, including the number of individuals with linked data at each stage.	6.1 N/A<br><br>6.2 N/A<br><br>6.3 N/A
Variables	7	Clearly define all outcomes, exposures, predictors, potential confounders, and effect modifiers. Give diagnostic criteria, if applicable.		RECORD 7.1: A complete list of codes and algorithms used to classify exposures, outcomes, confounders, and effect modifiers should be provided. If these cannot be reported, an explanation should be provided.	7.1 Outcomes and variables described in the Methods section paragraphs 1-7, 9, 10
Data sources/ measurement	8	For each variable of interest, give sources of data and details of methods of assessment (measurement).<br>Describe comparability of assessment methods if there is more than one group			Data source described in Methods paragraph 1

For peer review only

Bias	9	Describe any efforts to address potential sources of bias			Bias of available data described in Methods paragraph 4-6
Study size	10	Explain how the study size was arrived at			Methods paragraph 5
Quantitative variables	11	Explain how quantitative variables were handled in the analyses. If applicable, describe which groupings were chosen, and why			Groupings described in Methods paragraphs 5-7<br><br>Quantitative variables described in Methods paragraphs 8-11
Statistical methods	12	(a) Describe all statistical methods, including those used to control for confounding<br>(b) Describe any methods used to examine subgroups and interactions<br>(c) Explain how missing data were addressed<br>(d) *Cohort study* - If applicable, explain how loss to follow-up was addressed<br>*Case-control study* - If applicable, explain how matching of cases and controls was addressed<br>*Cross-sectional study* - If applicable, describe analytical methods taking account of sampling strategy<br>(e) Describe any sensitivity analyses			Methods paragraphs 8-12
Data access and cleaning methods		..		RECORD 12.1: Authors should describe the extent to which the investigators had access to the database population used to create the study population.<br><br>RECORD 12.2: Authors should provide information on the data cleaning methods used in the study.	12.1 Noted in methods section that data is publicly available for use. Also described in Data Availability Statement at conclusion of manuscript.<br><br>12.2 No data cleaning methods were used in the study.
Linkage		..		RECORD 12.3: State whether the study included person-level, institutional-level, or other data linkage across two or more databases. The methods of linkage and methods of linkage quality evaluation should be provided.	12.3 No data linkage was performed.
**Results**					
Participants	13	(a) Report the numbers of individuals at each stage of the study (*e.g.*, numbers potentially eligible, examined for eligibility, confirmed eligible, included in the study, completing follow-up, and analysed)<br>(b) Give reasons for non-participation at each stage.<br>(c) Consider use of a flow diagram		RECORD 13.1: Describe in detail the selection of the persons included in the study (*i.e.,* study population selection) including filtering based on data quality, data availability and linkage. The selection of included persons can be described in the text and/or by means of the study flow diagram.	13.1 No person-level data was used in the study. Number of hospitals included in study described in Methods paragraph 7, and Results section Table 1, and supplementary file
Descriptive data	14	(a) Give characteristics of study participants (*e.g.*, demographic, clinical, social) and information on exposures and potential confounders<br>(b) Indicate the number of participants with missing data for each variable of interest<br>(c) *Cohort study* - summarise follow-up time (*e.g.*, average and total amount)			Descriptive information on hospitals are stated in Methods section, and in Table 1 of Results section.

For peer review only

Outcome data	15	*Cohort study* - Report numbers of outcome events or summary measures over time<br>*Case-control study* - Report numbers in each exposure category, or summary measures of exposure<br>*Cross-sectional study* - Report numbers of outcome events or summary measures			Reported in Table 2 of Results section.
Main results	16	(a) Give unadjusted estimates and, if applicable, confounder-adjusted estimates and their precision (e.g., 95% confidence interval). Make clear which confounders were adjusted for and why they were included<br>(b) Report category boundaries when continuous variables were categorized<br>(c) If relevant, consider translating estimates of relative risk into absolute risk for a meaningful time period			The Results section contains three main headings (corresponding to research questions 1,2,3, with the 4th addressed concurrently).
Other analyses	17	Report other analyses done—e.g., analyses of subgroups and interactions, and sensitivity analyses			Subgroup analyses by hospital type/size are described throughout Results section, notably tables 1,2,3 & figures 1,2,3.<br><br>Jurisdictional and hospital type/size breakdowns provided in supplementary file.
**Discussion**					
Key results	18	Summarise key results with reference to study objectives			Discussion paragraphs 2-3<br>Conclusion paragraph 1
Limitations	19	Discuss limitations of the study, taking into account sources of potential bias or imprecision. Discuss both direction and magnitude of any potential bias		RECORD 19.1: Discuss the implications of using data that were not created or collected to answer the specific research question(s). Include discussion of misclassification bias, unmeasured confounding, missing data, and changing eligibility over time, as they pertain to the study being reported.	19.1 Noted under paragraphs 2-4 of Strengths & Limitations section of Discussion section.
Interpretation	20	Give a cautious overall interpretation of results considering objectives, limitations, multiplicity of analyses, results from similar studies, and other relevant evidence			Paragraphs 2-3 of Discussion section.
Generalisability	21	Discuss the generalisability (external validity) of the study results			Paragraph 5 of Discussion section.
**Other Information**					
Funding	22	Give the source of funding and the role of the funders for the present study and, if applicable, for the original study on which the present article is based			Funding statement
Accessibility of protocol, raw data, and programming code		..		RECORD 22.1: Authors should provide information on how to access any supplemental information such as the study protocol, raw data, or programming code.	22.1 Noted in Data Availability Statement, and cited in Methods section.

For peer review only

*Reference: Benchimol EI, Smeeth L, Guttmann A, Harron K, Moher D, Petersen I, Sørensen HT, von Elm E, Langan SM, the RECORD Working Committee. The REporting of studies Conducted using Observational Routinely-collected health Data (RECORD) Statement. *PLoS Medicine* 2015; in press.

*Checklist is protected under Creative Commons Attribution (CC BY) license.

For peer review only
